# Improving HER2 Diagnostics with Digital Real‐Time PCR for Ultrafast, Precise Prediction of Anti‐HER2 Therapy Response in Patients with Breast Cancer

**DOI:** 10.1002/smtd.202500599

**Published:** 2025-09-06

**Authors:** Hee‐Joo Choi, Soo Young Park, Minsik Song, Jinhyuk Chang, YoonSik Kim, Hosub Park, Chihwan David Cha, Sohyeon Yang, Nam Hun Heo, Min Ji Song, Da Sol Kim, Hayeon Kim, Minuk Kim, Jae Eun Park, Yesung Lee, EunChae Ji, Heekyoung Chung, Ilecheon Jeong, Mineui Hong, Jin‐Wu Nam, Mee‐Hye Oh, Ji‐Hye Lee, Jinwoo Seol, Hee‐Young Won, Hyun‐Woo Song, Jaewon Eom, Do Young Lee, Han Suk Ryu, Si‐Hyong Jang, Jeong‐Yeon Lee

**Affiliations:** ^1^ Department of Pathology, College of Medicine Hanyang University Seoul Republic of Korea; ^2^ Hanyang Institute of Bioscience and Biotechnology (HY‐IBB) Hanyang University Seoul Republic of Korea; ^3^ Hanyang Biomedical Research Institute (HBRI) Hanyang University Seoul Republic of Korea; ^4^ Department of Pathology Seoul National University College of Medicine Seoul Republic of Korea; ^5^ Center for Medical Innovation, Biomedical Research Institute Seoul National University Hospital Seoul Republic of Korea; ^6^ OPTOLANE Technologies Inc. Jagok‐ro, Gangnam‐gu Seoul Republic of Korea; ^7^ Department of Pathology Hanyang University Hospital Seoul Republic of Korea; ^8^ Department of Surgery Hanyang University College of Medicine Seoul Republic of Korea; ^9^ Department of Pathology Seoul National University Hospital Seoul Republic of Korea; ^10^ Clinical Trial Center Soonchunhyang University Cheonan Hospital Cheonan‐si Chungcheongnam‐do Republic of Korea; ^11^ Genome Insight Technology Inc. Yuseong‐gu Daejeon Republic of Korea; ^12^ Department of Pathology, College of Medicine Chung‐Ang University Seoul Republic of Korea; ^13^ Hanyang Institute of Advanced BioConvergence Hanyang University Seoul Republic of Korea; ^14^ Department of Life Science, College of Natural Sciences Hanyang University Seoul Republic of Korea; ^15^ Department of Pathology Soonchunhyang University Cheonan Hospital Cheonan‐si Chungcheongnam‐do Republic of Korea; ^16^ Cancer Research Institute Seoul National University Seoul Republic of Korea

**Keywords:** anti‐HER2 therapy, breast cancer, chromosome 17, copy number alteration, digital real‐time PCR, HER2 testing

## Abstract

While human epidermal growth factor receptor (HER2) has emerged as a tumor‐agnostic biomarker, standard HER2 testing for anti‐HER2 therapies using immunohistochemistry (IHC) and in situ hybridization (ISH) assays remains subjective, time‐consuming, and often inaccurate. To address these limitations, an ultrafast and precise HER2 testing method is developed using Lab‐On‐An‐Array (LOAA) digital real‐time PCR (drPCR), a fully automated digital PCR enabling real‐time absolute quantification. A multicenter study involving four independent breast cancer cohorts cross‐validates the high diagnostic accuracy of drPCR‐based HER2 assessment. Comparative analyses with artificial intelligence algorithms, next‐generation sequencing, and droplet digital PCR demonstrate that drPCR is faster, simpler, and more accurate than conventional assays for assessing HER2 status, while IHC/ISH frequently yields false positives. Importantly, in patients initially diagnosed as HER2‐positive and treated with neoadjuvant anti‐HER2 therapy, the HER2 drPCR(+)/IHC‐ISH(+) group achieves high pathological complete response rates, while HER2 drPCR(‐)/IHC‐ISH(+) cases exhibit poor treatment responses, highlighting the superior predictive accuracy of drPCR for anti‐HER2 therapy response. Additionally, drPCR identifies patients with chromosome 17 centromere abnormalities, HER2‐zero/*ERBB2* hemizygous deletion, and *ERBB2* hyperamplification who respond favorably to anti‐HER2 therapy. Collectively, these findings establish drPCR as a clinically feasible, standardized, and ultrafast HER2 testing method for improved prediction of anti‐HER2 therapy response in patients with cancer.

## Graphical abstract

This study presents a novel, clinically feasible HER2 testing method using a fully automated digital real‐time PCR platform. It enables real‐time absolute quantification of *ERBB2* copy number alterations for rapid, simple, and accurate assessment of HEgR2 status, thereby overcoming the limitations of conventional testing and improving prediction of anti‐HER2 therapy response.

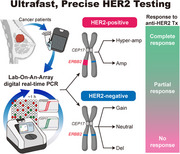

## 1 Introduction

The human epidermal growth factor receptor 2 (HER2/erbB2) is a receptor tyrosine kinase overexpressed via gene amplification in ≈15–20% of invasive breast cancers, correlating with a poor prognosis.^[^ ^1^, ^2^ ^]^ Since the first approval of trastuzumab, the continuous success of HER2‐targeted therapies has significantly improved the survival of patients with HER2‐positive breast cancer harboring *ERBB2* amplification.^[^ ^1^, ^2^ ^]^ Furthermore, the recent approval of trastuzumab deruxtecan (T‐DXd), a third‐generation anti‐HER2 antibody‐drug conjugate (ADC), for all HER2‐positive solid tumors and HER2‐low/ultra‐low breast tumor has positioned HER2 as an emerging tumor‐agnostic biomarker and therapeutic target across multiple tumors.^[^ ^3^, ^4^, ^5^ ^]^ While accurate determination of HER2 status is necessary for identifying patients who may benefit from anti‐HER2 therapies, the limitations of conventional HER2 testing methods can lead to HER2 status misclassification, resulting in inappropriate treatment and therapy resistance.

Current standard HER2 testing relies on immunohistochemistry (IHC) and in situ hybridization (ISH), which determine HER2 protein overexpression and gene amplification, respectively.^[^ ^6^ ^]^ In HER2 IHC, strong membranous staining on ≥ 10% of tumor cells (IHC score 3+) is categorized as HER2‐positive, while equivocal cases (IHC 2+) require additional ISH testing to confirm the HER2 status. Furthermore, distinguishing HER2‐zero from HER2‐low/ultra‐low within the HER2‐negative group has become essential for T‐DXd treatment.^[^ ^4^, ^7^ ^]^ Although IHC and ISH are gold standards for HER2 evaluation, their reliability has been questioned due to various issues with testing methods and interpretation.^[^ ^2^, ^8^, ^9^, ^10^ ^]^ IHC results are influenced by pre‐analytical variables, such as fixation time and storage conditions.^[^ ^11^, ^12^ ^]^ Moreover, the subjective nature of IHC interpretation leads to intra‐ and inter‐observer variability, complicating patient selection for anti‐HER2 therapies.^[^ ^13^, ^14^, ^15^, ^16^, ^17^ ^]^ While ISH testing for the detection of *ERBB2* amplification is considered relatively accurate compared to IHC in determining HER2 status,^[^ ^13^, ^16^, ^18^ ^]^ it is labor‐intensive, technically challenging, time‐consuming, and more costly than IHC.^[^ ^2^, ^10^ ^]^ For these reasons, HER2 ISH is commonly used as a confirmatory test for IHC‐equivocal cases (IHC 2+) following initial HER2 IHC testing, although HER2 status can be determined by ISH alone. However, this two‐step approach prolongs the testing time and carries a risk of HER2 status misinterpretation due to IHC inaccuracies.

To date, various quantitative assays, including mass spectrometry,^[^ ^19^, ^20^ ^]^ artificial intelligence (AI)‐based automated scoring algorithms,^[^ ^21^, ^22^ ^]^ next‐generation sequencing (NGS),^[^ ^23^, ^24^, ^25^ ^]^ and droplet digital PCR (ddPCR),^[^ ^26^, ^27^, ^28^, ^29^, ^30^, ^31^, ^32^, ^33^, ^34^ ^]^ have been proposed as novel diagnostic methods to improve HER2 testing. However, none have been approved for clinical use. Among these, ddPCR has been the most extensively studied method over the past decade for detecting *ERBB2* amplification through absolute quantification. Although previous studies have reported high concordance between ddPCR and IHC/ISH results for HER2 status,^[^ ^26^, ^27^, ^28^, ^29^, ^30^, ^31^, ^32^, ^33^, ^34^ ^]^ the clinical utility of ddPCR‐based HER2 assessment remains uncertain due to numerous challenges, including inconsistent cutoffs, variable reference controls for *ERBB2* copy number (CN) measurement across institutions, non‐standardized methods lacking multicenter validation, and, most notably, a lack of clinical evidence supporting its predictive ability for response to anti‐HER2 therapy. Furthermore, ddPCR has technical limitations, including a high contamination risk, droplet generation variability, and reliance on end‐point detection method for quantification, which may result in false positives and negatives.^[^ ^35^ ^]^ Moreover, the complex, multi‐step ddPCR workflow and the high cost of instruments and reagents restrict its clinical application.

To address the limitations of conventional HER2 testing methods, we developed a clinically feasible, ultrafast, and accurate HER2 testing approach using Lab‐On‐An‐Array (LOAA) digital real‐time PCR (drPCR), an advanced digital PCR (dPCR) platform offering real‐time absolute quantification with high speed, sensitivity, and reproducibility. In a multicenter study across four independent breast cancer cohorts, the drPCR‐based HER2 assessment demonstrated high diagnostic accuracy, effectively identifying false‐positive IHC/ISH results. We also confirmed the clinical utility of the HER2 drPCR assay in predicting the response to neoadjuvant anti‐HER2 therapy. Given its advantages over conventional assays, we propose a new HER2 testing algorithm that applies drPCR as an alternative or complementary method to ISH/IHC to improve diagnostic accuracy for HER2‐targeted therapies.

## 2 Results

### 2.1 Overview of a Novel drPCR System

**Figure 1:**
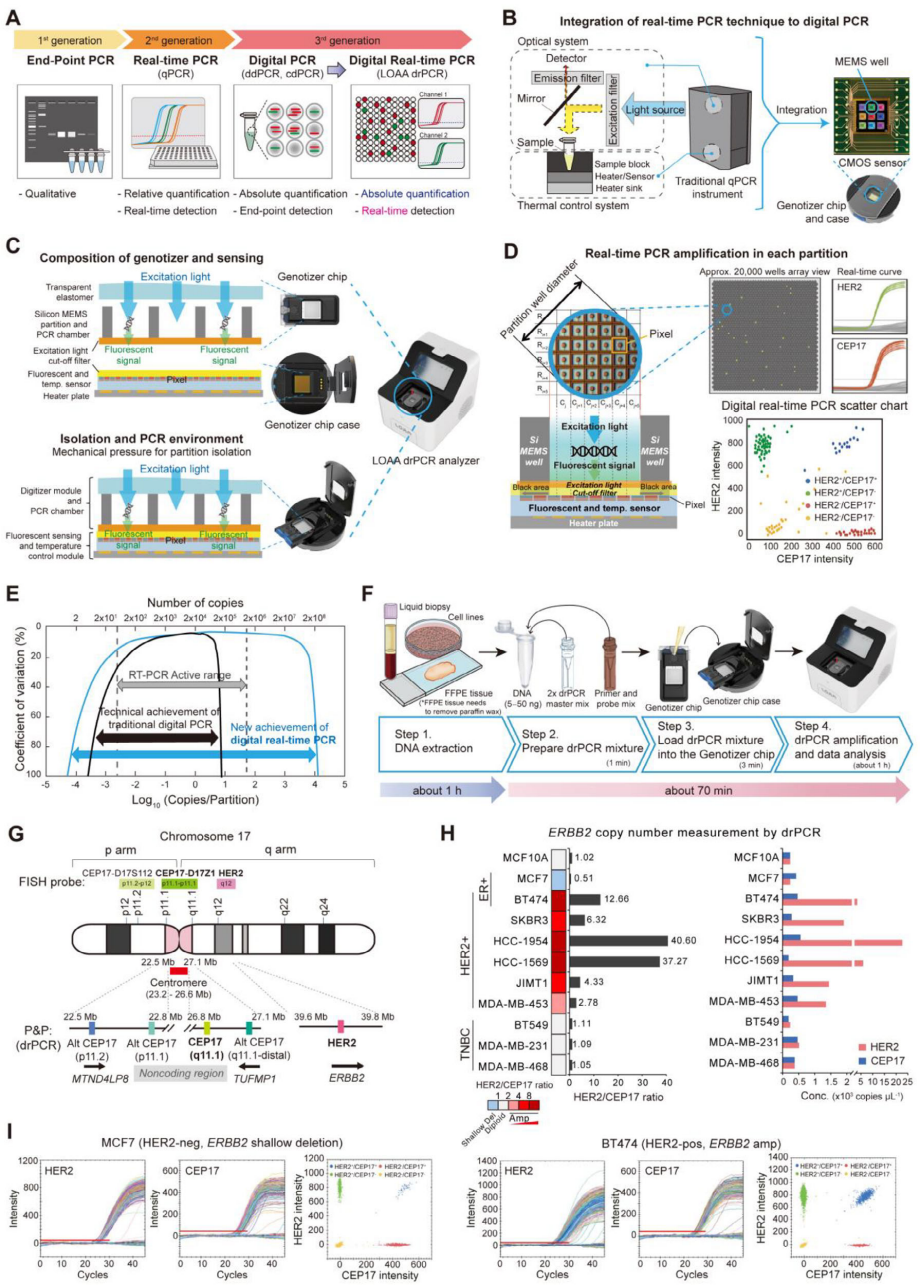
Development of a method for LOAA drPCR‐based HER2 CNA measurement. A) Overview of PCR technology development, illustrating the advanced features of LOAA drPCR, which enables absolute quantification with real‐time measurements. cdPCR, chip‐based dPCR. B) Structure of MEMS nanoparticle wells on a CMOS sensor chip in the LOAA drPCR system. C) LOAA drPCR equipment includes a Genotizer chip, chip case, and LOAA analyzer (right). The vertical structure of the Genotizer chip and chip case is shown (left). When the chip case cover is closed, all partitions of the Genotizer chip become completely isolated. temp., temperature. D) Principles of digitization and real‐time PCR analysis by fluorescent sensor structures in ≈20000 segmented wells on a Genotizer chip. Si, silicon; temp., temperature. E) High dynamic range of LOAA drPCR. F) Workflow of drPCR analysis for DNA quantification. G) Chromosomal location of the designed primer and probe sets (P&P) targeting HER2 gene and reference controls (one CEP17 and three Alt CEP17s at different loci). Alt, alternative. H,I) HER2 CNA analysis using LOAA drPCR in the indicated cell lines. HER2/CEP17 ratio (left) and concentration (Conc.; right) of HER2 and CEP17 measured by drPCR H). Representative images of real‐time PCR amplification curve and scatter plot for drPCR analysis I). Amp, amplification; HER2‐neg, HER2‐negative; HER2‐pos, HER2‐positive.

To overcome the limitations of current HER2 testing methods, we sought to develop a novel HER2 diagnostic approach using our recently launched LOAA drPCR, an advanced dPCR technology that enables ultrafast real‐time absolute quantification (**Figure** **1**A). dPCR, a third‐generation PCR technology allowing absolute quantification of nucleic acids, has emerged as a promising molecular diagnostic tool.^[^ ^35^ ^]^ However, current dPCR platforms, including droplet‐ and chip‐based types, still adopt end‐point detection methods to determine amplification‐positive and negative partitions for absolute quantification, despite the risk of false‐positive and negative results.^[^ ^36^ ^]^ This limitation arises from the technical challenges of distinguishing changes in individual fluorescence signals during real‐time measurement in each partition. Although some dPCR platforms with real‐time monitoring capabilities have been developed, including the commercially available BioMark HD (Fluidigm) and two recently reported in‐house systems,^[^ ^37^, ^38^ ^]^ they still face several limitations, such as low partition numbers, lack of system integration, or prolonged assay times, without substantial improvement in dynamic range compared to conventional end‐point dPCR platforms (Table S1, Supporting Information). To develop a more advanced real‐time dPCR system with enhanced sensitivity and accuracy, broader detection range, and reduced assay time, we introduced an innovative technology that enables PCR amplification through silicon microelectromechanical system (MEMS) nanoparticle wells on a complementary metal‐oxide semiconductor (CMOS)‐based semiconductor fluorescent sensor chip (Figure 1B).

LOAA drPCR is a fully automated instrument comprising a CMOS‐integrated chip with ≈20000 partitions, a chip case, and a drPCR analyzer (Figure 1B,C; Table S1, Supporting Information). The MEMS structure of the chip enables uniform partitioning of samples, eliminating user‐to‐user variability. The MEMS partition arrays are near the optical sensor, ensuring that each partition responds only to the internal photosensor array (Figure 1B,C). The row and column addresses of the CMOS sensor array are automatically assigned to each well segment by a software program using sensing data from dark and bright MEMS well shadow patterns formed on the CMOS sensor by excitation light illumination. This process allowed for the measurement of fluorescence signal changes during each PCR cycle, generating ≈20 000 real‐time amplification curves (Figure 1D). These real‐time amplification curves enable downstream analyses that are not feasible with conventional end‐point dPCR, such as high‐resolution melting analysis of PCR amplicons and high dynamic range (HDR) quantification using C_T_ values. This analytical flexibility significantly expands the applicability of the platform, making it highly suitable for a wide range of research and clinical applications, including highly sensitive detection of rare mutations and DNA methylation analysis (Table S1, Supporting Information).

Unlike conventional quantitative PCR (qPCR), where the optical system and thermal controller are separate,^[^ ^39^ ^]^ LOAA drPCR amplifies genes by controlling electrical signals with thermal and optical sensors embedded in the CMOS sensor chip, thereby enhancing signal detection efficiency (Figure 1B,C). According to the principle of the solid angle, fluorescence intensity decreases with greater distance from the sensor due to signal dispersion. To maximize the sensor's ability to detect fluorescence, the LOAA drPCR system incorporates a bioreaction chamber positioned directly above the optical sensor (Figure 1C; Figure S1A, Supporting Information). This unique sensing structure enables highly sensitive detection of extremely low DNA concentrations (Figure S1B, Supporting Information). Furthermore, real‐time monitoring of drPCR extended the detection ranges up to 10^8^, surpassing the typical 10^5^ detection limit of conventional dPCR (Figure 1E; Table S1, Supporting Information). The LOAA drPCR also supports multiplexing with two fluorescent channels. Each pixel of the semiconductor sensor was implemented with two different filter systems, allowing for separate output of two fluorescent dyes (Figure 1D; Figure S1C, Supporting Information).

Unlike ddPCR, which requires a separate sample partitioning step, the LOAA drPCR system automates the entire process after simply loading the PCR mixture onto the chip. This streamlined workflow achieves a turnaround time of ≈1 h (Table S1, Supporting Information), which is significantly faster than NGS or ddPCR for HER2 copy number alteration (CNA) analysis (Figure 1F; Figure S2, Supporting Information). Collectively, these features demonstrate the advanced technology and superior performance of LOAA drPCR compared to conventional dPCR systems.

### 2.2 Development of a drPCR Method for HER2 CNA Analysis

To develop a drPCR‐based HER2 CNA detection method, we designed primer‐probe sets (P&Ps) targeting *ERBB2* and reference DNA regions (Figure 1G; Table S2, Supporting Information). The optimal *ERBB2* P&P region was selected to exclude hot‐spot point mutation sites found in human cancers (Figure S3A, Supporting Information). In the standard HER2 ISH assay, CEP17 (chromosome 17 centromere) is the only approved reference probe for detecting *ERBB2* amplification. Nevertheless, most ddPCR studies assessing *ERBB2* amplification have used alternative reference genes, such as *EFTUD2*, *EIF2C1*, *TFF3*, and *RPP30*,^[^ ^26^, ^30^, ^31^, ^32^, ^33^ ^]^ due to challenges in designing specific PCR amplicons within the CEP17 probe region (17p11.1‐q11.1; the centromeric/pericentromeric region of chromosome 17). While some studies used CEP17 as a reference,^[^ ^28^, ^29^ ^]^ we found that the primer sequences were not specific. Given the importance of an accurate reference control for HER2 CNA analysis, we selected the clinically validated CEP17 as a reference control for drPCR‐based HER2 CNA assay. By avoiding AT‐rich tandem repeats and other highly repetitive sequences, we successfully designed four specific reference P&Ps within the 17p11.1‐q11.1 or 17p11.2 region (Figure 1G; Figure S3B, Supporting Information). The P&P closest to the centromeric q arm (CEP17q11.1) was used as the main reference, while three others were designated as alternative references (Figure 1G; Table S2, Supporting Information). After verifying the specificity of P&P and the absence of interference between HER2 and CEP17 P&Ps (Figure S3C–E, Supporting Information), the HER2/CEP17 duplex drPCR assay was further tested in normal and malignant breast cell lines harboring different HER2 CNAs. The drPCR assay detected low to high levels of *ERBB2* amplification, as well as *ERBB2* CN deletion in these cells, consistent with the Cancer Cell Line Encyclopedia (CCLE) data (Figure 1H,I; Figure S3F, Supporting Information), demonstrating that the drPCR conditions for HER2 CNA detection were optimized.

### 2.3 Multicenter Clinical Study for drPCR‐based HER2 Status Assessment

Next, we designed a multicenter, retrospective study for evaluating the clinical reliability of drPCR‐based HER2 status assessment in patients with breast cancer (Figure S4, Supporting Information). To establish and standardize the drPCR assay as a novel HER2 testing method, a total of 363 patients from three independent institutions were divided into a training cohort and two validation cohorts. Clinicopathological features of these patients are summarized in Table S3 (Supporting Information).

The training cohort (*n* = 103) from Soonchunhyang University Cheonan Hospital (SCHH) was assigned to develop the method for drPCR‐based HER2 assessment in clinical specimens. Fluorescence in situ hybridization (FISH) and drPCR were conducted on all cases to determine optimal cutoff for drPCR assay, and NGS was performed on 40 representative samples to ensure the accuracy of drPCR assay. To validate the drPCR‐based HER2 testing method, 200 patients from Seoul National University Hospital (SNUH; Validation set 1) and 60 patients from Chonnam National University Hwasun Hospital (CNUH; Validation set 2) were enrolled. In these cohorts, drPCR results were compared with HER2 status from clinical records based on standard HER2 testing methods. Discrepant cases were re‐evaluated using IHC, FISH, silver‐enhanced in situ hybridization (SISH), and NGS. Additionally, ddPCR and drPCR results were compared for 166 representative samples.

A separate cohort of 35 HER2‐positive patients (Hanyang University Hospital, HYUH, *n* = 26; SCHH, *n* = 9) who received neoadjuvant therapy was assigned to assess the predictive ability of drPCR for response to anti‐HER2 treatment. Biopsy specimens from these patients were analyzed using drPCR, and treatment response was compared between groups with HER2‐positive and HER2‐negative status by drPCR.

### 2.4 Establishing drPCR for HER2 Status Assessment in Patients with Breast Cancer

**Figure 2:**
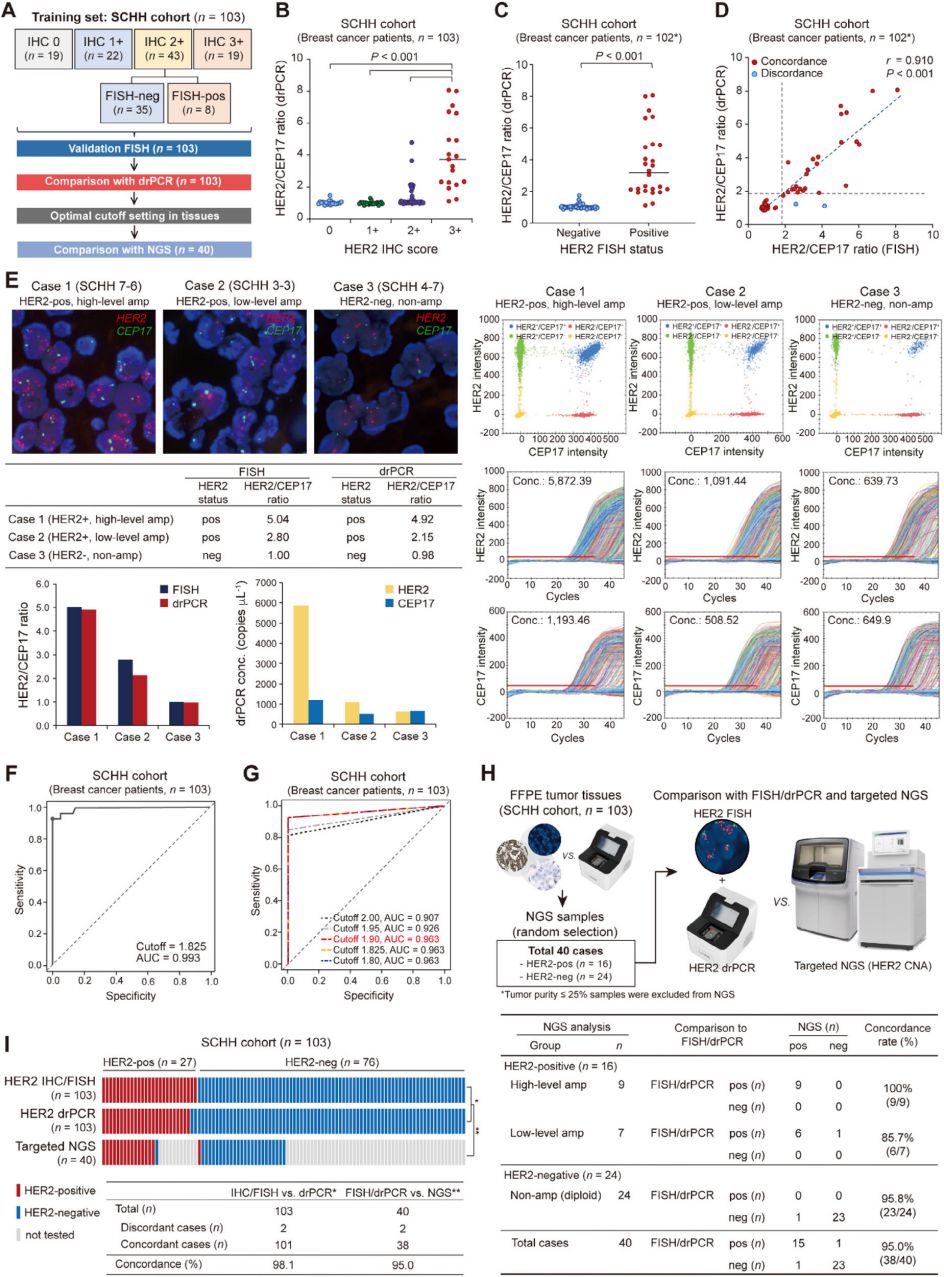
Establishment of HER2 drPCR assay in breast tumor tissues from a training cohort. A) Overview of the training cohort composition (*n* = 103) and research workflow. B,C) Distribution of HER2/CEP17 ratios measured by drPCR across HER2 IHC grades (B, total *n* = 103; IHC 0, *n* = 19; IHC 1+, *n* = 22; IHC 2+, *n* = 43; and IHC 3+, *n* = 19) and FISH results (C, total *n* = 102; FISH‐negative, *n* = 76; FISH‐positive, *n* = 26; *Out of 103 cases, one was excluded due to failure of FISH signal detection). Each dot represents an individual patient. *P*‐values were calculated using one‐way ANOVA with Tukey's post‐hoc test (B) or unpaired Student's *t*‐test C). D) Scatter plot showing the correlation between HER2/CEP17 ratios from drPCR and FISH (*n* = 102), analyzed using Pearson's correlation. E) Representative cases showing high concordance between FISH and drPCR results. FISH images (left) and drPCR scatter plots with real‐time amplification curves (right) are shown. HER2 signals, red; CEP17 signals, green; DAPI, blue in the FISH images. HER2/CEP17 ratios measured by FISH and drPCR are displayed, along with HER2 and CEP17 concentrations (conc., copies µL^−1^) from drPCR. F,G) ROC curves for determining the optimal drPCR cutoff using continuous F) and categorical G) values in the SCHH cohort (*n* = 103). H) Comparison of HER2 status using drPCR, FISH, and targeted NGS in 40 representative cases. The workflow is depicted (upper), and the table shows concordance rate (lower). I) Heatmap comparing HER2 status across IHC/FISH, drPCR, and NGS, with concordance rates displayed in a table.

To develop a clinically feasible drPCR‐based method for assessing HER2 status, 103 clinical specimens from the training cohort (SCHH) were tested. In all cases, both FISH and drPCR assays were performed concurrently to determine an optimal cutoff value for drPCR. The reliability of the drPCR results was further validated using NGS (**Figure** **2**A).

Preliminary testing of the drPCR assay in a small number of HER2‐positive and HER2‐negative patients showed reliable results with high reproducibility in the triplicate measurements, yielding a coefficient of variation (CV) of 2.41 ± 1.22% (Figure S5A, Supporting Information). A genomic DNA (gDNA) input of 25 ng per drPCR reaction was sufficient, as higher inputs did not affect the HER2/CEP17 ratio (Figure S5B, Supporting Information). When drPCR testing was expanded to all 103 cases, the drPCR‐based HER2/CEP17 ratios were statistically significantly increased in the groups with higher HER2 IHC scores or FISH‐positive status (*P* < 0.001; Figure 2B,C). The HER2/CEP17 ratios calculated using HER2 FISH and drPCR showed a strong correlation (*r* = 0.910; Figure 2D,E).

Next, we determined the optimal HER2/CEP17 cutoff for defining HER2‐positivity based on drPCR. To this end, we first aimed to set a potential cutoff range based on ROC curve analysis in a training cohort (*n* = 103), in which FISH and drPCR results were compared across all samples, and subsequently validated the candidate cutoffs in two independent institutions (validation set 1, *n* = 200; validation set 2, *n* = 60) (Figure S5C, Supporting Information). In the ROC curve analysis of training cohort, the calculated drPCR cutoff using continuous variables was 1.825 (area under the curve, AUC = 0.993; Figure 2F). Among five evaluated cutoffs ranging from 1.8 to 2.0, three values (1.8, 1.825, and 1.9) demonstrated equally high diagnostic performance (AUC = 0.963), whereas higher cutoffs (1.95 and 2.0) showed decreased AUCs (Figure 2G; Figure S5D, Supporting Information). Based on these results, we conservatively selected 1.9 as the potential final cutoff for drPCR‐based HER2‐positivity and considered HER2/CEP17 ratios between ≥1.8 and <1.9 as a gray zone requiring further evaluation (Figure S5C–E, Supporting Information). In the training cohort with marginal *ERBB2* gain/amplification (HER2/CEP17 ratios from 1.3 to 2.5), all 14 cases showed drPCR ratios either below 1.8 or above 1.9, which were clearly confirmed them as HER2‐negative or ‐positive, respectively, by FISH analysis. None of the cases fell within the gray zone, further supporting the validity of 1.9 cutoff (Figure S5E, Supporting Information). At the 1.9 cutoff, drPCR achieved 98.1% concordance with standard HER2 testing results, with 92.6% sensitivity, 100% specificity, 100% positive predictive value (PPV), and 97.4% negative predictive value (NPV) (Table S4, Supporting Information).

We further verified the drPCR results with targeted NGS in 40 representative cases, yielding 95% concordance between NGS and FISH/drPCR in assessing HER2 status. In the two discordant cases, NGS could not detect low‐level *ERBB2* amplification or led to false‐positive data (Figure 2H,I). These data supported the higher accuracy of drPCR compared to that of NGS in measuring HER2 CNA.

### 2.5 Validation of drPCR Accuracy for HER2 Status Assessment in Two Independent Cohorts

**Figure 3:**
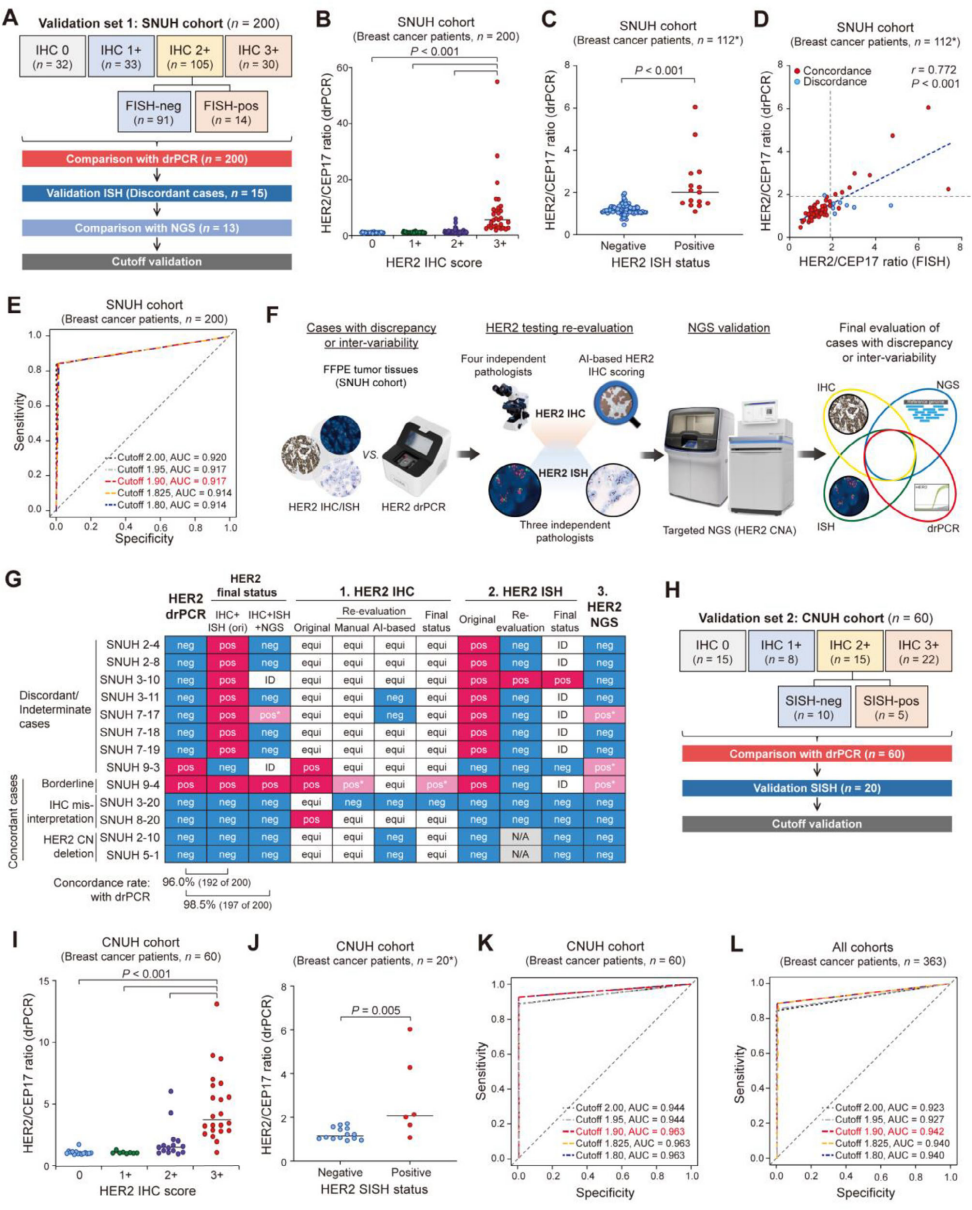
Diagnostic accuracy of drPCR‐based HER2 assessment in validation cohorts. A) Workflow for determining the accuracy of drPCR‐based HER2 assessment in validation cohort 1 (SNUH, *n* = 200). B,C) Comparison of HER2 IHC grades (B, total *n* = 200; IHC 0, *n* = 32; IHC 1+, *n* = 33; IHC 2+, *n* = 105; and IHC 3+, *n* = 30) or ISH results (C, total *n* = 112; ISH‐negative, *n* = 97; ISH‐positive, *n* = 15) with HER2/CEP17 ratios measured by drPCR. *In addition to 105 cases with an IHC score of 2+, ISH was also performed in 7 additional cases with other IHC scores. *p‐*values by one‐way ANOVA with Tukey's post‐hoc test B) or unpaired Student's *t*‐test C). D) Scatter plot showing the correlation between ISH and drPCR results for HER2/CEP17 ratios (*n* = 112) analyzed using Pearson's correlation. E) ROC curve with AUC score validating the cutoff for drPCR‐based HER2 assessment using categorical values. F) Workflow for comparative re‐evaluation of HER2 status in indeterminate or discordant cases between IHC/ISH and drPCR results. G) Heatmap showing the re‐evaluation results from each method. pos, positive; neg, negative; equi, equivocal; ID, indeterminate; N/A, not applicable. pos*, ambiguous‐positive. H) Workflow to verify the accuracy of drPCR‐based HER2 assessment in validation cohort 2 (CNUH, *n* = 60). I,J) Comparison of HER2/CEP17 drPCR results with HER2 IHC (I, total *n* = 60; IHC 0, *n* = 15; IHC 1+, *n* = 8; IHC 2+, *n* = 15; and IHC 3+, *n* = 22) and SISH results (J, total *n* = 20; SISH‐negative, *n* = 14; SISH‐positive, *n* = 6). *ISH was performed in 15 cases with an IHC score of 2+ and in additional 5 cases with other IHC scores. *p*‐values by one‐way ANOVA with Tukey's post‐hoc test I) or unpaired Student's *t*‐test J). K,L) ROC curves validating the drPCR cutoff for HER2 assessment in validation cohort 2 (K, *n* = 60) and all three cohorts (L, *n* = 363).

The diagnostic accuracy of the drPCR‐based HER2 assessment was further validated in two independent cohorts. In validation cohort 1 (SNUH, *n* = 200), which included a higher proportion of HER2 IHC‐equivocal cases (52.5%, 105/200), drPCR results were compared with prior diagnoses based on HER2 IHC/ISH analyses. Discrepant cases were re‐evaluated through additional assays, including ISH and NGS (**Figure** **3**A). Among these patients, HER2/CEP17 ratios measured by drPCR showed statistically significant differences across HER2 IHC grades and between ISH‐positive and ISH‐negative groups (*p* < 0.001; Figure 3B,C), exhibiting a strong positive correlation with those determined by the ISH assay (*r* = 0.772; Figure 3D). At a drPCR cutoff of HER2/CEP17 ratio 1.9, the diagnostic accuracy of drPCR was 96% (AUC = 0.917), with 84.1% sensitivity, 99.4% specificity, 97.4% PPV, and 95.7% NPV (Figure 3E; Figure S5D and Table S4, Supporting Information). Compared with other candidate cutoffs, the AUC value tended to be slightly higher when the cutoffs were ≥ 1.9 (Figure 3E; Figure S5C,D, Supporting Information), confirming that cutoff 1.9 was more reliable than 1.8 or 1.825.

In the SNUH cohort, eight cases showed discordance between drPCR and standard HER2 testing methods (Table S5, Supporting Information). For discordant or ambiguous cases in interpreting the results, HER2 status was re‐evaluated by independent pathologists, AI‐based IHC scoring algorithm, or NGS (Figure 3F). Among the eight discrepant cases, seven showed concordant results between NGS and drPCR, and six had revised interpretations in repeat ISH that were identical to the drPCR results (Figure 3G; Table S5, Supporting Information). NGS analysis of additional concordant cases (*n* = 5) also supported the accuracy of drPCR in assessing HER2 status. Overall, considering re‐evaluated HER2 interpretations by NGS and IHC/ISH, the accuracy of HER2 drPCR increased to 98.5% (197/200, concordant cases), suggesting high reliability of the drPCR assay compared to that of ISH, which can exhibit intra‐ and inter‐observer variability in assessing HER2 status.

We further investigated the accuracy of drPCR‐based HER2 assessment in a second validation cohort (CNUH, *n* = 60) comprising 45% HER2‐positive (27/60) and 55% HER2‐negative patients (33/60). SISH was performed for cases with equivocal IHC results or those requiring further evaluation (Figure 3H). Consistent with the results from SCHH and SNUH cohorts, drPCR showed high concordance with IHC and/or SISH results (Figure 3I,J), achieving 96.7% accuracy, 92.6% sensitivity, and 100% specificity at the drPCR cutoff 1.9 (AUC = 0.963; Figure 3K; Figure S5D; Table S4, Supporting Information). In this cohort, cutoffs ≤ 1.9 yielded higher AUCs than those of 1.95 or 2.0 (Figure 3K; Figure S5C,D, Supporting Information).

In the ROC curve analysis of 363 patients across three independent cohorts, the drPCR cutoff 1.9 was verified as optimal, yielding the highest AUC (0.942) with 88.8% sensitivity, 99.6% specificity, 98.9% PPV, 96% NPV, and 96.7% accuracy (Figure 3L; Figure S5C,D,F, and Table S4, Supporting Information). Additionally, among the 363 cases across all three institutions, only one case exhibited a drPCR ratio of 1.89 within the gray zone and was further confirmed to be HER2‐negative by both IHC and ISH analyses (Figure S5G, Supporting Information), supporting the reliability of the 1.9 cutoff. Upon correction of ambiguous ISH results through repeat ISH and NGS analysis, the sensitivity, specificity, and accuracy of drPCR increased to 94.6%, 100%, and 98.6%, respectively (Table S4, Supporting Information). Collectively, these data suggest the high diagnostic accuracy of drPCR with a validated cutoff of 1.9 in assessing HER2 status across institutions, providing a novel, standardized method for improved HER2 testing.

### 2.6 Comparison of drPCR and ddPCR for Measuring HER2 CNA

Next, we compared the performance of drPCR with ddPCR, the most widely used dPCR system,^[^ ^35^ ^]^ for measuring HER2 CNA in 166 representative clinical samples with varying *ERBB2* CN levels. When using our P&Ps, the ddPCR assay showed 98.2% concordance with drPCR results (163/166; Table S6, Supporting Information), with a strong positive correlation between the HER2/CEP17 ratios obtained by both methods (*r* = 0.984, *p* < 0.001; Figure S6A, Supporting Information). In both HER2‐positive and –negative groups, drPCR and ddPCR yielded comparable HER2/CEP17 ratios (Figure S6B, Supporting Information), suggesting that the 1.9 cutoff for drPCR could also be applied to ddPCR. However, in a HER2‐positive case with extremely high *ERBB2* amplification, ddPCR could not adequately measure the saturated *ERBB2* CN in an automated setting, requiring parameter adjustments or DNA dilution. Meanwhile, drPCR accurately quantified these high *ERBB2* CN levels without additional PCR adjustments. Moreover, all three discrepant cases were classified as HER2‐positive with low‐level *ERBB2* amplification, as confirmed by both IHC/ISH and drPCR, but were misclassified as HER2‐negative by ddPCR (Figure S6C, Supporting Information). Since the ddPCR ratios in these cases ranged from 1.8 to 1.89, marginally below the 1.9 cutoff, we further evaluated these discordant cases by replicate measurements of both ddPCR and drPCR. Repeated assays (five to six replicates per case) demonstrated that drPCR consistently showed concordant results, while ddPCR produced variable outcomes with inconclusive HER2 classification and higher CV% compared to drPCR (Figure S6C, Supporting Information). These findings support the greater reproducibility of the fully integrated drPCR platform over ddPCR, which carries potential risks of droplet variability and technical errors due to its multistep workflow. Collectively, these data suggest that drPCR is more sensitive and reproducible than end‐point detection‐based ddPCR for measuring CNA with superior advantages of a simpler workflow and faster turnaround time (Table S6 and Figures S2, S6, Supporting Information).

### 2.7 Detection of HER2 IHC False Positives by AI‐based Re‐Evaluation and drPCR

**Figure 4:**
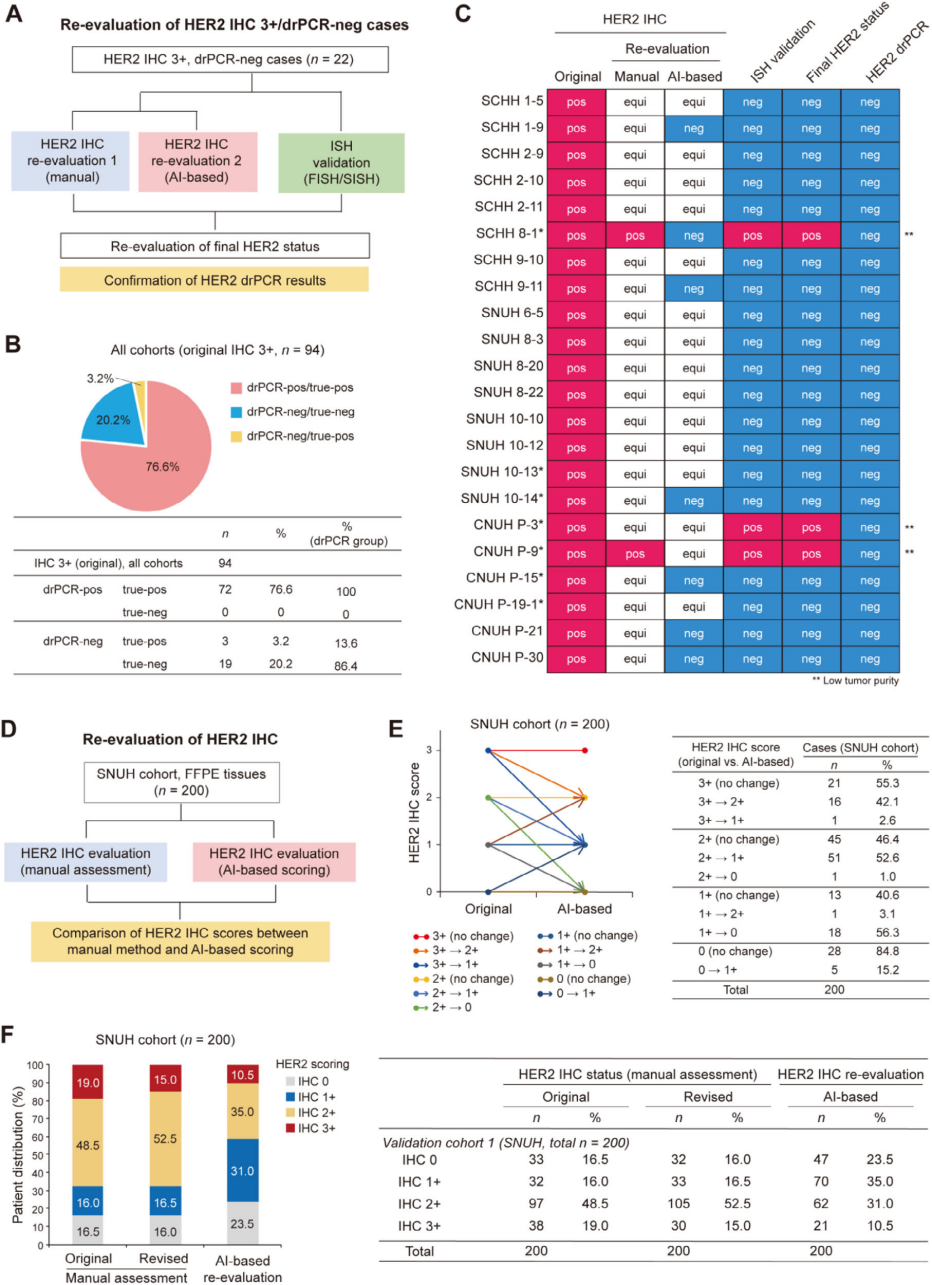
AI‐based re‐evaluation of HER2 IHC results to confirm drPCR accuracy. A) Workflow for re‐evaluating HER2 IHC 3+/drPCR‐negative cases (*n* = 22) across all three cohorts. B) Proportion of true‐positive and false‐positive HER2 IHC cases among patients with original HER2 IHC score of 3+ (*n* = 94) as determined by drPCR and ISH analyses in combination with re‐evaluation of IHC results. C) Heatmap displaying HER2 IHC false‐positivity in IHC 3+/drPCR‐negative cases (*n* = 22) confirmed by multiple methods. pos, positive; neg, negative; equi, equivocal. *, cases with low tumor purity (≤25%). **, discordance between HER2 IHC and drPCR results due to low tumor purity. D) Schematic diagram of the re‐evaluation of HER2 IHC results using an AI‐based HER2 IHC scoring algorithm in the SNUH cohort (*n* = 200). E,F) Comparison of HER2 IHC results between manual assessment and AI‐based automated scoring in the SNUH cohort (*n* = 200). The graph (left) and table (right) show changes in HER2 IHC status based on the evaluation methods. Data are presented as patient distribution (%) of case numbers.

While HER2 drPCR showed high agreement with conventional HER2 testing methods in the final evaluation, some discordant cases were revised to concordance upon IHC and/or ISH re‐evaluation, primarily due to the frequent occurrence of IHC false positives. Among patients with an original HER2 IHC score 3+ (*n* = 94), 22 cases (23.4%) were classified as HER2‐negative by the drPCR assay. For these discrepant cases, we re‐evaluated the HER2 IHC data using both manual assessment and an AI‐based automated scoring system. When the original HER2 IHC 3+ score was revised to 2+ (equivocal) or 0/1+ (negative), ISH was performed to verify the true HER2 status (**Figure** **4**A). Interestingly, all these cases, except three discrepant cases with low tumor purity, were identified as HER2 true‐negative with IHC‐equivocal/ISH‐negative results upon re‐evaluation (Figure 4B,C; Table S7, Supporting Information), ensuring the higher accuracy of drPCR than IHC.

Notably, when evaluating the original HER2 IHC data with an AI‐based automatic scoring algorithm in a representative cohort (SNUH, *n* = 200), approximately half of the original HER2 IHC 3+ cases (42.1%) were downgraded to IHC 2+, and 53.6% of the original IHC‐equivocal (2+) cases were estimated as HER2‐negative (1+ or 0) (Figure 4D–F). These findings highlight the potential risks of current HER2 testing, which considerably relies on the subjective interpretation of HER2 IHC as an initial test, suggesting that drPCR could be valuable in correcting false‐positive IHC results.

### 2.8 Impact of Tumor Purity and Intratumoral Heterogeneity on drPCR‐Based HER2 Assessment

As low tumor purity and high HER2 intratumoral heterogeneity (ITH) have been considered the main causes of the discrepancy between ddPCR and standard HER2 testing methods,^[^ ^27^, ^28^, ^29^ ^]^ their effect on drPCR‐based HER2 assessment was investigated. Among 363 patients, 56 (15.4%) had low tumor purity (≤25%), resulting in relatively lower diagnostic accuracy for HER2 drPCR compared to that of the group with tumor purity > 25% (Table S8, Supporting Information). While specificity and PPV were unaffected by tumor purity, sensitivity was remarkably decreased in the low‐purity group. All discordant cases (*n* = 7) in the SCHH and CNUH cohorts had low tumor content (≤25%), with four cases showing improved results after tumor macrodissection (Figure S7A and Table S9, Supporting Information). Among the three remaining discrepant cases, one case was found to have no residual tumor due to tissue loss during multiple sectioning after the IHC/ISH test (Case 1), while the other two exhibited high levels of tumor‐infiltrating lymphocytes (case 2; Figure S7B, Supporting Information). Additionally, macrodissection did not affect drPCR results for samples with tumor purity >25% (Table S9, Supporting Information).

To simplify correction of drPCR false negatives caused by low tumor purity without inconvenient macrodissection, an optimal drPCR cutoff of 1.385 was determined for cases with tumor purity ≤ 25% using ROC curve analysis (AUC = 0.942; Figure S7C, Supporting Information). Applying this alternative cutoff to drPCR false‐negative cases with low tumor purity (*n* = 7) reclassified five cases as HER2‐positive (Figure S7D, Supporting Information), indicating the effective correction. Collectively, these findings suggest a potential guideline for drPCR‐based HER2 assessment for cases with tumor purity ≤25%, which could either adopt macrodissection or apply the alternative cutoff for low tumor purity (Figure S7E, Supporting Information).

While low tumor purity affected drPCR results in discordant cases in the SCHH and CNUH cohorts, HER2 ITH was a major cause of discrepancy between drPCR and IHC/ISH results in the SNUH cohort, which included a high proportion of HER2 IHC‐equivocal cases. Among eight discordant cases in the SNUH cohort, seven exhibited high HER2 ITH as confirmed by AI‐based algorithm (Figure S8A,B, Supporting Information). Given the conflicting interpretation of IHC/ISH in these discordant cases (Figure 3G), these data support the notion that HER2 ITH may cause intra‐ and inter‐observer variability in the IHC/ISH assays. Since the re‐evaluation confirmed the high accuracy of drPCR, drPCR may provide more reliable data on HER2 status in cases with high HER2 ITH, as it reflects the overall HER2 status across a larger tumor area compared to conventional IHC/ISH, which assesses only limited tumor regions.

### 2.9 Alternative Reference Controls for Detection of CEP17 Abnormalities

**Figure 5:**
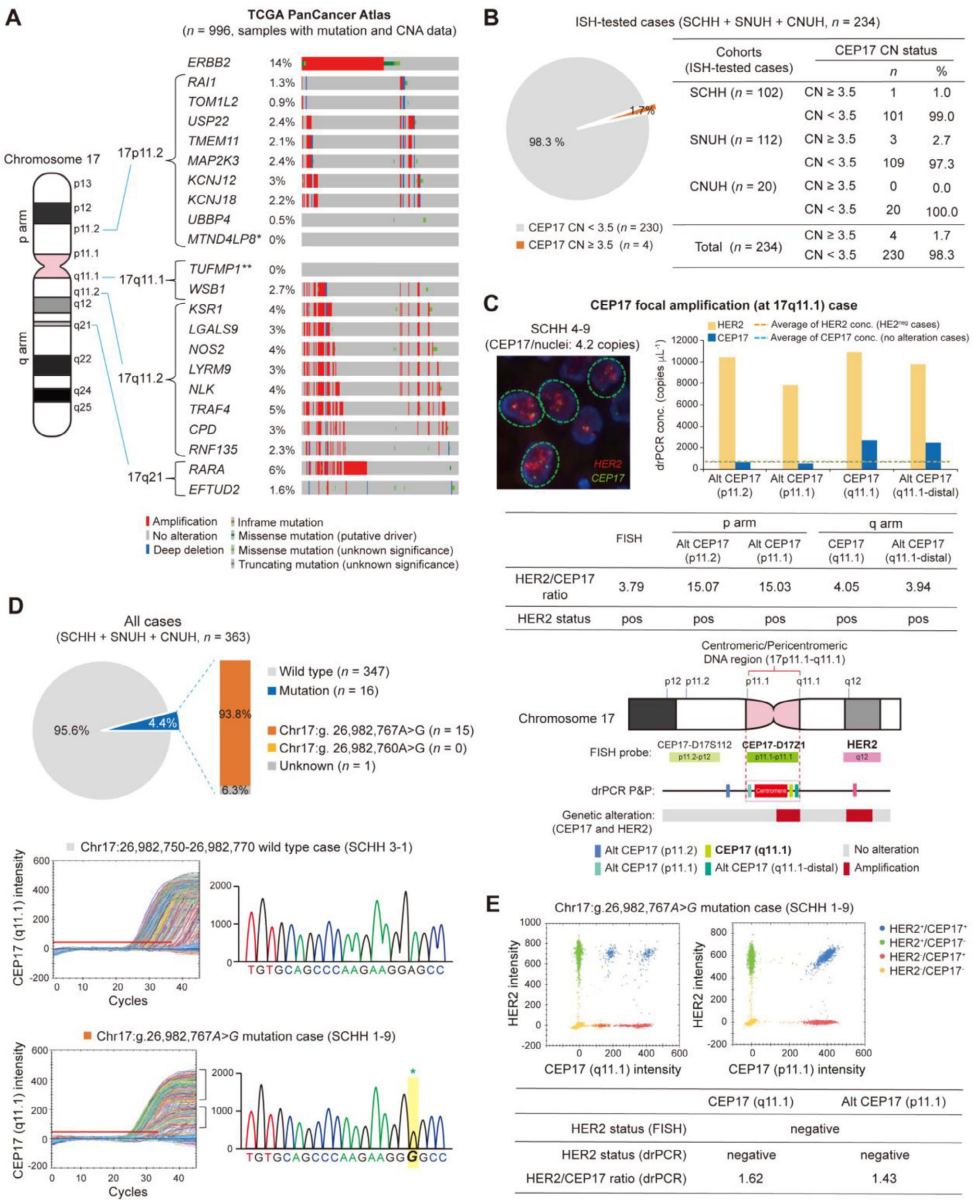
Alternative CEP17 reference controls for detecting CEP17 abnormalities. A) Genetic alterations in genes/pseudogenes located in the indicated regions of chromosome 17 in patients with breast cancer from the TCGA PanCancer Atlas (*n* = 996). * and **, alternative reference P&P regions encoding the pseudogenes (Alt CEP17p11.2 and CEP17q11.1‐distal, respectively). B) Frequency of CEP17 CN gain in ISH‐tested patients from the indicated cohorts (total *n* = 234; SCHH, *n* = 102; SNUH, *n* = 112; CNUH, *n* = 20). An average CEP17 DNA copy number (CN) ≥ 3.5 was considered a CN gain. C) Representative case with CEP17 focal amplification on the 17q11.1 region, confirmed by FISH and drPCR analysis. D) Proportion of cases harboring point mutations within the CEP17q11.1 probe region in the breast cancer cohort (*n* = 363), confirmed by drPCR and Sanger sequencing (upper). Results from two representative cases (wild type vs mutant type) are shown (lower). One case presumed to have a CEP17 mutation could not be assessed by Sanger sequencing due to severe DNA degradation from the FFPE sample (Unknown, *n* = 1). E) Representative case harboring a CEP17 point mutation confirmed by drPCR using the alternative reference P&P (Alt CEP17p11.1).

In standard HER2 ISH testing, polysomy 17 or focal CN gain within the CEP17 probe region, observed in some patients, is a major complicating factor that can lead to HER2 status underestimation.^[^ ^40^, ^41^, ^42^ ^]^ Consistently, analysis of the TCGA dataset showed that CN gain of genes near the pericentromeric region of chromosome 17 (17p11.2–q11.1) occurs in ≈0.5–5% of patients with breast cancer (**Figure** **5**A). Notably, no mutations were found in the *MTND4LP8* (17p11.2) and *TUFMP1* (17q11.1) pseudogenes targeted by our alternative CEP17 P&Ps, whereas other genes previously proposed as alternative reference genes for HER2 CNA assays, including *RAI1* (17p11.2), *RARA* (17q21), *EFTUD2* (17q21), *EIF2C1* (1p34), and *TFF3* (21q22),^[^ ^26^, ^30^, ^31^, ^32^, ^41^ ^]^ exhibited genetic mutations in ≈1–6% of patients (Figure 5A; Figure S9, Supporting Information).

We also evaluated CEP17 CNA in our cohorts using ISH and drPCR analyses. Among ISH‐tested patients (*n* = 234), four (1.7%) had CEP17 CN gain (Figure 5B), which was also confirmed by drPCR (Figure 5C; Figure S10A–C, Supporting Information). Remarkably, unlike the standard CEP17 ISH probe (D17Z1) that cannot distinguish between focal CN and polysomy 17 due to its broad binding across both arms of the pericentromeric regions (17p11.1–17q11.1), our reference P&Ps targeting four different focal loci on either the p or q arm of chromosome 17 pericentromere detected focal CEP17 gain on the q arm in three cases (Figure 5C; Figure S10A,B, Supporting Information) and potential polysomy 17 in one case (Figure S10C, Supporting Information). These results suggest that a more accurate assessment of CEP17 CN status to avoid HER2 misinterpretation may be achieved using drPCR rather than ISH.

Unexpectedly, we discovered a point mutation within the CEP17q11.1 probe region in some patients with breast cancer (16/363, 4.4%) and cell lines (Figure 5D; Figure S11A,B, Supporting Information). The mutation only partially inhibited the CEP17q11.1 probe fluorescence intensity without affecting the quantification of CEP17 CN, and substitution of the mutated nucleotide with the wild type restored the probe fluorescence intensity in the drPCR (Figure 5D,E; Figure S11B–D, Supporting Information). Using our P&Ps, we found that SKBR3 and HCC‐1954 cell lines have complex patterns of CEP17 abnormalities, including point mutation, focal CN loss (p arm), and CN gain (q arm) (Figure S11E, Supporting Information). Collectively, these data suggest that our drPCR assay may enable more accurate estimation of HER2 and CEP17 CNAs even in the presence of diverse CEP17 abnormalities.

### 2.10 Association of *ERBB2* CN Loss with HER2‐zero in Patients with Breast Cancer

**Figure 6:**
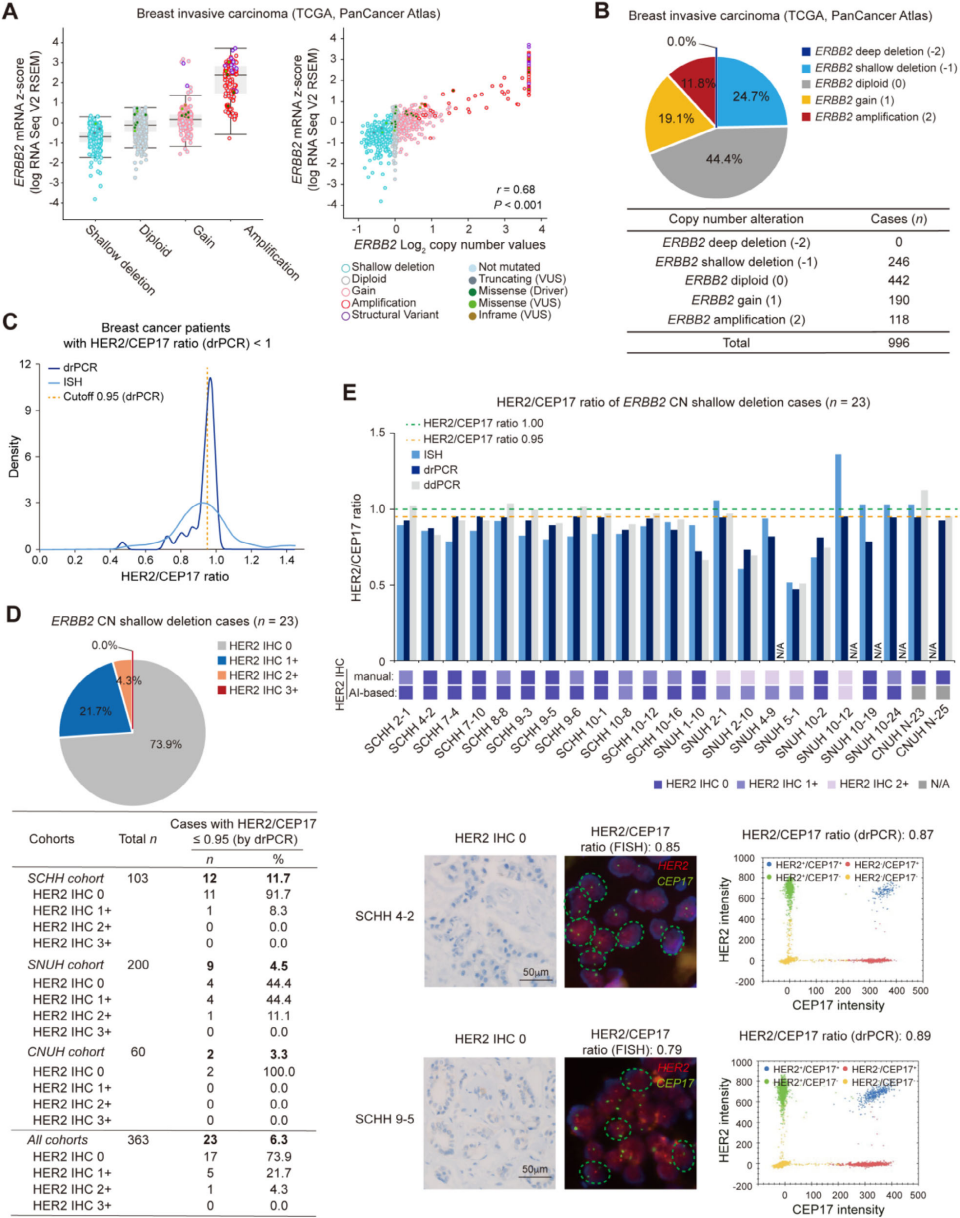
Shallow deletion of *ERBB2* CN in patients with breast cancer. A) Association between *ERBB2* expression and genetic alterations in patients with breast cancer from the TCGA PanCancer Atlas (*n* = 996), analyzed using Spearman's correlation. VUS, variants of unknown significance. *ERBB2* shallow deletion, *n* = 246; diploid, *n* = 442; gain, *n* = 190; amplification, *n* = 118. B) Proportion of cases with the indicated *ERBB2* CN status in the TCGA breast cancer dataset (*n* = 996). C) The kernel density estimation (KDE) plot displaying the distribution of HER2/CEP17 ratios measured by drPCR and ISH in patients with breast cancer with a HER2/CEP17 < 1 as determined by drPCR (*n* = 49). ISH was not performed in 7 of these 49 cases. D) Proportion of cases with shallow *ERBB2* CN deletion (HER2/CEP17 ratio ≤0.95) as determined by drPCR in the indicated cohorts (*n* = 23). HER2 IHC status in these cases was reviewed using an AI‐based automatic scoring system to compare drPCR and IHC results. (E) Comparison of HER2/CEP17 ratios measured by drPCR, ddPCR, and ISH in patients with breast cancer with shallow *ERBB2* CN deletion (upper, *n* = 23). Manual and AI‐based HER2 IHC scores are also shown. N/A, not applicable. Representative images of IHC/FISH (lower left) and drPCR (lower right) results are shown for cases with *ERBB2* CN loss. In FISH image, nuclei with shallow deletion of *ERBB2* CN are circled with dashed green line.

Although diagnosing HER2‐low/ultra‐low tumors has become crucial for identifying patients eligible for the recently approved T‐DXd therapy, defining HER2‐zero and HER2‐low/ultra‐low remains challenging due to significant variability in the pathologists interpretation of IHC 0 versus 1+.^[^ ^3^, ^4^, ^5^ ^]^ A recent study demonstrated that *ERBB2* hemizygous deletion, found in ≈7% of patients with breast cancer, is associated with resistance to T‐DXd.^[^ ^43^ ^]^ Consistently, a more recent report indicated a subset of patients with HER2‐negative breast cancer harboring heterozygous loss of *ERBB2* and minimal HER2 expression, characterized by distinct clinical features that may contribute to T‐DXd resistance.^[^ ^44^ ^]^ To explore the potential clinical applicability of drPCR assay for identifying T‐DXd‐resistant patients with *ERBB2* CN deletion, we examined the prevalence of *ERBB2* CN deletion in patients with breast cancer. In the TCGA dataset, none of these patients had *ERBB2* homozygous deletion, while ≈25% of the patients showed shallow/hemizygous deletion of *ERBB2*, with decreased *ERBB2* mRNA levels (**Figure** **6**A,B). We further investigated our cohorts to define patients with *ERBB2* shallow deletion based on drPCR and ISH results. As the HER2 CNA status is assessed relative to a reference control (HER2/reference = 1, diploid; HER2/reference >1, potential HER2 CN gain; HER2/reference <1, potential HER2 CN loss) in ISH and dPCR assays, we first examined the proportion of cases with a HER2/CEP17 ratio <1 by drPCR. Of the 363 patients, 49 were identified as having HER2/CEP17 ratio <1 by drPCR. Among these, ISH results were available for 42 cases, of which 30 also showed a HER2/CEP17 ratio < 1 by ISH. Comparison of distribution of drPCR and ISH ratios in cases with a HER2/CEP17 ratio <1 using the kernel density estimation (KDE) plot showed that drPCR measurement exhibited a sharp peak near 1.0, whereas ISH results showed a broader, left‐shifted distribution (Figure 6C). By considering these distribution patterns, as well as potential technical variability of drPCR (estimated ranges: 0.01–0.05, based on CV%), we conservatively set the cutoff for shallow *ERBB2* deletion at 0.95. When applying this cutoff, drPCR analysis identified 23 patients (6.3%; 23/363) with potential shallow *ERBB2* deletion (HER2/CEP17 ≤ 0.95), which were also confirmed by ISH and ddPCR (Figure 6D,E; Table S10, Supporting Information). Interestingly, AI‐based re‐evaluation of HER2 IHC scores indicated that 73.9% of these cases were classified as HER2‐zero (score 0), whereas manual assessment of IHC showed inconsistent scores. Overall, these findings suggest that drPCR‐based assessment of *ERBB2* shallow deletion combined with AI‐assisted IHC analysis may be valuable for identifying true HER2‐zero patients with potential resistance to T‐DXd therapy.

### 2.11 Predictive Utility of drPCR‐based HER2 Assessment for Anti‐HER2 Therapy Response

**Figure 7:**
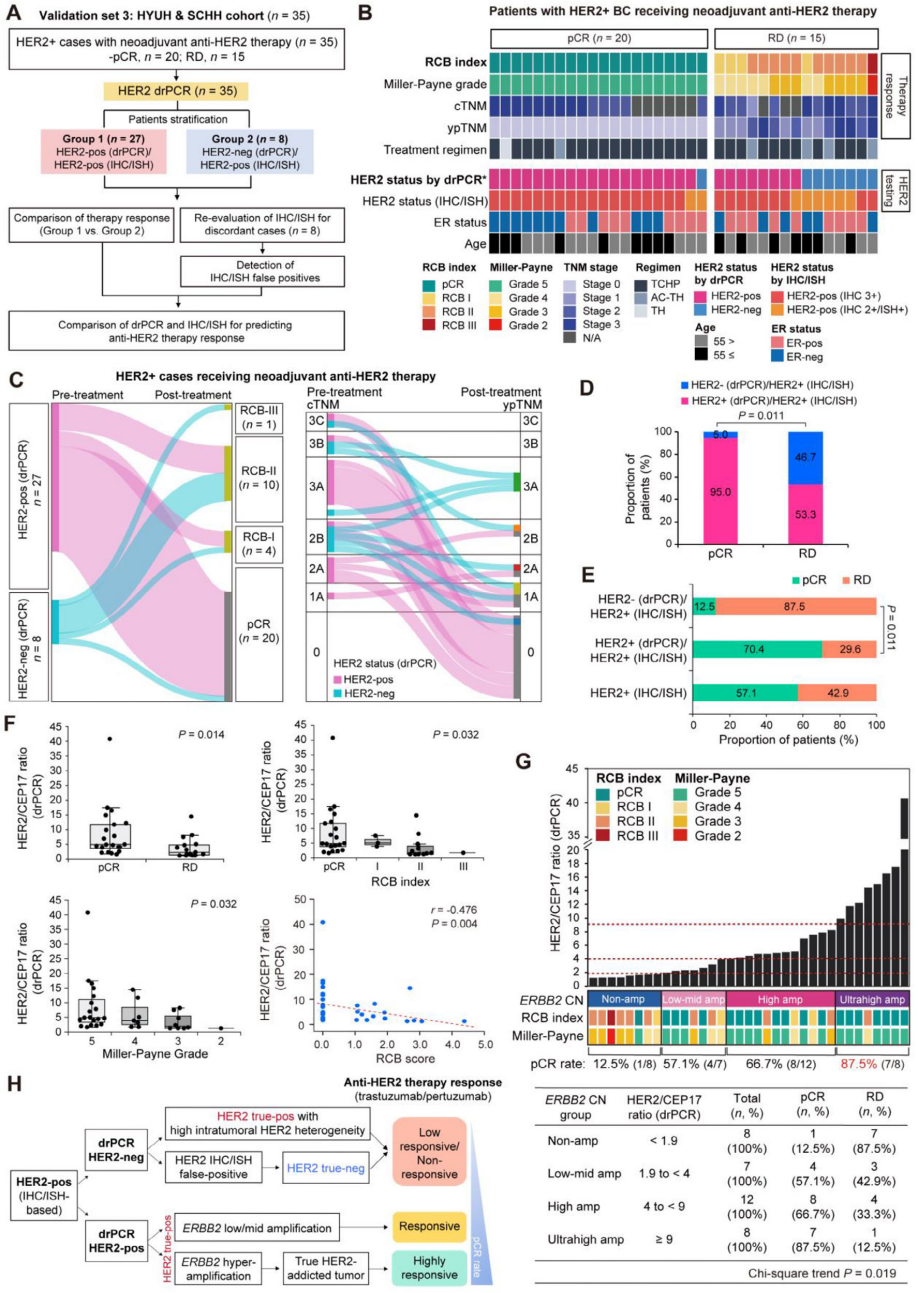
drPCR‐based HER2 status assessment in biopsy specimens from HER2‐positive patients receiving neoadjuvant anti‐HER2 therapy. A) Workflow for evaluating the utility of drPCR in predicting response to neoadjuvant anti‐HER therapy in 35 HER2‐positive patients with breast cancer. B) Heatmap depicting treatment response, clinicopathological features, and HER2 status (by both IHC/ISH and drPCR) for each patient initially diagnosed as HER2‐positive followed by neoadjuvant therapy (total *n* = 35; pCR, *n* = 20; RD, *n* = 15). *, statistically significant in the multivariable regression analysis (adjusted for age, ER status, HER2 IHC score, and treatment regimen). C) Sankey diagram showing treatment response based on the Residual Cancer Burden (RCB) index (left) and changes in TNM stage (right) between HER2 drPCR‐positive (*n* = 27) and drPCR‐negative (*n* = 8) groups. Of the 27 HER2 drPCR‐positive cases, nine lacking baseline cTNM information were excluded from the cTNM‐to‐ypTNM Sankey diagram. Diagram generated using SankeyMATIC (https://sankeymatic.com). D) Comparison of the proportions of HER2 drPCR‐positive (*n* = 27) and drPCR‐negative (*n* = 8) cases within the pathological complete response (pCR) and residual disease (RD) groups. E) Overall rates of pCR and RD in the indicated groups. *p*‐values by Fisher's exact test D,E). F) Box plots showing distribution of HER2/CEP17 ratios measured by drPCR, stratified by pCR vs RD (upper left; pCR, *n* = 20; RD, *n* = 15), RCB indexes (upper right; pCR, *n* = 20; RCB I, *n* = 3; RCB II, *n* = 11; RCB III, *n* = 1), and Miller‐Payne grades (lower left; score 5, *n* = 20; score 4, *n* = 7, score 3, *n* = 7, score 2, *n* = 1). *p*‐values were calculated using Mann‐Whitney U test (for pCR vs RD) or Kruskal‐Wallis (for RCB indexes and Miller‐Payne grades) test. Scatter plot showing the correlation between HER2/CEP17 ratio (drPCR) and RCB scores in patients receiving neoadjuvant anti‐HER2 therapy, analyzed using Spearmans’ rank correlation (lower right). G) Association of *ERBB2* CN levels with neoadjuvant therapy response, based on HER/CEP17 ratios measured by drPCR. *P*‐values by Chi‐square test for trend. H) Schematic diagram illustrating the drPCR‐based prediction of anti‐HER2 therapy response.

Given that HER2 serves as a companion diagnostic marker for anti‐HER2 therapy, we investigated whether drPCR‐based HER2 assessment is clinically available to predict response to anti‐HER2 therapy. Thirty‐five patients with breast cancer initially diagnosed as HER2‐positive by IHC/ISH‐based HER2 testing followed by neoadjuvant anti‐HER2 therapy (trastuzumab/pertuzumab) plus chemotherapy were enrolled (**Figure** **7**A). According to clinical records, 20 patients (57.1%) achieved pathological complete response (pCR), while 15 (42.9%) had residual disease (RD). In biopsy specimens, HER2 status was re‐assessed by drPCR, categorizing patients into concordant (drPCR^Pos^/IHC‐ISH^Pos^) and discordant (drPCR^Neg^/IHC‐ISH^Pos^) groups, and treatment outcomes were compared based on residual cancer burden (RCB) index, Miller‐Payne grade, and ypTNM stage. For discordant cases, IHC/ISH results were re‐evaluated by cross‐validation with independent pathologists to identify the reasons for discrepancies between drPCR and IHC/ISH testing (Figure 7A; Figure S12A, Supporting Information).

When HER2 status was re‐assessed by drPCR, 8 of 35 cases (22.9%) were classified as HER2‐negative, contrary to the initial IHC/ISH results. Of note, 7 of 8 (87.5%) of the discordant cases (drPCR^neg^/IHC‐ISH^pos^) had a partial or poor response to anti‐HER2 therapy, whereas a high rate of pCR was achieved in the concordant group (Figure 7B,C). Among patients who achieved pCR, 95% were HER2 drPCR‐positive, whereas 46.7% of RD cases were HER2 drPCR‐negative (Figure 7D). The overall pCR rate in patients confirmed as HER2‐positive by both drPCR and IHC/ISH reached 70.4% (Figure 7E). To elucidate whether HER2 status by drPCR independently predicts treatment response, we conducted a multivariable logistic regression analysis incorporating age, ER status, HER2 IHC score, and treatment regimen as covariates. Among these variables, only HER2 status by drPCR remained a significant predictor of treatment response (odds ratio [OR] = 19.56, 95% confidence interval [CI] = 1.01─377.88; *p* = 0.049), whereas other covariates were not statistically significant (Figure 7B; Table S11, Supporting Information). These findings demonstrated that drPCR predicted anti‐HER2 therapy response more accurately than IHC/ISH, suggesting the possibility of false positives in initial IHC/ISH‐based diagnoses. Indeed, re‐evaluation of eight discordant cases revealed that four were IHC/ISH false positives, and one had high HER2 ITH with only 5% HER2‐positivity; all exhibited poor therapeutic outcomes (Figure S12A–D, Supporting Information). Two additional cases showed ambiguous ISH results due to intra‐ and inter‐observer variability (Figure S12A–C, Supporting Information).

A significantly higher HER2/CEP17 ratio by drPCR was observed in patients who achieved pCR compared to those with RD (*p* = 0.014; Figure 7F). Likewise, this ratio gradually decreased across RCB index groups from pCR to RCB‐III (*p* = 0.032) and Miller‐Payne grades from 5 to 2 (*p* = 0.032). Furthermore, a negative correlation was shown between drPCR ratios and RCB scores (*r* = ‐0.476, *p* = 0.004). Notably, patients harboring ultrahigh *ERBB2* amplification (HER2/CEP17 ratio ≥9) achieved the highest pCR rate (87.5%, 7/8) compared to that of other HER2‐positive cases with lower *ERBB2* amplification levels (Figure 7G; Figure S12E, Supporting Information), implying that higher levels of *ERBB2* CNs as measured by drPCR may serve as a reliable predictor of better treatment response.

Collectively, these results suggest that HER2‐negativity by drPCR indicates a poorer response to neoadjuvant anti‐HER2 therapy, reflecting high HER2 ITH or IHC/ISH false positives. In contrast, HER2‐positivity by drPCR can predict a favorable response to therapy. The drPCR assay also identified highly HER2‐addicted patients with *ERBB2* hyperamplification who responded better to anti‐HER2 therapy (Figure 7H). Therefore, drPCR‐based HER2 status assessment may be valuable to precisely discriminate responders to anti‐HER2 therapy.

## 3 Discussion

**Figure 8:**
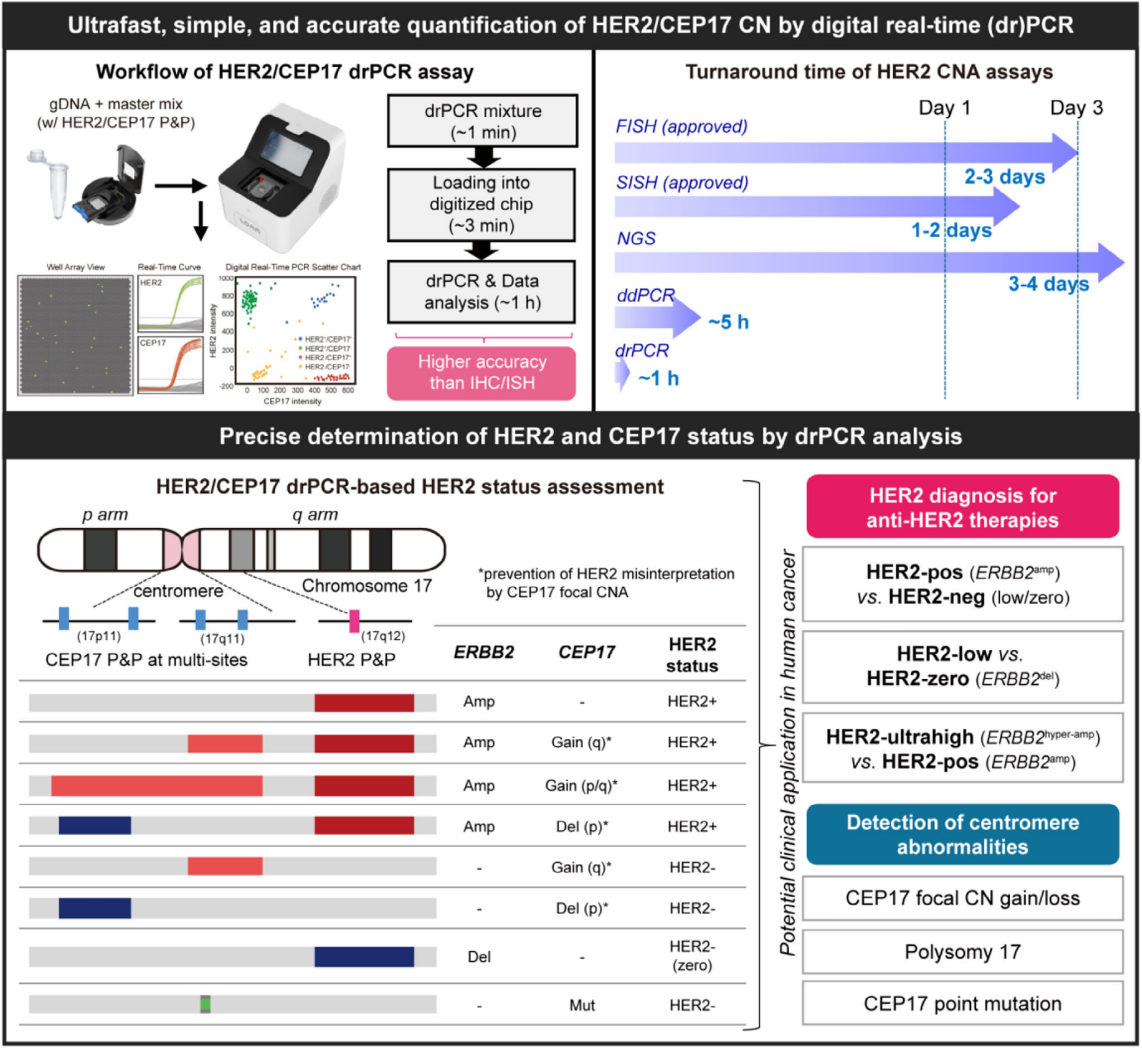
Advanced features and proposed clinical application of the HER2/CEP17 drPCR assay. Schematic illustration of the superior advantages of the drPCR assay in assessing HER2 status, offering faster, easier, and more accurate measurement of HER2 CNA compared to conventional HER2 testing methods (Upper). A proposed model for precise determination of HER2 status with HER2, CEP17, and alternative CEP17 primer‐probe sets (P&Ps) in the drPCR assay. The multi‐site reference P&Ps can distinguish focal CEP17 CN gain occurring on either arm, thereby preventing misinterpretation of HER2 status (lower left). The HER2/CEP17 drPCR assay may enable precise diagnosis of HER2‐altered tumors, including determination of HER2‐positive versus HER2‐neg, HER2‐low versus HER2‐zero, and HER2‐ultrahigh within the HER2‐positive group, to improve predictions of anti‐HER2 therapy response. Additionally, the drPCR assay may be valuable for detecting focal CN gain/loss, as well as a point mutation in centromeric/pericentromeric regions of chromosome 17 (lower right).

Our study proposes an improved HER2 testing method using drPCR, which significantly reduce diagnostic time and labor, while enhancing accuracy and sensitivity. Although HER2 has recently been highlighted as a potential tumor‐agnostic biomarker across all solid tumors,^[^ ^3^, ^4^, ^5^ ^]^ current standard HER2 testing methods still have significant limitations.^[^ ^2^, ^8^, ^9^, ^10^ ^]^ Despite continuous updates to the ASCO/CAP guidelines to improve HER2 testing accuracy,^[^ ^6^, ^40^ ^]^ the revised algorithm, still based on IHC/ISH, has shown limited improvement in interpreting HER2 status.^[^ ^45^, ^46^, ^47^ ^]^ Compared to conventional assays, our drPCR method offers superior advantages for assessing HER2 status. drPCR provided more precise HER2 CNA measurement with higher sensitivity than ISH, NGS, and ddPCR, while also greatly shortening and simplifying the analysis process (Figure 8, upper). drPCR also effectively detected false‐positive IHC results, indicating higher accuracy of drPCR than IHC. Moreover, our new strategy for reference control targeting multi‐sites within the CEP17 probe region allows for easy detection of focal CEP17 CNA, thereby preventing HER2 status misinterpretation (Figure 8, lower). Furthermore, we established a standardized drPCR method through multicenter cross‐validation, ensuring its clinical validity, whereas previous NGS and ddPCR studies showed inconsistent results due to highly variable cutoffs and reference controls across institutions and platforms. Given the advantages of drPCR over conventional HER2 testing assays, drPCR‐based HER2 assessment may be feasible for routine clinical practice and could improve the limitations of standard HER2 testing.

Notably, our established drPCR‐based HER2 testing can better predict response to anti‐HER2 therapy than conventional HER2 IHC/ISH. Although anti‐HER2 therapies are well‐established for human cancers, a substantial number of HER2‐positive patients exhibit partial response or resistance to treatment, which remains a significant challenge.^[^ ^1^, ^2^ ^]^ In addition to various molecular mechanisms underlying innate and acquired resistance to anti‐HER2 therapies, high HER2 ITH has emerged as a main cause of resistance to HER2‐targeted therapies,^[^ ^48^, ^49^, ^50^ ^]^ emphasizing the need for patient stratification according to HER2 ITH for better prediction of response to anti‐HER2 therapies. Furthermore, inaccurate decision of HER2 status during diagnosis may lead to an inefficient treatment response. Interestingly, in a neoadjuvant setting of anti‐HER2 therapies, our findings showed that HER2 drPCR‐negativity reflects a poor response to treatment, which may be attributed to false‐positive IHC/ISH results or high HER2 ITH, both associated with a lack of pCR (Figure 7; Figure S12, Supporting Information). Meanwhile, HER2 drPCR‐positive cases showed a higher pCR rate than HER2 IHC/ISH‐positive groups. Collectively, these results suggest that drPCR offers improved accuracy in evaluating HER2 status and predicting responders to anti‐HER2 therapies compared to conventional HER2 testing methods. For drPCR‐negative cases with HER2 ITH showing a poor response to trastuzumab/pertuzumab, T‐DXd may be a better option to improve outcomes, as it is effective in tumors with HER2 ITH due to its bystander effect on adjacent tumor cells.^[^ ^2^, ^3^, ^43^ ^]^ While the current study demonstrates the promising clinical utility of the drPCR assay in predicting therapeutic response to neoadjuvant anti‐HER2 therapy, the analysis was conducted in a relatively small cohort of 35 HER2‐positive patients. This limited sample size may restrict statistical power and limit the generalizability of the findings. Although multivariable analysis identified HER2 status by drPCR as a statistically significant independent predictor of pCR in this small cohort (Table S11, Supporting Information), further validation in larger, independent cohorts is warranted to confirm the robustness of this finding. In particular, prospective clinical studies will be essential to more accurately evaluate the predictive performance of drPCR and its potential superiority over current diagnostic methods.

In addition to assessing HER2‐positive versus HER2‐negative status based on *ERBB2* amplification, the HER2 drPCR assay may be suitable for detecting hemizygous deletions and hyperamplification of *ERBB2*, which could be associated with responses to T‐DXd and trastuzumab monotherapy, respectively. Although T‐DXd has recently been approved for HER2‐low/ultra‐low breast cancers, distinguishing HER2‐zero and HER2‐low groups using IHC remains challenging due to high inter‐observer variability.^[^ ^3^, ^4^, ^17^ ^]^ Interestingly, a recent study investigating clinical biomarkers related to T‐DXd resistance revealed that patients with *ERBB2* hemizygous deletion did not respond to T‐DXd.^[^ ^43^ ^]^ Similarly, another recent study support the clinical significance of *ERBB2* hemizygous deletion in patients with HER2‐negative breast cancer, which was associated with minimal *ERBB2* mRNA levels and poor clinical outcomes, suggesting its potential as a predictive marker for T‐DXd resistance.^[^ ^44^ ^]^ Consistently, our HER2 drPCR analysis, combined with HER2 IHC re‐evaluation using an AI‐based automatic system, showed that most patients harboring shallow *ERBB2* CN loss (HER2/CEP17 ratio ≤ 0.95) displayed HER2 IHC 0 in the AI‐based scoring algorithm. While further studies are needed to optimize the cutoff for defining *ERBB2* CN loss in the dPCR and ISH assays and to clarify its relationship with T‐DXd response in larger cohorts, this finding suggests that drPCR‐based HER2 assessment for HER2 IHC‐negative patients may be helpful in distinguishing HER2‐zero from HER2‐low/ultra‐low groups by detecting *ERBB2* hemizygous deletion for better prediction of T‐DXd response. Furthermore, increasing evidence has suggested that hyperamplification of *ERBB2*, with a HER/CEP17 ratio greater than the ISH cutoff 2.0, represents a true HER2‐addicted tumor phenotype that is highly responsive to trastuzumab monotherapy or T‐DXd therapy in HER2‐positive breast and gastric cancers.^[^ ^1^, ^51^ ^]^ Consistently, our data showed that patients with ultrahigh *ERBB2* amplification (HER2/CEP17 ratio ≥9) achieved a high rate of pCR (Figure 7G; Figure S12, Supporting Information). Therefore, determining *ERBB2* amplification levels within the HER2‐positive group using drPCR may be useful for identifying patients most likely to benefit from anti‐HER2 therapies, potentially allowing for reduced or omitted chemotherapy. Given the growing importance of precise measurement of various types of HER2 CNA for clinical decision‐making, the HER2 drPCR assay, which provides a simpler and more practical approach to assessing *ERBB2* CN status compared to ISH, could be broadly used to determine HER2 CNA, including hemizygous deletion, amplification, and hyperamplification, for appropriate selection of patients who will respond to different treatment strategies of anti‐HER2 therapies (Figure 8).

Our study highlights the technical advantages and clinical applicability of the LOAA drPCR platform over conventional dPCR systems. Recently, dPCR has increasingly emerged as a powerful molecular diagnostic tool, offering high precision and sensitivity through absolute quantification without the need for standard curves.^[^ ^52^, ^53^ ^]^ Despite these benefits, the routine clinical adoption of many commercial dPCR platforms has been limited by several factors, including complex workflow due to the lack of full system integration, variability and contamination risks associated with manual partitioning, moderate throughput time, restricted dynamic range, and high cost.^[^ ^35^ ^]^ In particular, their reliance on end‐point fluorescence detection is a common drawback that may increase the risk of false‐positive results. To address these challenges, there is a growing need for faster, simpler, and fully automated real‐time dPCR platforms that are suitable for routine clinical diagnostics. The BioMark HD system (Fluidigm) was the first to incorporate real‐time monitoring into dPCR, but it is limited by a low partition number (<800), long assay times (≈4 h), lack of integration requiring a separate controller, and higher cost compared to other platforms. To overcome these limitations, Zhou et al. developed an inexpensive, fully integrated real‐time dPCR system using a PDMS chip fabricated via soft lithography.^[^ ^38^ ^]^ However, its partition number (≈1120 wells) and throughput speed remain modest. More recently, Yao et al. introduced a MEMS‐based real‐time dPCR platform with ≈20000 partitions per sample, similar to the LOAA system, but their system requires an external chip loader.^[^ ^37^ ^]^ In addition, while the BioMark HD and Yao's platform rely on a microfluidic chip loader to dispense reagents into the wells, the LOAA and Zhou's devices utilize a self‐priming mechanism in which the reagents are automatically distributed into the partitions. Notably, the fully integrated, automated LOAA drPCR enables a simpler and faster assay with shorter throughput time (≈1 h), and also supports HDR across eight orders of magnitude, which is the widest range reported among current dPCR platforms. Furthermore, the LOAA drPCR platform offers both a portable On‐point model, which is suitable for point‐of‐care testing, and a multi‐sample model designed for high‐throughput analysis. In addition, the simplified reagent composition and compact device make the LOAA platform more cost‐effective than other dPCR systems. Therefore, the LOAA drPCR's simple workflow, robust performance, and complete automation support its broad clinical applicability.

## 4 Conclusion

Our data demonstrate the clinical reliability of drPCR‐based HER2 assessment, offering great advantages such as high accuracy, reproducibility, and ultrafast and simple processes compared to conventional HER2 testing methods. Given these benefits, we propose a new HER2 testing algorithm incorporating drPCR to enhance the accuracy and efficiency of current HER2 diagnostics. The HER2 drPCR assay could serve as an alternative to ISH, offering much easier and accurate *ERBB2* CN measurement (Figures S13A,B, Supporting Information). Furthermore, considering the frequency of false‐positive IHC results, concomitant evaluation of HER2 status with both IHC and drPCR for all cases could significantly improve the diagnostic accuracy while reducing turnaround time compared to conventional methods (Figures S13B,S14, Supporting Information). While further large‐scale clinical validation is required, HER2 drPCR could potentially serve as a one‐step HER2 test, replacing IHC and ISH as complementary methods for drPCR (Figure S13C, Supporting Information). Although only a minimal number of cases fell into the gray zone in our study (HER2/CEP17 ratio 1.8 to <1.9), the drPCR‐equivocal cases should be carefully managed by repeat drPCR testing and re‐evaluation of IHC/ISH results. Establishing standardized clinical guidelines for managing these gray zone cases should be addressed in future prospective studies. Collectively, implementing drPCR assay as a new HER2 testing method is expected to enable more accurate, precise, and rapid determination of HER2 status for better prediction of response to anti‐HER2 therapy.

## 5 Experimental Section

### Patient Cohorts and Ethical Approval

To develop and establish a new standardized drPCR‐based method for HER2 status assessment through a multicenter, retrospective study, a total of 363 patients with breast cancer from three independent institutions in the Republic of Korea were enrolled (Training set, SCHH cohort, *n* = 103; Validation set 1, SNUH cohort, *n* = 200; and Validation set 2, CNUH cohort, *n* = 60). To evaluate the predictive value of drPCR for response to anti‐HER2 therapy, 35 patients (SCHH, *n* = 9; HYUH, *n* = 26) initially diagnosed as HER2‐positive followed by neoadjuvant anti‐HER2 therapy plus chemotherapy were enrolled. Detailed information for patient cohort is described in the Supplementary Methods.

The study protocol was reviewed and approved by the Institutional Review Boards of SCHH (2022‐02‐018), SNUH (H‐2203‐058‐1305), CNUH (2022‐02‐018), and HYUH (2024‐11‐041). Informed consent was waived as the study was retrospective in design and utilized anonymized clinical data. This study was conducted in accordance with the Declaration of Helsinki recommendations for biomedical research involving human participants.

### IHC

Standard IHC procedures were performed on FFPE tissues using an anti‐HER2/neu antibody (pre‐diluted, 4B5 clone, 790–4493, Ventana Medical Systems; Roche Diagnostics, Indianapolis, IN, USA) and the Ventana BenchMark XT Staining System. IHC results were interpreted by experienced breast pathologists and graded from 0 to 3+ based on the intensity and pattern of membrane staining. A complete, intense circumferential membranous staining on ≥ 10% of tumor cells (score 3+) was considered HER2 IHC‐positive, following the 2018 ASCO/CAP guidelines.^[^ ^6^ ^]^ For AI‐based HER2 IHC quantification, slides were scanned on an Aperio ScanScope CS (v12.4.6.5003, Leica Biosystems, Wetzlar, Germany). HER2 IHC scores were then calculated using the Image Scope computerized image analysis system with the “IHC HER2 Breast Dako HercepTest v1” algorithm.^[^ ^54^, ^55^, ^56^, ^57^ ^]^

### FISH

FISH was performed using the PathVysion DNA Probe Kit (HER2/neu probe, orange; CEP17 probe, green; Abbott Molecular, Abbott Park, IL, USA) following the manufacturer's instructions. Representative slides were marked with a 0.5 × 0.5 cm target area, and 2 µm thick tissue sections were prepared from archival paraffin blocks. Unstained slides were deparaffinized, pretreated at 80 °C with Vysis pretreatment solution for 12 min, and treated with protease at 37 °C for 60 min. After washing, the slides were fixed with neutral‐buffered formalin, hybridized with the HER2/CEP17 probe mixture, and incubated overnight at 37 °C. After counterstaining with 4,6‐diaminido‐2‐phenylindole dihydrochloride (DAPI), FISH results were evaluated by pathologists using an epifluorescence microscope (Olympus BX51, Tokyo, Japan). An HER/CEP17 ratio ≥2.0 or an average of ≥6.0 HER2 signals was considered FISH‐positive with *ERBB2* amplification, according to ASCO/CAP 2018 guidelines.^[^ ^6^ ^]^ For interpretation, 20 tumor cells were typically counted; however, in the training cohort, 50 tumor cells were analyzed to account for HER2 ITH and optimize HER2 drPCR conditions through precise comparison with FISH results.

### drPCR

To quantify *ERBB2* CN based on the HER2/CEP17 ratio using LOAA drPCR (OPTOLANE Technologies Inc., Seoul, Republic of Korea), gDNA derived from tumor tissues (25–100 ng) or cell lines (10 ng) was mixed with HER2 (*ERBB2*)‐targeted probe (0.17 µm), reference control probe (CEP17q11.1, 0.5 µm), each of forward and reverse primers for HER2 and reference control (0.5 µm), and 2x drPCR Master Mix (15 µl, OPTOLANE) in a total 30 µL volume. For singleplex drPCR to assess the interference between HER2 and CEP17 amplicons, either HER2 or CEP17 P&P was used. Alternative reference control P&Ps (CEP17p11.1, CEP17q11.1‐distal, or CEP17p11.2) were selectively used for detection of CEP17 abnormalities. The prepared PCR mixture was loaded onto a Genotizer chip encased in a chip case and inserted into the LOAA drPCR instrument. PCR reactions were performed under the following conditions: 50 °C for 3 min, 95 °C for 15 min, 45 cycles of 95 °C for 10 s and 60 °C for 40 s. Results were analyzed using the drPCR Analyzer (Onpoint Pro v1.0.0.21, OPTOLANE). A HER2/CEP17 concentration (copies/µl) ratio of ≥1.9 was considered HER2‐positive with *ERBB2* amplification.

### NGS

For comparative evaluation of HER2 CNA among ISH, drPCR, and NGS, 53 representative tumor tissue samples from patients with breast cancer (SCHH, *n* = 40; SNUH, *n* = 13) were analyzed by targeted NGS using either Ion Torrent Genexus sequencer (ThermoFisher, Waltham, MA, USA) or Illumina NextSeq 500 sequencing system (Illumina, San Diego, CA, USA). Detailed methods for each sequencing platform are provided in the Supplementary Methods.

### Statistical Analysis

All statistical analyses were performed using SPSS (version 27.0; SPSS Inc., Chicago, IL, USA), GraphPad Prism (version 10; GraphPad Software, San Diego, CA, USA), or R (version 4.2.3). Continuous variables were assessed for normality using the Shapiro–Wilk test. Parametric tests were applied when the assumption of normality was met, otherwise non‐parametric tests were used. Data are presented as mean ± standard deviation (SD) for continuous variables and as number (percentage) for categorical variables. The sample size (*n*) for each statistical analysis is indicated in the figures, figure legends, or tables. Differences between two groups were analyzed using an unpaired Student's *t*‐test or Mann‐Whitney U test. For multiple group comparisons, one‐way ANOVA with post‐hoc Tukey's test, or the Kruskal‐Wallis test was used. Pearson or Spearman correlation coefficient was calculated to assess correlations between two factors. The association between drPCR‐based HER2 status and pCR rate was assessed using Fisher's exact test or Chi‐square test for trend, as appropriate. Binary logistic regression analysis was performed to evaluate the independent predictors of pCR in HER2‐positive patients receiving neoadjuvant therapy. ROC analysis, conducted with the R package pROC, was used to determine the optimal drPCR cutoff and evaluate its diagnostic performance compared to IHC/ISH in assessing HER2 status. KDE plots were generated using R to illustrate the distribution of HER2/CEP17 ratios measured by FISH and drPCR in cases with a drPCR‐derived ratio below 1.0 in drPCR. All *P*‐values were two‐sided, with *p* < 0.05 considered statistically significant.

## Conflict of Interest

Do Young Lee, Minsik Song, Jaewon Eom, Hyun‐Woo Song, and Hee‐Young Won are shareholders of OPTOLANE Technologies Inc. The other authors declare no competing interests. The multicenter clinical study was conducted independently, and all data analysis and interpretation were performed by clinical researchers unaffiliated with the company. All authors affirm that the results presented are unbiased and accurately reported.

## Author Contributions

H.‐J.C., S.Y.P., M.S., J.C., and Y.K. contributed equally to this work. J.‐Y.L., S.‐H.J., and H.S.R. conceived the idea and designed the study; J.‐Y.L., S.‐H.J., H.S.R., and D.Y.L. supervised and directed the project; D.Y.L., M.S., J.E., H.‐W.S., J.S. participated in the development of drPCR platform and its associated algorithms; J.‐Y.L., M.S., H.‐Y.W., H.‐J.C., J.C., and Y.K. contributed to the design and selection of primer‐probes for drPCR; S.‐H.J., H.S.R., and S.Y. chose the clinical samples, collected their clinicopathologic data by reviewing medical records, and conducted the ISH and IHC interpretation; H.P., C.C., M.‐H.O., J.‐H.L., and S.‐H.J. collected biopsy and surgical specimens from HER2‐positive patients who received neoadjuvant therapy and analyzed the predictive value of drPCR for anti‐HER2 therapy response. NGS data were analyzed by S.‐H.J. (SCHH samples) and I.J. (SNUH samples); N.H.H. analyzed the statistical significance of drPCR‐based HER2 assessment; H.S.R., S.Y.P., and M.J.S. contributed to AI‐based analysis of HER2 IHC status; J.‐Y.L., S.‐H.J., H.S.R., H.‐J.C., and S.Y.P. interpreted the results and organized the data; J.C., Y.S.K., M.J.S., D.S.K., H.K., M.K., J.E.P., Y.L., E.J., H.C., M.H., and J.‐W.N. contributed to the experiments and data interpretation; J.‐Y.L., S.‐H.J., H.S.R., D.Y.L., H.‐J.C., S.Y.P., M.S., J.S., H.‐Y.W., H.‐W.S., and J.E. contributed to writing the manuscript.

## Supporting information



Supporting Information

## Data Availability

The data that support the findings of this study are available from the corresponding author upon reasonable request.
