# Supplementary material for: Improving HER2 Diagnostics with Digital Real‐Time PCR for Ultrafast, Precise Prediction of Anti‐HER2 Therapy Response in Patients with Breast Cancer
Source: Small Methods. 2025 Sep 6;10(3):e00599. doi: 10.1002/smtd.202500599 (PMC12893271; doi:10.1002/smtd.202500599)
Supplement: Supplementary file 1 — Supporting Information [file SMTD-10-e00599-s001.docx]

Supporting Information

Improving HER2 Diagnostics with Digital Real-time PCR for Ultrafast, Precise Prediction of Anti-HER2 Therapy Response in Patients with Breast Cancer

*Hee-Joo Choi, Soo Young Park, Minsik Song, Jinhyuk Chang, YoonSik Kim, Hosub Park, Chihwan David Cha, Sohyeon Yang, Nam Hun Heo, Min Ji Song, Da Sol Kim, Hayeon Kim, Minuk Kim, Jae Eun Park, Yesung Lee, EunChae Ji, Heekyoung Chung, Ilecheon Jeong, Mineui Hong, Jin-Wu Nam, Mee-Hye Oh, Ji-Hye Lee, Jinwoo Seol,* *Hee-Young Won, Hyun-Woo Song, Jaewon Eom, Do Young Lee,^*^ Han Suk Ryu,^*^ Si-Hyong Jang,^*^and Jeong-Yeon Lee^*^*

**This supporting Information includes:**

Supplementary Methods

Supplementary References

Figs. S1 to S14

Tables S1 to S11

**Supplementary Methods**

***LOAA drPCR Platform***: The LOAA drPCR platform was developed by OPTOLANE Technologies Inc. (Seoul, Republic of Korea). The LOAA system integrates Micro-Electro-Mechanical Systems (MEMS) and complementary metal-oxide-semiconductor (CMOS) technologies within a compact, automated analyzer unit. The Genotizer chip, produced using advanced silicon MEMS fabrication techniques, comprises approximately 20,000 uniform silicon partition wells, each precisely calibrated to hold a volume of 0.94 nL. These partitions are treated with a hydrophilic surface modification to ensure efficient and uniform reagent distribution, prevent reagent evaporation, and maintain reagent stability throughout the thermal cycling process. Information about the deviation of each partition volume that may occur during chip production is recorded in a built-in Read-Only Memory (ROM). This information is used to reduce deviations between chips through precise calibration. A custom CMOS image sensor with a resolution of 1 megapixel (960 × 1080) and a pixel size of 10 µm × 10 µm was employed for fluorescence signal acquisition. The sensor featured a sensitivity of 10 V/lux·s, enabling high responsiveness under low-light conditions. A dual-channel 12-bit ADC architecture was integrated for digitization, allowing precise quantification of fluorescence intensities.

The fluorescence detection component is a CMOS sensor embedded within the chip case, positioned directly beneath the MEMS partition array of the Genotizer chip. This CMOS sensor array supports dual fluorescence detection channels, allowing simultaneous multiplex detection of two distinct fluorescent dyes. Each partition is continuously monitored by a dedicated sensor array composed of 24 pixels: 12 pixels sensitive to green fluorescence (G-pixels) and 12 pixels sensitive to red fluorescence (R-pixels). Fluorescence intensity measurements within each partition are determined by spatial averaging of the pixel signal distributions across the partition. Additionally, temporal averaging of fluorescence data collected over nine consecutive frames per PCR cycle enhances the stability and accuracy of the fluorescence measurements, significantly reducing signal noise and enhancing detection sensitivity.

Real-time fluorescence data acquired during each PCR cycle are processed using a Second Derivative Maximum (SDM) algorithm. This sophisticated method calculates cycle threshold (C_T_) values by identifying the PCR cycle at which the second derivative of the fluorescence amplification curve reaches its maximum, indicating the onset of exponential fluorescence increase. This critical inflection point accurately captures the amplification kinetics within each partition, providing precise quantification of nucleic acid targets. The distinction between amplification-positive and amplification-negative partitions is determined using both SDM and C_T_ thresholds. First, the SDM threshold is objectively established using the Histogram-based Valley Detection method. Second, the Ct threshold is determined by researchers based on the Limit of Blank (LOB) experiment to minimize background noise. A partition is classified as amplification-positive only if its SDM value is above the SDM threshold and its C_T_ value is below the C_T_ threshold. All other partitions are classified as amplification-negative.

To overcome the limited dynamic range of conventional dPCR, the LOAA drPCR system incorporates a high dynamic range (HDR) quantification algorithm, which combines the advantages of both qPCR and dPCR. The principle is as follows: (i) In the range where Poisson-based quantification is accurate (uncertainty ≤10%), conventional dPCR analysis is applied. (ii) In the high-concentration range, where Poisson estimation fails, quantification is performed using real-time C_T_ values obtained from individual partitions. In C_T_-based quantification, the relationship between the C_T_ value and target copy number (log scale) is utilized as in standard qPCR. For each partition, the number of targets (N_i_) is calculated from the observed C_T_ value (C_T,i_) using the equation:

$$C_{T,i}=a\times log\left( N_{i} \right)+C_{T,ref}$$

Where a is the slope (−3.322 for 100% PCR efficiency), and $C_{T,ref}$ is the reference C_T_ value corresponding to one target copy per partition.

Using this relationship, the total copy number in the sample can be calculated by summing over all partitions as follows:

$$\left[ Total copy number \right]=\sum_{i=1}^{n} N_{i}= \sum_{i=1}^{n} {10}^{\frac{C_{T,i}-C_{T,ref}}{a}}$$

This method enables accurate quantification even if the uncertainty of Poisson-based estimation exceeds 10% (when positive partitions exceed approximately 99.786%), where conventional dPCR becomes ineffective. Thus, by integrating real-time fluorescence data into digital quantification, the LOAA platform achieves an extended dynamic range.

***Patient Cohorts:*** A total of 398 patients with breast cancer from four independent institutions were included in this study: 363 patients for development and validation of the HER2 drPCR assay, and 35 patients for evaluating the predictive utility of drPCR in assessing anti-HER2 therapy response.

To establish a standardized HER2 testing method using drPCR, three institutions (*n* = 363) were assigned to a training cohort and two independent validation cohorts. FFPE tissue specimens were obtained from Soonchunhyang University Cheonan Hospital (SCHH, *n* = 103; Training set), Seoul National University Hospital (SNUH, *n* = 200; Validation set 1), and Chonnam National University Hwasun Hospital (CNUH, *n* = 60; Validation set 2) between 2017 and 2022. For the CNUH cohort, biospecimen and data were provided by the Korea Biobank Network. The primary treatments included radical mastectomy, modified radical mastectomy, and breast-conserving surgery with concomitant sentinel lymph node biopsy or axillary lymph node dissection. Patients who received neoadjuvant chemotherapy were excluded in these cohorts. Selected clinicopathological parameters, including age, tumor size, nuclear grade, histological grade (Nottingham system), lymphovascular invasion, lymph node metastasis, and anatomic and prognostic stage (8^th^ American Joint Committee on Cancer, AJCC), were retrieved from the electronic medical record system, as summarized in Table S3. All specimens were reviewed by pathologists who evaluated the percentage of tumor cells on selected H&E slides, and underwent prior testing for estrogen receptor (ER), progesterone receptor (PR), and HER2, reviewed in accordance with the 13th St. Gallen and ASCO/CAP guidelines.^[1-3]^

To test the potential utility of drPCR in predicting neoadjuvant anti-HER2 therapy, 35 patients previously diagnosed as HER2-positive by standard methods (IHC/ISH-based) that subsequently received neoadjuvant therapy followed by surgery were enrolled. FFPE tissue specimens of initial core biopsy specimen prior to neoadjuvant chemotherapy were obtained from Soonchunhyang University Cheonan Hospital (SCHH, *n* = 9), and Hanyang University Hospital (HYUH, *n* = 26) between 2016 and 2024. Clinicopathological data including IHC results in core biopsy specimen (HER2, ER, PR, and Ki-67), ISH results in HER2 equivocal staining cases, cTNM stage, and neoadjuvant therapy regimen were collected from medical record. The RCB score, RCB index, Miller-Payne score, number of metastatic lymph nodes, and ypTNM stage were calculated or obtained by reviewing resection specimen slides. Twenty-nine patients received neoadjuvant therapy with the TCHP (docetaxel, carboplatin, trastuzumab and pertuzumab) regimen for six cycles. Five patients received four cycles of AC (doxorubicin hydrochloride and cyclophosphamide) followed by four cycles of TH (paclitaxel and trastuzumab), and one patient received TH (docetaxel and trastuzumab) as neoadjuvant therapy.

As noted in the main Methods section, all clinical studies conducted at the participating institutions were independently approved by their respective Institutional Review Boards (SCHH, 2022-02-018; SNUH, H-2203-058-1305; CNUH, 2022-02-018; and HYUH, 2024-11-041).

***SISH:*** SISH was conducted using the VENTANA HER2 Dual ISH DNA Probe Cocktail kit on the BenchMark ULTRA automated stainer (Roche Diagnostics, Indianapolis, IN, USA) according to the manufacturer's protocols. The HER2 (DNP-labeled) and CEP17 (DIG-labeled) DNA probes were denatured at 80°C for 10 min and hybridized at 44°C for 6 h, followed by three washes at 72°C. HER2 signals were visualized as black dots using the ultraView SISH Detection Kit and silver development reagents. CEP17 signals were visualized as red dots using a mouse anti-DIG antibody and the Red ISH Detection Kit (Fast Red and Naphthol phosphate). The specimens were counterstained with Harris hematoxylin. SISH results were interpreted by independent pathologists, *ERBB2* amplification was defined according to the ASCO/CAP 2018 guidelines.^[3]^

***Cell Culture:*** MCF10A human breast epithelial cell line and different subtypes of human breast cancer cells, including MCF7 (ER+/HER2-), BT474 (ER+/HER2+), SKBR3 (ER-/HER2+), JIMT-1 (ER-/HER2+), HCC-1954 (ER-/HER2+), HCC-1569 (ER-/HER2+), MDA-MB-453 (HER2+ or TNBC), BT549 (TNBC), MDA-MB-231 (TNBC), and MDA-MB-468 (TNBC) were obtained from the American Type Culture Collection (ATCC, Manassas, VA, USA) and Korean Cell Line Bank (KCLB, Seoul, Republic of Korea). MCF10A cells were maintained in DMEM/F-12 medium (Welgene, Daegu, Republic of Korea) supplemented with 5% horse serum, EGF (20 ng mL^-1^), insulin (10 μg mL^-1^), and hydrocortisone (0.5 μg mL^-1^) at 37℃ in a humidified 5% CO_2_ incubator. MCF7, MDA-MB-231, and MDA-MB-468 cells were cultured in DMEM (Welgene) supplemented with 10% FBS; BT474, SKBR3, HCC-1954, HCC-1569, JIMT-1, MDA-MB-453, and BT549 were cultured in RPMI1640 (Welgene) supplemented with 10% FBS at 37℃ in a humidified 5% CO_2_ atmosphere.

***DNA Extraction:*** The genomic DNA (gDNA) was extracted from breast epithelial cells or cancer cell lines using QIAamp DNA Mini kit (51304, QIAGEN, Hilden, Germany) or AccuPrep Genomic DNA Extraction Kit (K-3032, Bioneer, Daejeon, Republic of Korea) as described by the manufacturer. The purified gDNA from the cell lines was incubated with EcoRI (R0101, New England Biolabs, Ipswich, MA, USA) at 37℃ for 1 h to shear the DNA into short fragments, and the restriction enzyme was inactivated at 65℃ for 20 min. Sheared gDNA was eluted in nuclease-free water.

For analysis of HER2 DNA copy numbers by drPCR in tumor tissues of patients with breast cancer, one to four 4 µm-thick FFPE sections were cut, deparaffinized, and digested with protease K overnight. Subsequently, DNA extraction and purification from FFPE tissues were conducted using PANAMAX^TM^ FFPE DNA Extraction Kit and the automated PANAMAX 16 system (PANAGENE, Deajeon, Republic of Korea; for SCHH and CNUH cohorts) or QIAamp DNA FFPE Tissue kit (QIAGEN, Germantown, MD, USA; for SNUH cohort) according to the manufacturer’s instructions. The purified DNA was quantified with Nanodrop (Thermo Fisher Scientific, Waltham, MA, USA).

***Primer and Probe Design for Digital Real-time PCR:*** To develop a new method for detection of HER2 copy number alteration (CNA) in human cancers using LOAA drPCR platform (OPTOLANE Technology Inc.), primer and probe sets (P&P) specific to HER2 gene (*ERBB2*) or DNA regions adjacent to the chromosome 17 centromere (designated as CEP17) were manually developed using freely available online tools for primer design, such as PrimerQuest (https://sg.idtdna.com/PrimerQuest; Integrated DNA Technologies Inc., Coralville, IA, USA) and OligoArchitect (http://www.oligoarchitect.com; Sigma-Aldrich, St Louis, MO, USA).

To determine the genomic region suitable for the HER2 drPCR amplicon, the mutational status of *ERBB2* (NC_000017.11; NG_007503.1) in human cancers was screened using the Catalogue of Somatic Mutations in Cancer (COSMIC) and Cancer Genome Atlas (TCGA) datasets via the UCSC Genome Browser (https://genome.ucsc.edu/). To avoid hotspot mutation sites in *ERBB2*, we selected the *ERBB2* exon 3 region, where fewer point mutations occur compared to other exons, as an optimal site for the P&P design. We confirmed that there were no point mutations within the primer and probe sequences for HER2 drPCR designed for *ERBB2* exon 3 (Figure S3A).

To design optimal CEP17 P&P targeting a focal region within chromosome 17 centromere/pericentromere with high efficiency and specificity, we searched candidate genomic regions for CEP17 P&P by exploring GC contents, highly repetitive regions, and potential CNV sites within 17p11.1-17q11.1 regions using the UCSC Genome Browser. To distinguish CEP17 abnormalities, alternative CEP17 P&Ps targeting different loci of chromosome 17 pericentromeric regions (17p11.1 to 17q11.1) or the 17p11.2 region were also designed. Among the P&Ps, CEP17q11.1 P&P was selected as the main reference control, and three other P&Ps were designated as alternative reference controls (Alt CEP17p11.1, Alt CEP17q11.1-distal, and Alt CEP17p11.2). Alt CEP17p11.2 and CEP17q11.1-distal P&Ps targeted *MTND4LP8* and *TUFMP1* pseudogene regions, and other CEP17 P&Ps were non-coding region. The information on HER2, CEP17, and alternative CEP17 P&Ps for drPCR analysis is described in Table S2.

The specificity of each primer and probe sequences was validated by NCBI nucleotide BLAST (https://blast.ncbi.nlm.nih.gov/). To confirm that PCR amplicon for HER2 or CEP17 are target-specific without any generation of non-specific PCR products, 50 ng of gDNA from breast cancer cell lines was mixed with each primer and PCR mixtures, and then real-time quantitative PCR (qPCR) for analysis of melting curves and traditional qualitative PCR for gel electrophoresis were performed using CFX opus 96 real-time quantified PCR system (Bio-Rad Laboratories, Hercules, CA, USA) and C1000 thermal cycler (Bio-Rad Laboratories), respectively. The real-time amplification curve and melting peak data were analyzed using CFX Maestro 2.2 software (version 5.2.008.0222, Bio-Rad Laboratories).

***Targeted NGS with Ion Torrent Genexus:*** For targeted NGS (panel name: OCA version 3) with Ion Torrent^TM^ Genexus^TM^, Recover All^TM^ Multi-Sample DNA/RNA Workflows (Invitrogen A26069 and A26135) were used for DNA and RNA extraction from 12 FFPE tissue samples in the SCHH cohort following the manufacturer’s recommendations using the AllTM Multi-Sample DNA/RNA workflows (Invitrogen A26069 and A26135). The DNA/RNA concentration was measured using the Qubit 2.0 Fluorimeter with Qubit DNA dsDNA BR Assay and RNA BR Assay kits (Reagecon). Nucleic acids, with a minimum of 10 ng nucleic acid input, were prepared, loaded onto the Ion Torrent^TM^ GX5^TM^ Chip, and then transferred directly onto the Ion Torrent^TM^ Genexus^TM^ sequencer. The Genexus platform was employed to process all samples, which encompassed fully automated NGS steps including library preparation, template preparation, sequencing, and bioinformatics analysis. The targeted solid-tumor panel OCA version 3 was utilized, consisting of 161 key genes, including 87 hotspot mutations, 43 focal copy number variation (CNV) gains, 48 full coding sequencing for deletion (CDS) mutations, and 51 fusion variants. After sequencing, the data was mapped and analyzed using the Genexus software integrated on the instrument. The annotation of detected mutations was performed using Oncomine Knowledgebase Reporter V.5.6.

***Targeted NGS with Illumina NextSeq 500:*** The CancerScreen Core Panel consisting of 15 target genes including *ERBB2* CNV was made based on the liquid-phase hybridization technology of Celemics, Inc. (Seoul, Republic of Korea). Capture probes were designed to bind complementary to the corresponding target regions of 15 genes. The library prep kit (Celemics) was used for Illumina sequencing by the following steps: enzymatic fragmentation (end-repair, dA-tailing), adapter ligation, and pre-PCR for indexed NGS library. To capture all target regions, NGS libraries and capture probes were hybridized using the CancerScreen Core Panel (Celemics). Target capture libraries were amplified by post-PCR to enrich sample amounts and sequenced with 2 x 150 bp paired-end reads on an Illumina NextSeq 500 sequencing system.

For alignment and variant calling of NGS results, raw FASTQ file was filtered and trimmed using Adaptor removal 2.3.3. Burrows-Wheeler aligner (BWA; version 0.7.17) MEM with default option was used to align reads to human reference genome sequence GRCh37. Sequence alignment map (SAM) file was converted to BAM format using samtools (version 1.15.1). Picard tool (version 2.27.2) was used to sort and remove duplications. GATK (version 4.2.6.1) was used to perform base quality score re-calibration and variant calling. GATK Mutect2 tumor-only mode was used to call variants following best practices. CNV values were calculated by dividing normalized regional depth of test sample with average normalized regional depth of control samples. CNV values lower than 0.25 were considered as CNV deletions, and CNV values higher than 4 were considered as CNV amplifications. In addition, CNVs of tumor cells were calculated utilizing tumor purity as follows:

(1 – [Test sample’s tumor purity]) × [Control sample’s normalize value] = [non tumor cell weight in test sample]

[Test sample’s normalize value] - [non tumor cell weight in test sample] = [tumor cell weight in test sample]

[Tumor cell weight in test sample]/( ([Test sample’s tumor purity]× [Control sample’s normalize value]) ) = [Tumor cell depth ratio comparison with normal]

[Tumor cell depth ratio comparison with normal] * 2 = [Tumor cell copy number estimated value]

***Dilution Assay for drPCR CN Test:*** To test the ability of drPCR to measure single CN of nucleic acid, the drPCR was performed with serially diluted HeLa gDNA (N4006S, NEB, Ipswich, MA, USA). *HBB* encoding hemoglobin subunit beta was chosen as a test gene, and the CN of *HBB* in the serial dilution condition was measured by using following P&Ps: forward primer, 5’-ATTGCTTACATTTGCTTCTGACACA-3’; reverse primer, 5’-GGGCAGTAACGGCAGACTTC-3’; and probe: 6FAM-5’-TTCACTAGCAACCTCAAAC AGACACCATGG-3’-SFCQ1.

***Droplet Digital PCR:*** To compare the performance of LOAA drPCR and droplet digital PCR (ddPCR) in detecting HER2 CNA, 166 FFPE tissue samples from patients with breast cancer were analyzed by ddPCR using our primer-probe sets for HER2 and CEP17. Due to differences in the fluorescence detection system between drPCR and ddPCR, the fluorescence dye of the CEP17 probe (chamel610) was replaced with Hex for ddPCR. A 20 μl PCR mixture containing 25 ng gDNA, 2X ddPCR SuperMix (1863024, Bio-Rad Laboratories), primers (0.9 μM each of forward and reverse primers) and probes (0.25 μM) of HER2 and CEP17 were prepared, and the reaction mixture was partitioned by generating droplets using the Bio-Rad QX200™ droplet generator. The generated droplets were transferred to a 96-well PCR plate and subjected to PCR in a SimpliAmp Thermal cycler (Applied Biosystems by Thermo Fisher Scientific, Carlsbad, CA, USA). Thermal cycling conditions were as follows: 95 °C for 10 min, 40 cycles of 94 °C for 30 s, 60 °C for 1 min, followed by 98 °C for 10 min, and a hold at 4 °C. After PCR, the plate was read using the Bio-Rad QX200 droplet reader, and the results were analyzed using software provided by Bio-Rad. To compare the reproducibility of ddPCR and drPCR assays, the discordant cases were replicated five to six times, and the coefficient of variation (CV%) was calculated using the formula: (standard deviation / mean) × 100.

***Analysis of Publicly Available Datasets:*** The Cancer Genome Atlas (TCGA) and Cancer Cell Line Encyclopedia (CCLE) datasets were used for analysis of genetic alterations in subsets of genes located on adjacent pericentromeric regions of chromosome 17 or genes previously recommended as a reference control for HER2 CNA analysis in patients with breast cancer and cell lines, respectively. The information on CNA, point mutation, and mRNA expression of target genes in these datasets were downloaded and analyzed via the cBioportal (http://www.cibioportal.org/). Oncoprints for visualization of genetic alterations and the correlation between *ERBB2* CNA status and mRNA expression levels were analyzed in the cBioportal.

***Sanger Sequencing:*** To define the mutation status within the sequences on CEP17 (q11.1) probe region, Sanger sequencing was performed. Briefly, the target DNA regions (250 or 526 bp) encompassing the CEP17(q11.1) probe sequences for sequencing were amplified using 2720 thermal cycler (Applied biosystem, Foster City, CA, USA), and the expected length of PCR product was separated by gel electrophoresis and purified with PCR clean up and gel extraction. The purified amplicons were then sequenced using the automated Sanger sequencing analyzer (3730xl DNA analyzer, Applied Biosystems) and analyzed using Applied Biosystems SeqStudio Sequence Analysis software (version 3.5; Applied Biosystems). The following primers were used in the amplification of PCR products and Sanger sequencing: for the 250 bp product, 5’-TGATGGGAGCAA GGAAGACC-3’ and 5’-TCCCTAGTCCTCCCCATCTG-3’; for the 526 bp product, 5’-GTAGGCAACCGCCTATTGCAG-3’ and 5’-GTTGGCTTAGATCCTGCTAACC-3’. The primers to amplify and read the target region were selected depending on the size distribution of FFPE-derived gDNA fragments. Primers for 250 bp product were used for highly degraded FFPE samples.

**Supplementary References**

1. Goldhirsch, A.; Wood, W. C.; Coates, A. S., et al., Strategies for subtypes--dealing with the diversity of breast cancer: highlights of the St. Gallen International Expert Consensus on the Primary Therapy of Early Breast Cancer 2011, Ann Oncol 2011, 22 (8), 1736-47. DOI 10.1093/annonc/mdr304.

2. Hammond, M. E.; Hayes, D. F.; Dowsett, M., et al., American Society of Clinical Oncology/College Of American Pathologists guideline recommendations for immunohistochemical testing of estrogen and progesterone receptors in breast cancer, J Clin Oncol 2010, 28 (16), 2784-95. DOI 10.1200/JCO.2009.25.6529.

3. Wolff, A. C.; Hammond, M. E. H.; Allison, K. H., et al., Human Epidermal Growth Factor Receptor 2 Testing in Breast Cancer: American Society of Clinical Oncology/College of American Pathologists Clinical Practice Guideline Focused Update, J Clin Oncol 2018, 36 (20), 2105-2122. DOI 10.1200/JCO.2018.77.8738.

4. Yao, J.; Luo, Y.; Zhang, Z., et al., The development of real-time digital PCR technology using an improved data classification method, *Biosens Bioelectron* **2022,** *199*, 113873. DOI 10.1016/j.bios.2021.113873.

5. Zhou, S.; Gou, T.; Hu, J., et al., A highly integrated real-time digital PCR device for accurate DNA quantitative analysis, *Biosens Bioelectron* **2019,** *128*, 151-158. DOI 10.1016/j.bios.2018.12.055.

**Supplementary Figures**

**
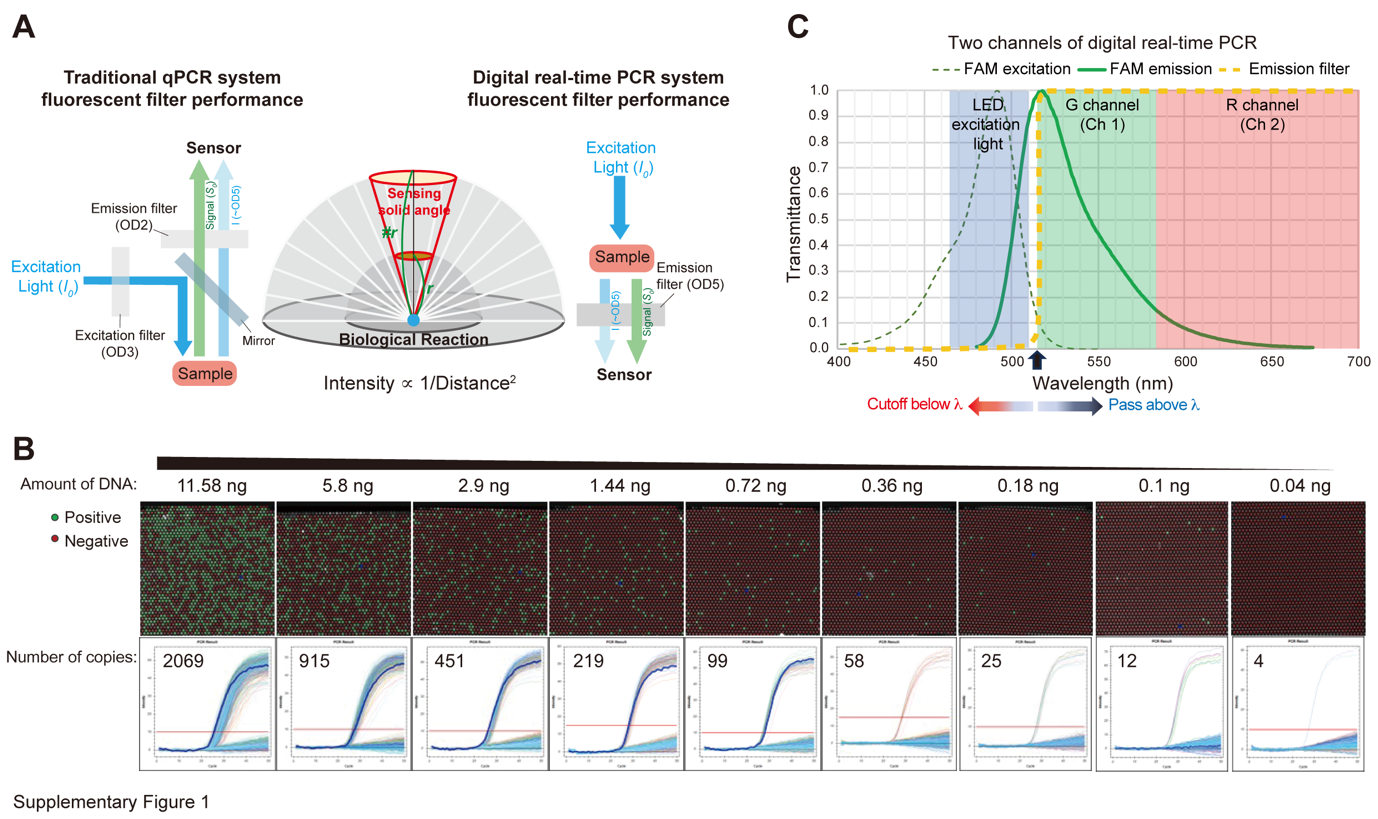
**

**Supplementary Figure 1.** Technical concept of LOAA digital real-time PCR.

(**A**) Schematic illustration of the optical system used in traditional real-time qPCR (left) and digital real-time PCR (right). Optical density (OD) is the ability of light to pass through a filter, calculated as the logarithm of the ratio of transmittance (I) to incident light (I0). (**B**) Absolute copy number (CN) analysis of LOAA drPCR. The drPCR-based *HBB* gene CN assessment of serially diluted gDNA derived from HeLa cells. In the fluorescence image on the top panel, green and red indicate positive and negative signals, respectively, and the bottom panel is the result of drPCR. (**C**) Excitation/emission profiles of fluorescence on two channels of LOAA drPCR instrument. The fluorescence filter of LOAA drPCR transmits fluorescence at 510 nm by excitation light of approximately 480 nm, and two channels can be detected through two different filters.


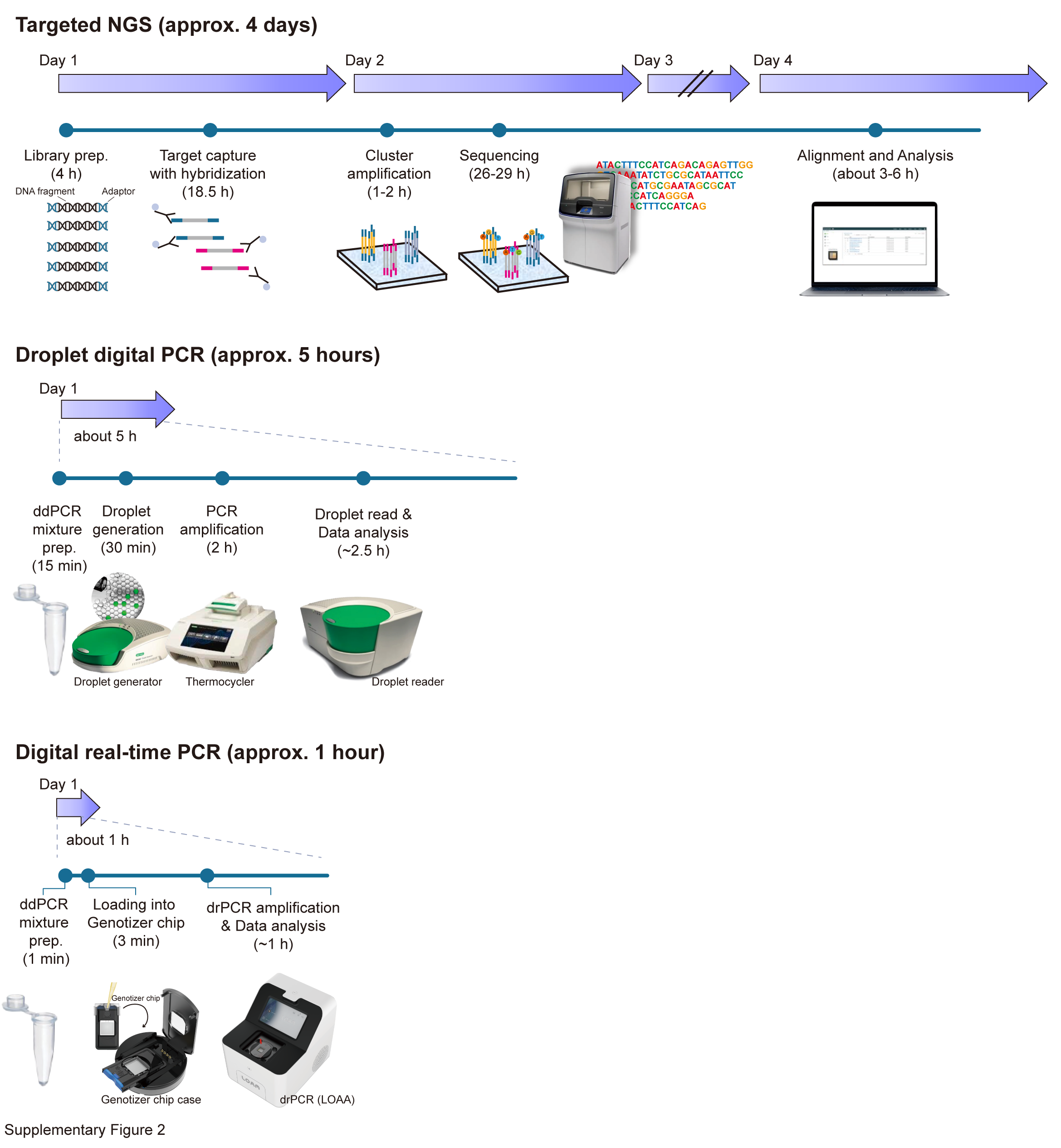


**Supplementary Figure 2.** Comparison of turnaround times among molecular diagnostic technologies for analysis of HER2 CNA.

Schematic illustration of the workflows for targeted NGS, droplet digital PCR (ddPCR), and digital real-time PCR (drPCR), comparing the turnaround times of these methods. drPCR demonstrated the simplest experimental process and the fastest turnaround time of the three technologies.


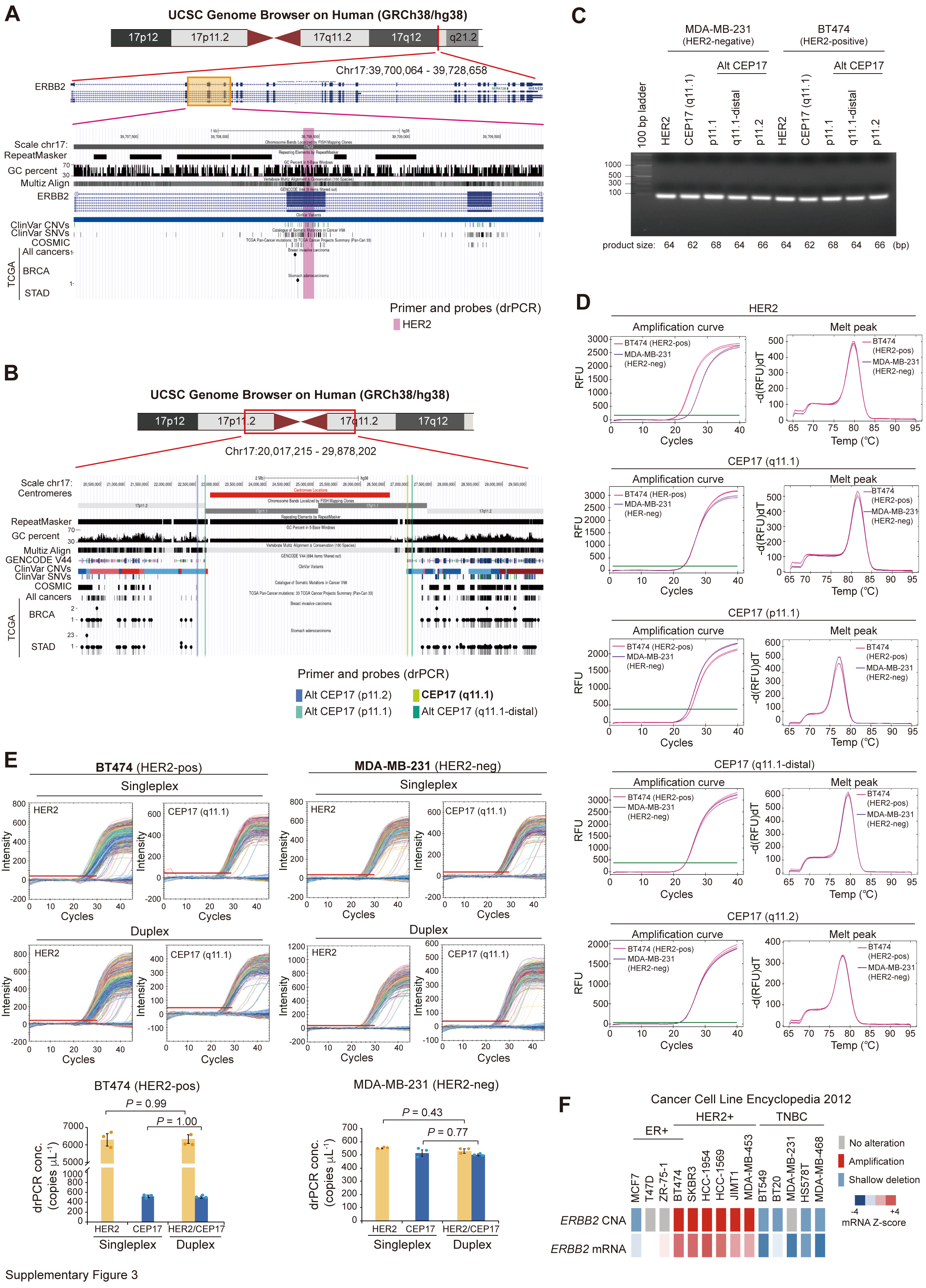


**Supplementary Figure 3.** Design and evaluation of the specificity of drPCR primer-probe sets for HER2 CNA assessment.

(**A, B**) Schematic presentation of information regarding GC contents, repetitive DNA regions, and mutational patterns of indicated genomic regions analyzed by UCSC Genome Browser (http://genome.ucsc.edu/) for appropriate and specific design of primers and probes for HER2 and CEP17 drPCR. (**C**) The gel image of PCR products shows the specificity of PCR amplicon for each target. Alt, alternative. (**D**) PCR amplification (left) and melting (right) curves in the qPCR analysis of HER2 or CEP17 (q11.1) using gDNA from BT474 and MDA-MB-231 cell lines. pos, positive; neg, negative; Temp, temperature. RFU, relative fluorescence unit; -d(RFU)dT, the negative derivative of relative fluorescence versus temperature. (**E**) Interference test between HER2 and CEP17 P&Ps by singleplex and multiplex analysis of drPCR. The data represents the means ± SD of triplicate measurements. *P*-values are calculated using one-way ANOVA with Tukey’s post-hoc test. (**F**) Copy number alteration (CNA) and mRNA expression levels of *ERBB2* in breast cancer cell lines from CCLE dataset.


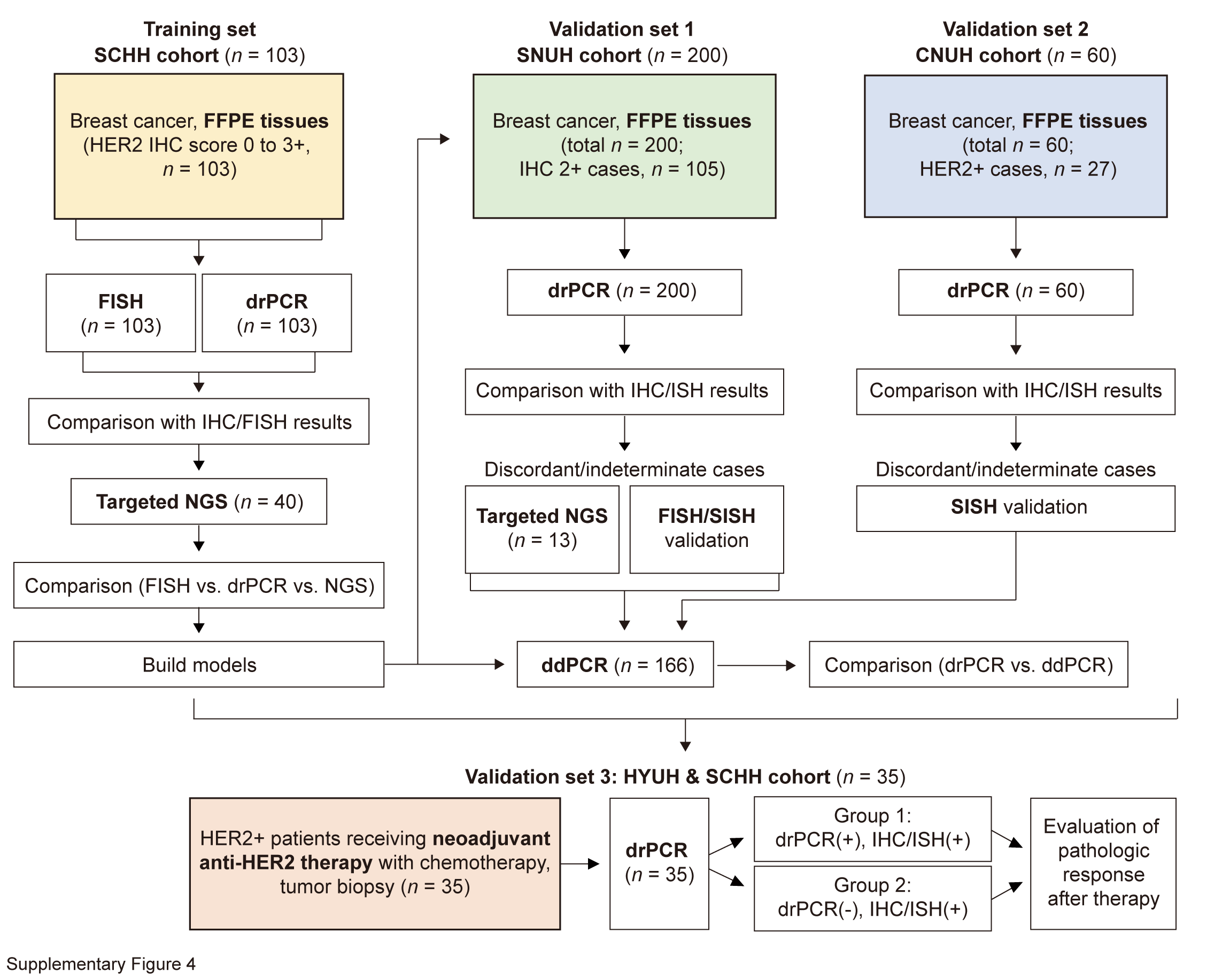


**Supplementary Figure 4.** Multicenter study design for HER2 status assessment using drPCR in patients with breast cancer.

Schematic illustration of the multicenter study model designed to verify the accuracy and clinical utility of drPCR-based HER2 assessment in patients with breast cancer. To develop and validate the drPCR-based HER2 testing method, three independent breast cancer cohorts from different institutions were assigned: a training cohort (SCHH, *n* = 103) and two validation cohorts (SNUH, *n* = 200; CNUH, *n* = 60). For comparison with drPCR results, IHC/ISH re-evaluation, targeted NGS, and ddPCR were conducted. To evaluate the predictive value of the drPCR method for anti-HER2 therapy response, drPCR was performed on biopsy specimens from 35 HER2-positive patients (SCHH, *n* = 9, HYUH, *n* = 26) who received neoadjuvant therapy followed by tumor resection. The study compared a group whose drPCR results were consistent with the existing standard test method to a group with inconsistent results. Anti-HER2 therapy responsiveness was evaluated by microscopic evaluation for the resected specimens. SCHH, Soonchunhyang University Cheonan Hospital; SNUH, Seoul National University Hospital; CNUH, Chonnam National University Hwasun Hospital. HYUH, Hanyang University Hospital.


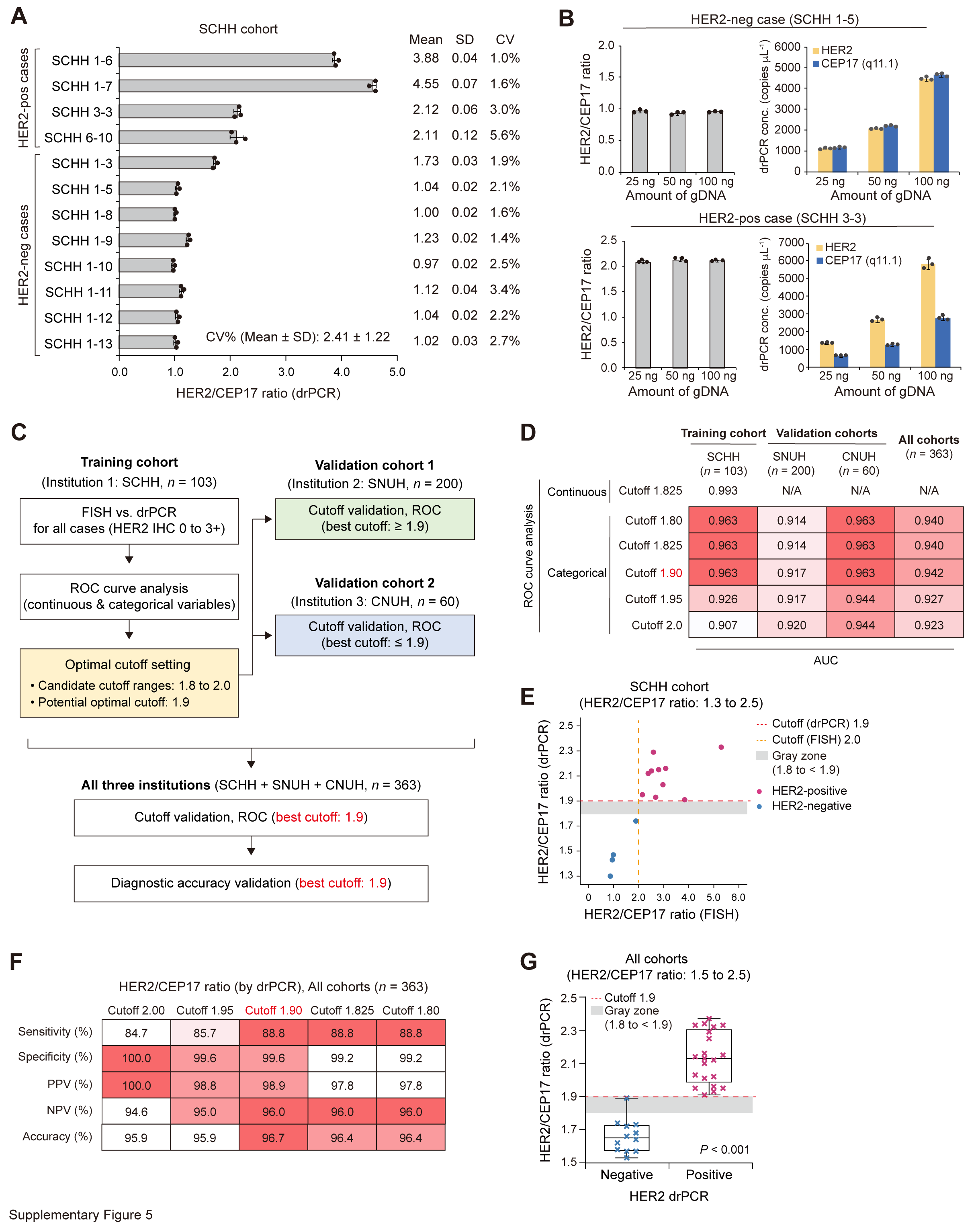


**Supplementary Figure 5.** Establishment of drPCR-based HER2 status assessment.

(**A, B**) Pre-test of drPCR assay for HER2 copy number measurement in clinical samples. The HER2/CEP17 ratio measured by drPCR in 12 cases of patients with breast cancer from the training cohort (SCHH) (A). The data represents the mean ± SD of triplicate measurements. The coefficient of variation (CV%) was calculated as the ratio of the SD to the mean, multiplied by 100. Comparison of the results of drPCR assay for HER2 and CEP17 according to the amount of gDNA from tumor tissues (B). Mean ± SD (*n* = 3). *P*-values are calculated by one-way ANOVA with Tukey’s post-hoc test. (**C**) Schematic illustration of study design to determine and validate optimal cutoff for HER2/CEP17 drPCR assay. (**D**) Graphical summary for the results of ROC curve analysis of three independent institutions (SCHH, *n* = 103; SNUH, *n* = 200; CNUH, *n* = 60) according to five candidates of cutoff values for drPCR-based HER2 assessment. (**E**) Scatter plot showing the distribution of HER2/CEP17 ratios measured by drPCR and FISH in SCHH training cohort cases with drPCR ratios between 1.3 and 2.5 (total *n* = 14; HER2-positive, *n* = 10; HER2-negative, *n* = 4). The yellow– and red– dashed lines indicate the cutoff (HER2/CEP17 ratio: 1.9 for drPCR; 2.0 for FISH). (**F**) Graphical summary of diagnostic test results according to five candidates of cutoff values for drPCR-based HER2 assessment in 363 patients with breast cancer. (**G**) Distribution of HER2/CEP17 ratios measured by drPCR (1.5 – 2.5 range), stratified by HER2 status defined by the drPCR (total *n* = 33; HER2-negative, *n* = 12; HER2-positive, *n* = 21). The *P*-value was calculated by unpaired Student’s *t*-test. Dashed red lines represent the cutoff (HER2/CEP17 ratio 1.9).

**
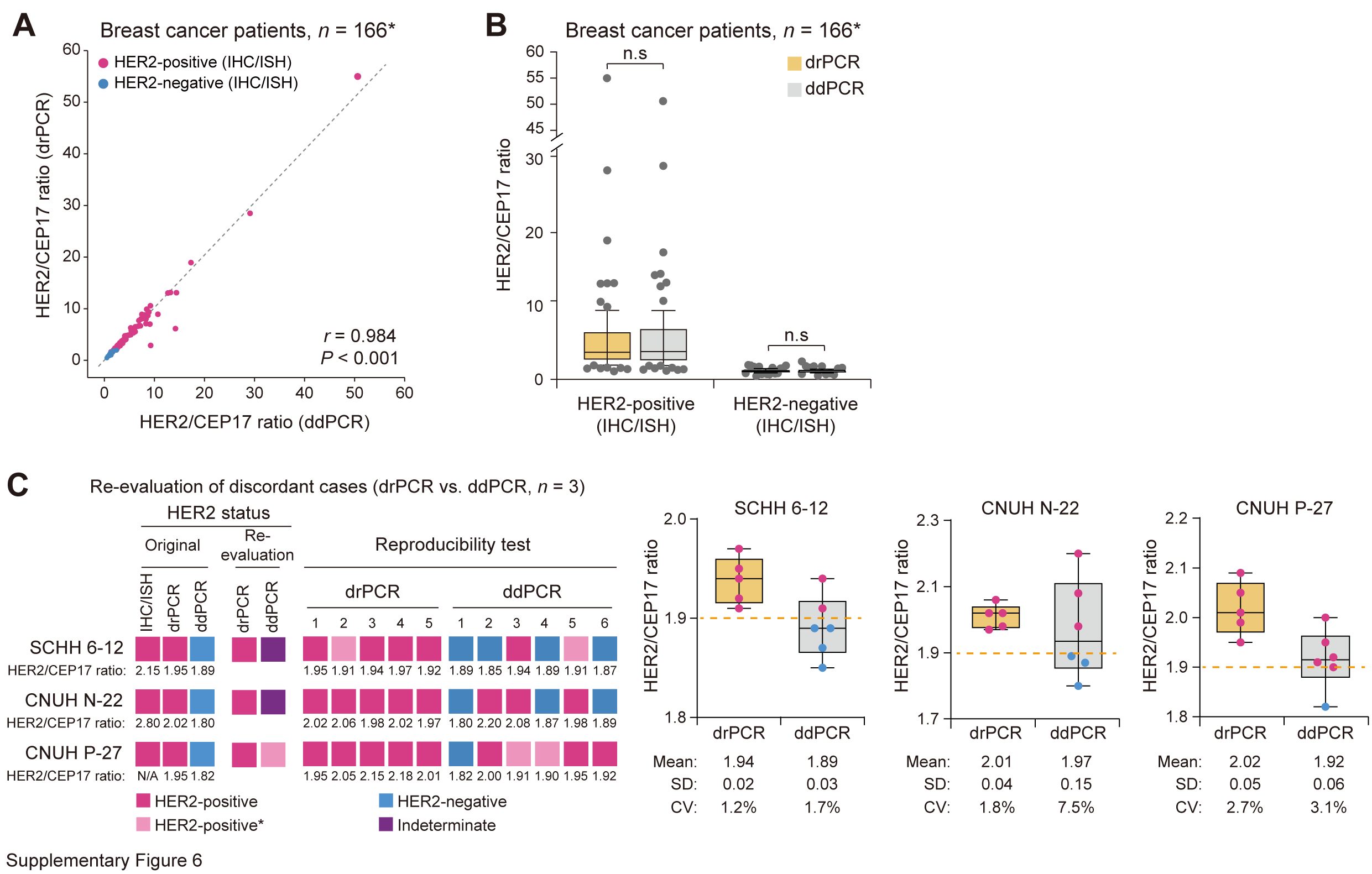
**

**Supplementary Figure 6.** Comparison of drPCR and ddPCR for HER2 status assessment.

(**A**, **B**) Scatter plot shows the correlation between HER2/CEP17 ratios measured by drPCR and ddPCR in 166 patients with breast cancer. The correlation coefficient (*r*) and *P*-value were calculated using Pearson’s correlation analysis *A total of 166 cases with diverse HER2 levels were randomly selected from all cohorts. (A). Comparison of HER2/CEP17 ratios between drPCR and ddPCR (Total *n* = 166; HER2-positive, *n* = 88; HER2-negative, *n* = 78). *P*-values were calculated using unpaired Student’s *t*-test (B). (**C**) Re-evaluation of three discordant cases between drPCR and ddPCR using reproducibility testing. Five to six replicate measurements of drPCR and ddPCR were performed for each case. Heatmap shows the re-evaluation results from each method (left), while the box plots summarize the HER2/CEP17 ratios across replicates (right). Among the cases, CNUH P-27 was classified as HER2-positive based on HER2 IHC 3+, whereas the others were HER2 IHC 2+ with ISH positivity. Dashed yellow lines represent the cutoff (HER2/CEP17 ratio 1.9). HER2-positive*, ambiguous HER2-positive; CV, coefficient of variation; SD, standard deviation.

**
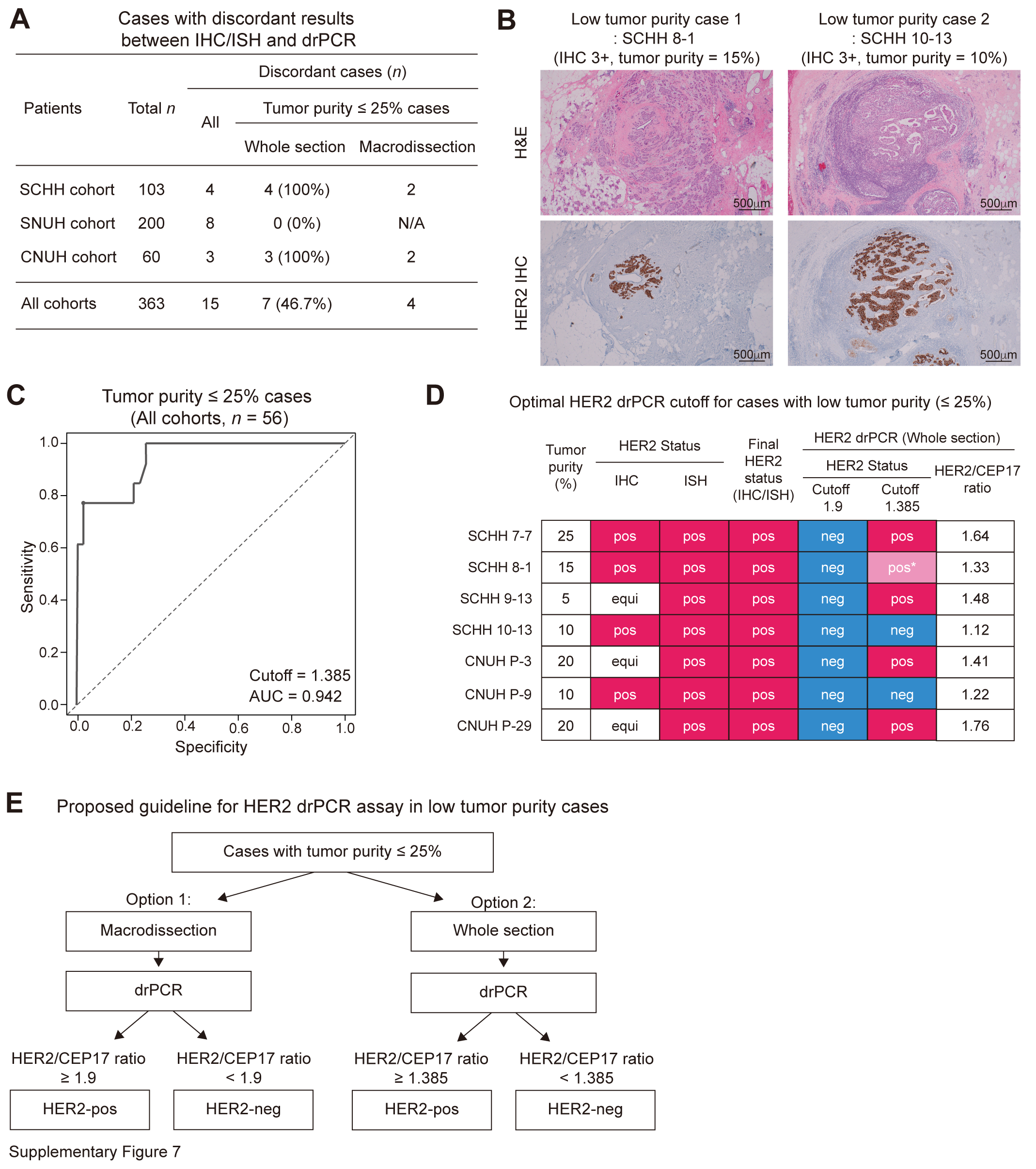
**

**Supplementary Figure 7.** Effect of tumor purity on the drPCR-based HER2 assessment.

(**A**) Cases with discrepancy between drPCR and conventional HER2 testing results (*n* = 15) are categorized according to the tumor purity. After macrodissection and subsequent drPCR analysis in the subgroup with low tumor purity (≤ 25%), the results of drPCR-based HER2 assessment in the macrodissection are compared with those from whole sections. (**B**) Representative images of the H&E (left) and HER2 (right) stained sections of low tumor purity and discordant cases. In the lower images, a significant number of lymphocytes surrounding the tumor cells can be seen. (**C**) ROC curve analysis with AUC of cases with low tumor purity (≤ 25%) using continuous values. (**D**) Heatmap showing the drPCR-based HER2 status according to optimal cutoff (HER2/CEP17 ratio 1.385) for low tumor purity cases (*n* = 7). pos, positive; neg, negative; equi, equivocal, pos*, ambiguous case. (**E**) Schematic illustration of proposed guideline of drPCR-based HER2 assessment for cases with low tumor purity (≤ 25%).

**
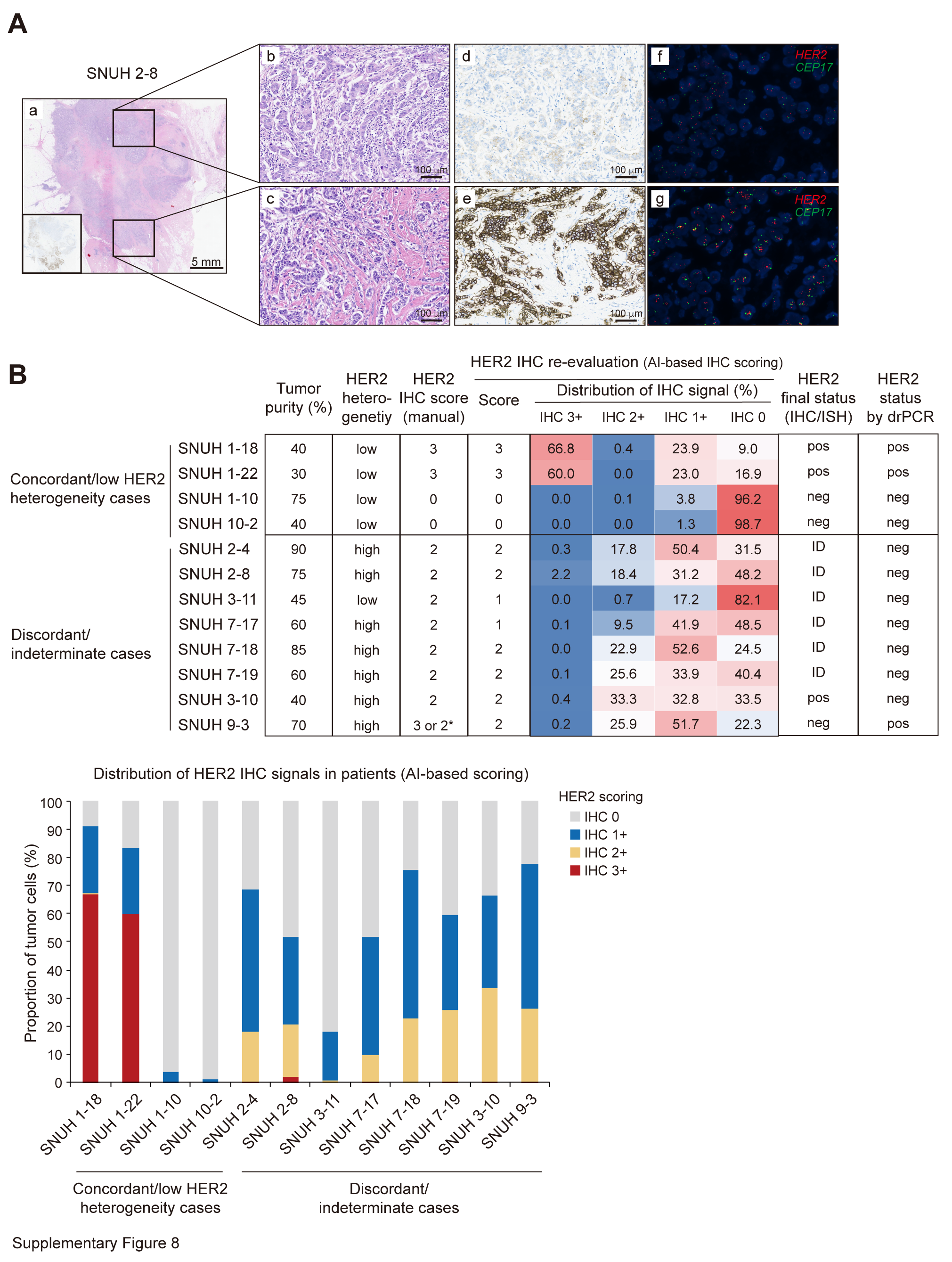
**

**Supplementary Figure 8.** Influence of intratumoral HER2 heterogeneity on drPCR-based HER2 copy number evaluation.

(**A**) Representative images of H&E staining (a, b, and c), IHC (d and e), and FISH (f and g) for HER2 testing in a case with high intratumoral HER2 heterogeneity. Bold rectangular represents IHC at x4 magnification (a, x4 magnification; b–e, x20 magnification; f and g, x60 magnification). FISH analyses display amplified *HER2* genes (red signal spots) in some tumor cells with increased intensity of HER2 protein by IHC (b, d, and f), while others with weak staining in IHC exhibit no amplification (c, e, and g), due to heterogeneous gene amplification. The green signal spots represent the centromeric region of chromosome 17 (CEP17). (**B**) The table and bar graph shows the proportion of tumor cells according to HER2 IHC scores in each case as measured by AI-based automatic algorithm for determination of the status of intratumoral HER2 heterogeneity. pos, positive; neg, negative; ID, indeterminate.

**
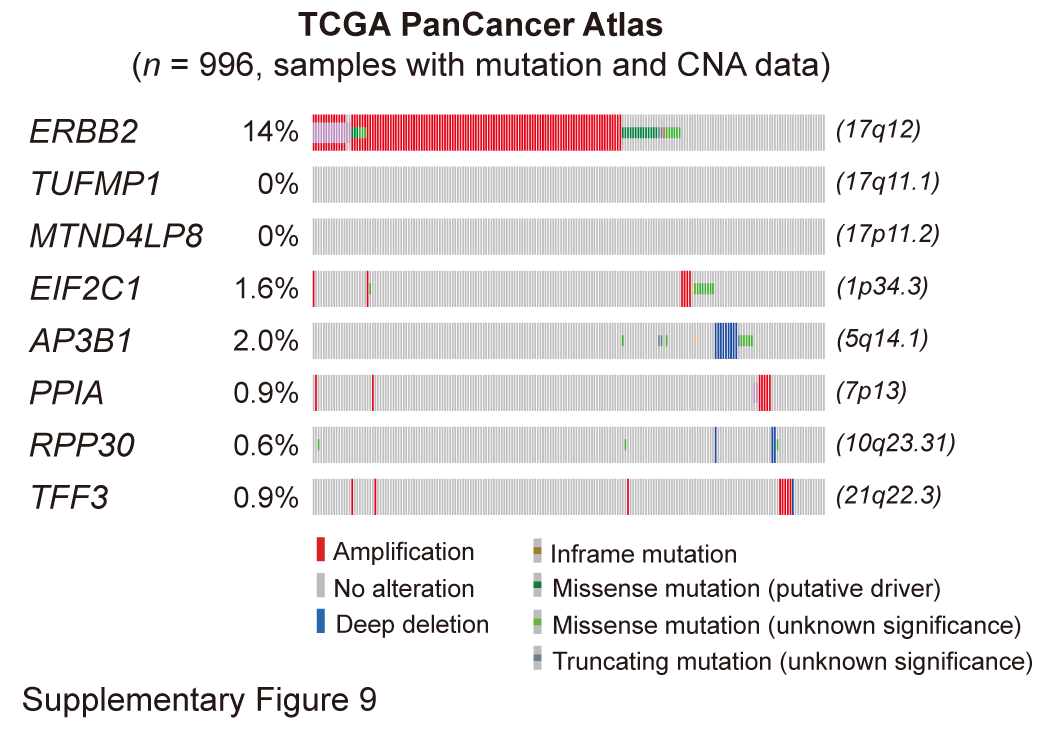
**

**Supplementary Figure 9.** Analysis of genetic alterations in reference genes previously utilized for HER2 copy number evaluation.

The patterns and frequency of genetic alterations in *EIF2C1, AP3B1, PPIA, RPP30,* and *TFF3* in patients with breast cancer from TCGA PanCancer Atlas. These genes are previously tested and recommended as reference genes for HER2 copy number assessment.

**
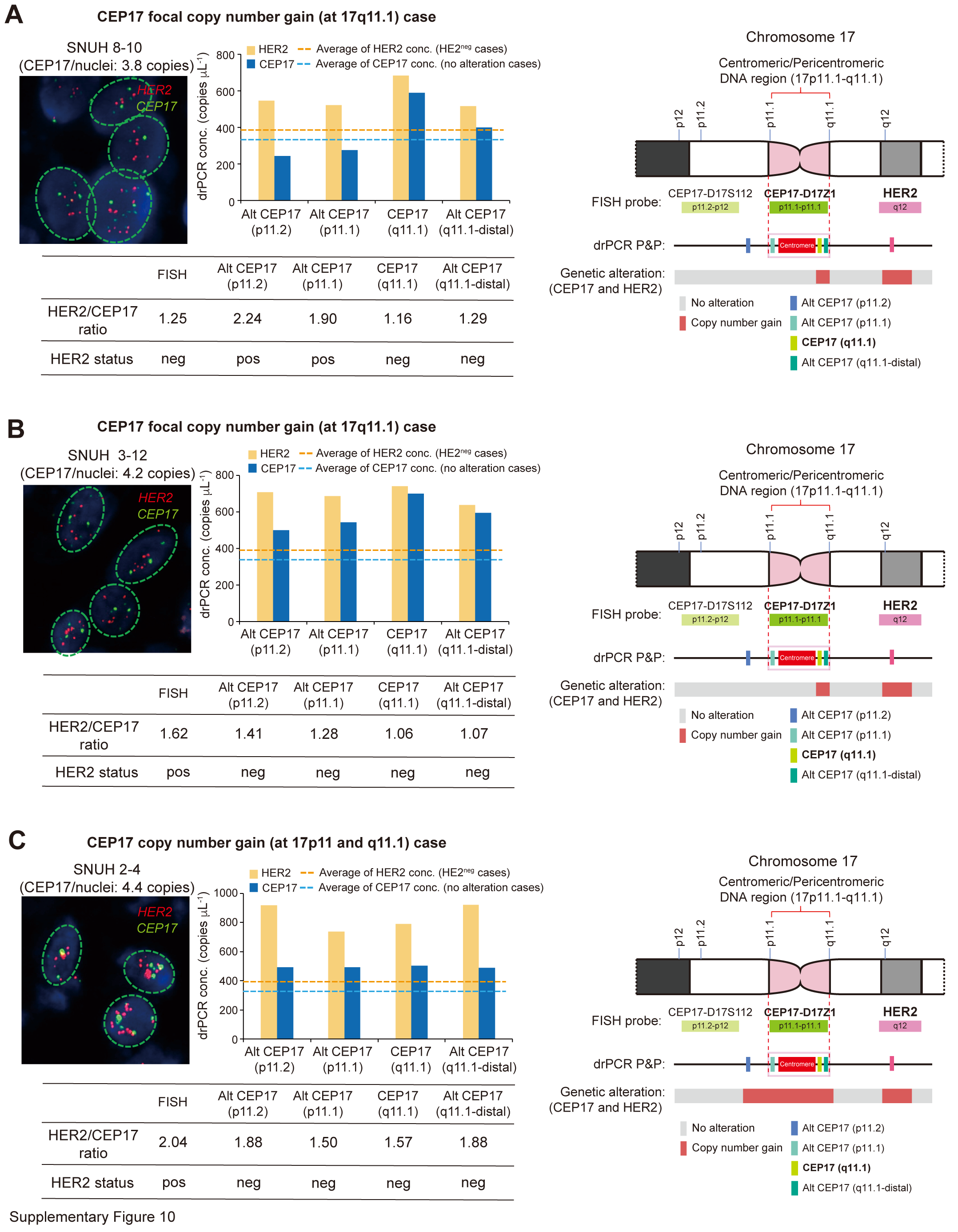
**

**Supplementary Figure 10.** Evaluation of CEP17 copy number gain by drPCR using alternative CEP17 reference controls.

(**A-C**) Representative cases with CEP17 focal copy number (CN) gain at q arm region of chromosome 17 (A-B) or both arms (C) as confirmed by drPCR analysis. *Left*, FISH analysis supports abnormal gain of CEP17 in this case, while it could not distinguish which arm region of CEP17 is altered. The nucleus showing CEP17 CN gain is circled with a dashed green line on FISH images (HER2 signals, red; CEP17 signals, green; and DAPI, blue). The bar graph and table show the results of drPCR analysis with alternative CEP17 P&Ps recognizing different loci adjacent to the p and q arms of chromosome 17 pericentromeric region. Dashed yellow and blue lines represent the average of HER2 concentration in HER2-negative cases and of CEP17 concentration in CEP17 non-altered cases of the SNU cohort, respectively, as measured by drPCR. *Right,* Schematic illustration of the pattern of genetic alterations in chromosome 17p11.2 to 17q12 and HER2 as identified by drPCR assay.

**
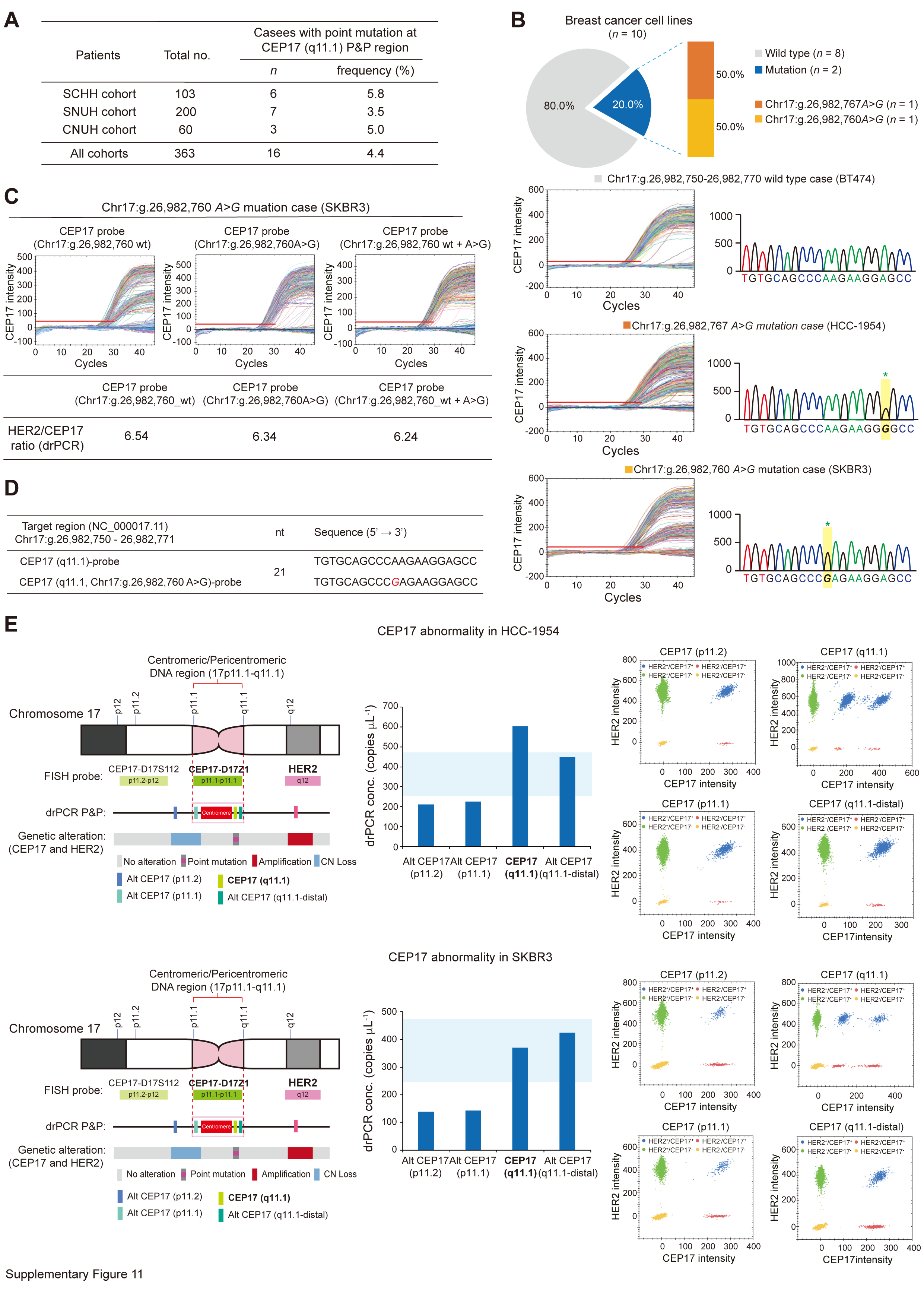
**

**Supplementary Figure 11.** Detection of complex patterns of CEP17 abnormalities in human breast cancer by drPCR using alternative CEP17 P&Ps.

**(A)** Occurrence of CEP17 point mutation at CEP17q11.1 P&P region in patients with breast cancer from indicated cohorts (*n* = 363). (**B**) Proportion of CEP17 point mutations at CEP17q11.1 P&P region in 10 breast cancer cell lines and type of point mutations confirmed by drPCR and Sanger sequencing. (**C, D**) CEP17 drPCR analysis using a modified CEP17q11.1 probe for perfect match of nucleotides by substitution of the point mutation site in SKBR3 cells harboring chr17:g.26,982,760A>G mutation. The results of drPCR by using original, modified, and 1:1 mixture of original and modified probes for CEP17q11.1 are compared (C). The information on each probe sequence is provided in the table (D). (**E**) Analysis of the patterns of CEP17 abnormalities in HCC-1954 and SKBR3 HER2+ breast cancer cell lines by drPCR assay using alternative CEP17 P&Ps. Blue area on bar graph represents the range of CEP17 concentration (copies μL^-1^) in CEP17 non-altered cases of 10 breast cancer cell lines.

**
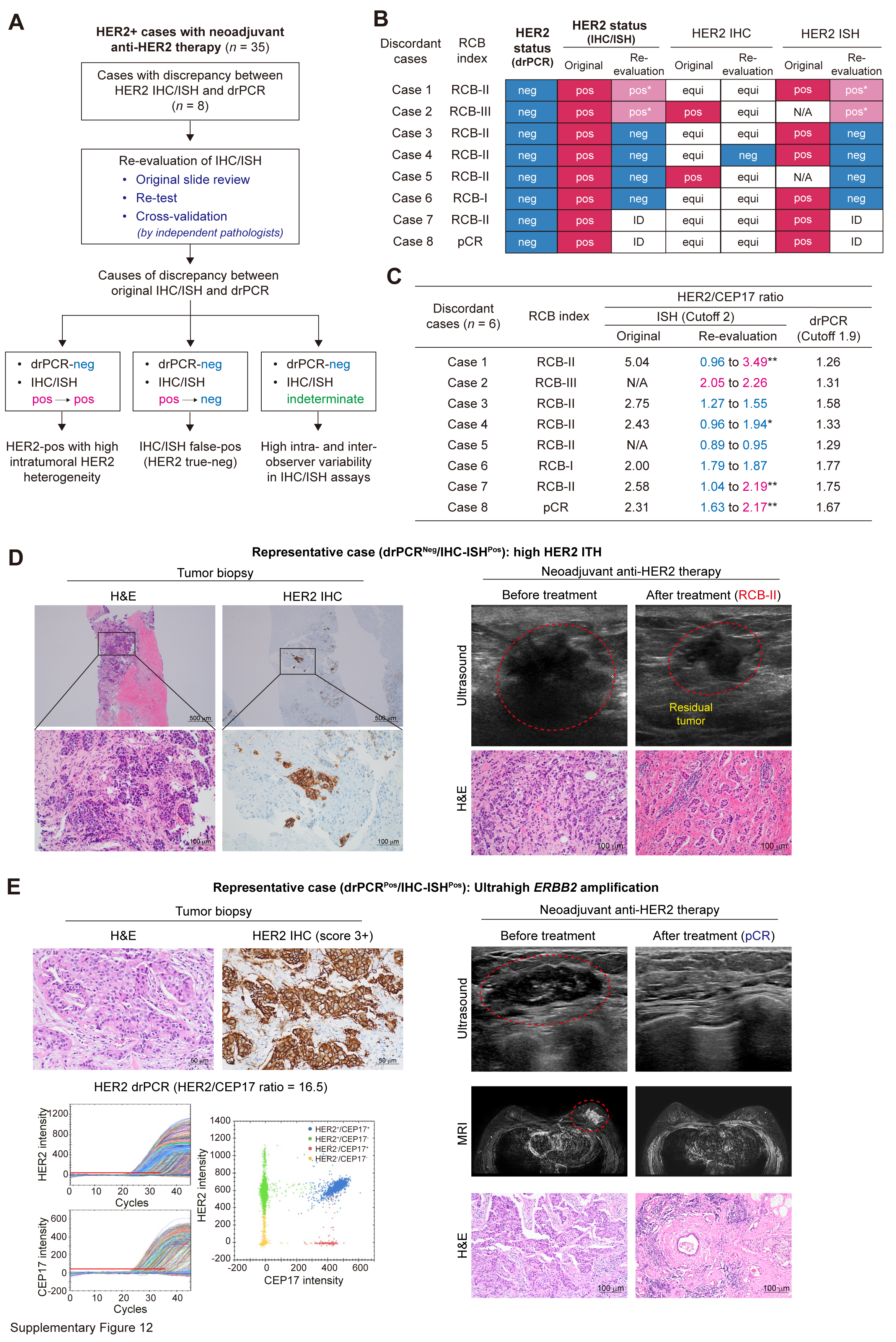
**

**Supplementary Figure 12.** Utility of drPCR-based HER2 assessment in predicting response to neoadjuvant anti-HER2 therapy.

(**A**) Workflow for re-evaluating cases with discrepancy between HER2 IHC/ISH and drPCR results in patients with breast cancer previously diagnosed as HER2-positive and treated with neoadjuvant anti-HER2 therapy (*n* = 35). High HER2 ITH, IHC/ISH false positives, and intra- and inter-observer variability in interpreting IHC/ISH results were identified as main causes of the discrepancy, linked to partial or poor response to treatment. (**B**) Results of HER2 IHC/ISH re-evaluation in HER2 drPCR^Neg^/IHC-ISH^Pos^ cases (*n* = 7). pos, positive; pos*, ambiguous-positive; neg, negative; equi, equivocal; ID, indeterminate (inter-observer disagreement); N/A, not applicable. (**C**) Re-assessment of HER2 ISH by independent pathologists in discordant cases. * and ** denote cases with high intra- and inter-observer variability in interpreting the HER2/CEP17 ratio, with HER2 status being unaffected (*) or affected (**), respectively. (**D**) Representative images of H&E, HER2 IHC, and ultrasound for a discordant case (HER2 drPCR^Neg^/IHC-ISH^Pos^) with high HER2 ITH. Ultrasound and H&E images before and after treatment reveal residual tumors in the discordant case with RCB-II (right). Red circles indicate the tumor area in the ultrasound images. (**E**) Representative images of H&E, HER2 IHC and drPCR analysis for a concordant case (HER2 drPCR^Pos^/IHC-ISH^Pos^) with ultrahigh *ERBB2* amplification. Ultrasound, MRI, and H&E images display a complete response to treatment (right). Red circles indicate the tumor area in the ultrasound and MRI images.

**
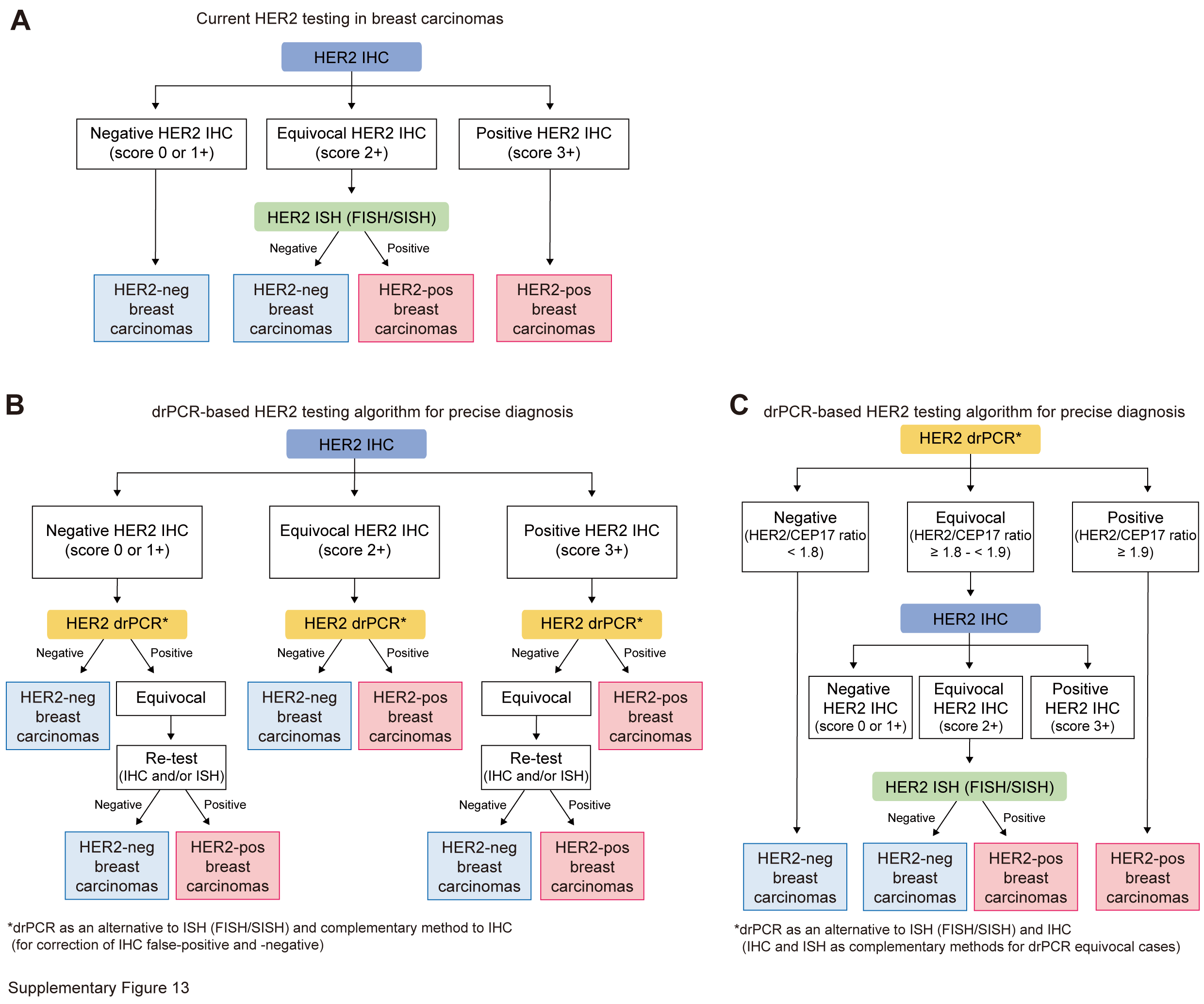
**

**Supplementary Figure 13.** Proposed HER2 testing algorithm using drPCR.

(**A**) Algorithm of current HER2 testing in breast carcinoma recommended by the ASCO/CAP guidelines. (**B**, **C**) Schematic illustrations of the proposed models for new HER2 testing algorithm by using drPCR as an alternative to ISH and complementary method to IHC (B) or as an alternative to ISH and IHC (C).

**
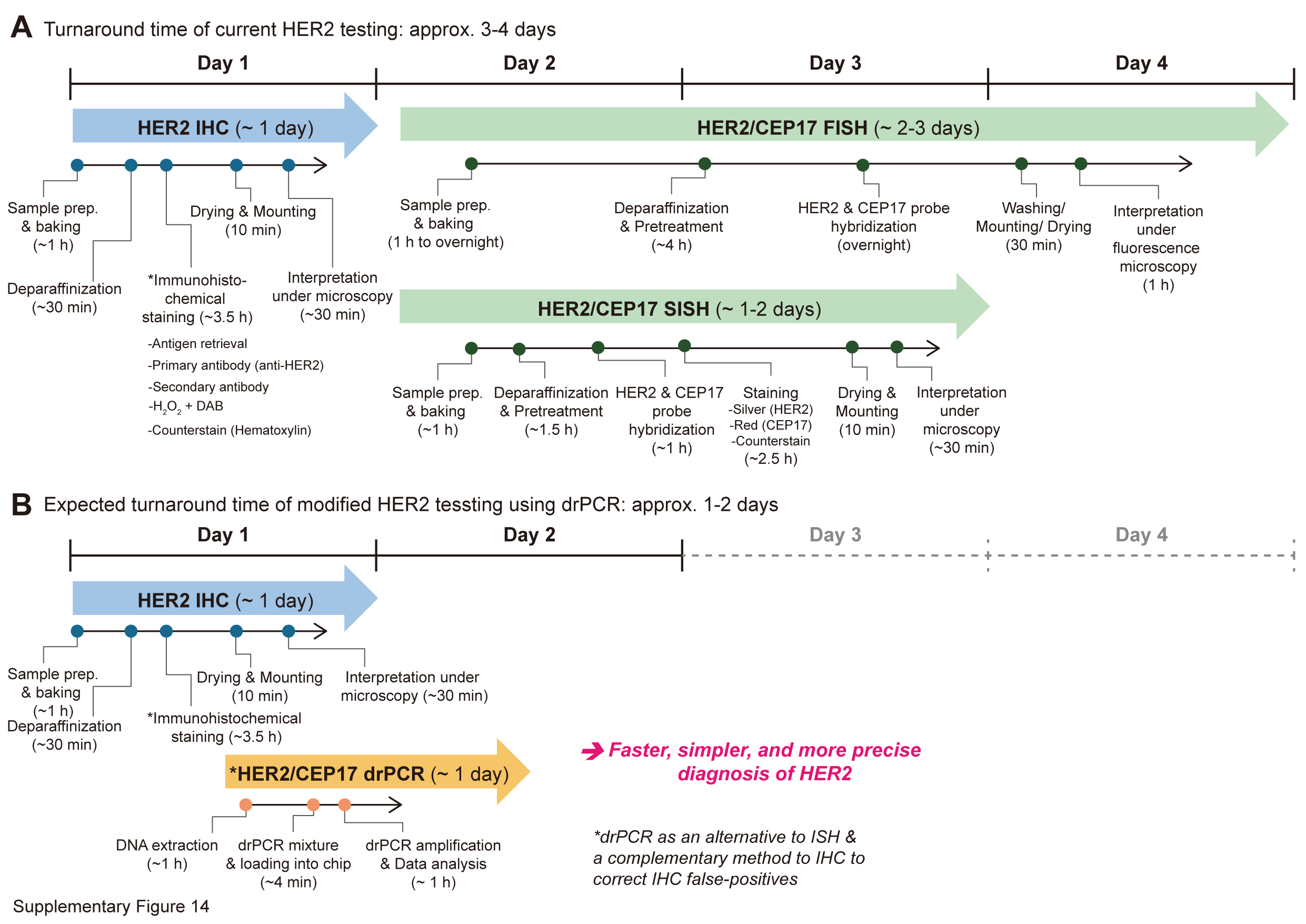
**

**Supplementary Figure 14.** Workflow of the proposed HER2 testing algorithm using drPCR.

(**A**) Standard HER2 testing workflow, with an approximate turnaround time of 3-4 days. After the initial HER2 IHC test (~1 day), cases with equivocal IHC are subjected to reflex ISH (~3 days) to confirm HER2 status according to the ASCO/CAP guidelines. (**B**) Proposed workflow for a novel HER2 testing method using drPCR as an alternative to ISH or as a complement to IHC with an expected turnaround time of under 1 day. The drPCR can be performed after or concurrently with the IHC test; the concurrent approach may expedite workflow for HER2 testing and improve accuracy by correcting IHC false positives.

**Supplementary Tables**

**Supplementary Table 1.** Comparison of LOAA drPCR with traditional dPCR platforms

|  |  | | **Droplet based** | | |  |  | **Chamber-based** | | | | | | | |  | |
| --- | --- | --- | --- | --- | --- | --- | --- | --- | --- | --- | --- | --- | --- | --- | --- | --- | --- |
| **Manufacturer** | **Bio-Rad** | | | **Stilla Technologies** | | | **OPTOLANE** | | **Standard BioTools**  **(Fluidigm Corp.)** | | **ThermoFisher** | **Qiagen** | **Real-Time PCR (Yao et al., 2022)^[4]^** | **Real-Time PCR**  **(Zhou et al., 2019)^[5]^** | | |  |
| **Platform** | QX200 ddPCR | | | Naica™ | | | LOAA drPCR | | BioMark HD | | QuantStudio Absolute Q | QIAcuity | In-house developed | In-house developed | | |  |
| **Partitioning system** |  | Droplet | | |  | | Capillary-driven | |  | Microfluidic | | | | |  | |  |
| **Dispensing method** | Droplet  (oil-emulsion based) | | | Hybrid droplet-  in-chamber | | | Self-priming | | Pressure-driven | | Heat-induced pressure | Air-displacement | unknown | Self-priming | | |  |
| **Partition volume (nL)** | 0.85 | | | •Sapphire: 0.68  •Ruby: 0.22 | | | 0.94 | | •12-inlet: 6  •48 -inlet: 0.85 | | 0.432 | •8.5K: 0.34  •26K: 0.91 | 0.9 | 5.2 | | |  |
| **Partition #/sample** | 20,000 | | | •Sapphire: 30,000  •Ruby: 17,000 | | | 20,631 | | •12-inlet: 765  •48 -inlet: 770 | | 20,480 | •8.5K: 8,500  •26K: 26,000 | 20,000 | 1120 | | |  |
| **Sample throughput/run** | 96 | | | •Sapphire: 4  •Ruby: 16 | | | 1 or 16 | | 12 or 48 | | 16 | 8 or 24 or 96 | 1 | 3 | | |  |
| **Fluorescence detection mode** | End-point | | | End-point | | | Real-time | | Real-time &  End-point | | End-point | End-point | Real-time | Real-time | | |  |
| **Dynamic range** | 5 logs | | | 5 logs | | | 8 logs | | 6 logs | | 5 logs | 5 logs | Not specified | 6 logs | | |  |
| **Throughput time (h)** | 5.5 | | | 2.5 | | | 1 | | 4 | | 1.5 | 2 | 2 (estimated) | 2 (estimated) | | |  |
| **HRM capability** | No | | | No | | | Yes | | Yes | | No | No | Yes | Yes | | |  |
| **System Integration** | No | | | No | | | Yes | | No | | Yes | Yes | No | Yes | | |  |
| **Separate modules:** | •Thermocycler  •Droplet generator  •Reader | | | •Thermocycler  •Reader | | | N/A | | External IFC controller required | | N/A | N/A | External chip loader required  (XG-TP-01, Casan) | N/A | | |  |
| **POCT suitability** | Very low | | | Very low | | | Optimal | | Low | | Fair | Fair | Fair | Optimal | | |  |
| **Equipment price** | High | | | High | | | Very low | | Very high | | High | High | Unknown | Unknown | | |  |

HRM, high-resolution melting; POCT, point-of-care test; N/A, not applicable.

**Supplementary Table 2.** List of primer-probe sets for drPCR-based detection of HER2 copy number alteration.

|  | Target region (NC_000017.11) | Product  length | nt | Sequence (5’ ⟶ 3’) | Fluorescence | Primer  /Probe  specificity |
| --- | --- | --- | --- | --- | --- | --- |
| *CNA target* | | |  |  |  |  |
| HER2-F | 39,708,411 - 39,708,474 | 64 bp | 23 | CAGCTCTTTGAGGACAACTATGC |  | specific |
| HER2-R |  |  | 21 | TGGTATTGTTCAGCGGGTCTC |  | specific |
| HER2-probe |  |  | 17 | TGGCCGTGCTAGACAAT | FAM | specific |
|  |  |  |  |  |  |  |
| *Reference control* | | |  |  |  |  |
| CEP17 (q11.1)-F | 26,982,730 - 26,982,791 | 62 bp | 20 | GACTAGGGTAGGAGGCTGAC |  | specific |
| CEP17 (q11.1)-R |  |  | 20 | GGATGCCACGATGTTTCCTC |  | specific |
| CEP17 (q11.1)-probe |  |  | 21 | TGTGCAGCCCAAGAAGGAGCC | Chamel610 | specific |
|  |  |  |  |  |  |  |
| *Alternative reference controls* | | |  |  |  |  |
| Alt CEP17 (p11.1)-F | 22,705,258 - 22,705,325 | 68 bp | 22 | CCCATCTACCCAAAGAGATGTG |  | specific |
| Alt CEP17 (p11.1)-R |  |  | 23 | TTCATGCACTGGTCTCTATTTCT |  | specific |
| Alt CEP17 (p11.1)-probe |  |  | 23 | TTCCTGCTGCTGAACCTCTTCCC | Chamel610 | specific |
|  |  |  |  |  |  |  |
| Alt CEP17 (q11.1-distal)-F | 27,083,670 - 27,083,733 | 64 bp | 17 | CATAGTGGTGGGCGACA |  | specific |
| Alt CEP17 (q11.1-distal)-R |  |  | 20 | CAAGCTCCAGGTATCACCAT |  | specific |
| Alt CEP17 (q11.1-distal)-probe |  |  | 25 | TGCTATACTCCACATGAGCCACACT | Chamel610 | specific |
|  |  |  |  |  |  |  |
| Alt CEP17 (p11.2)-F | 22,532,067 - 22,532,132 | 66 bp | 18 | AGTCTTTGCTGCCTGTGA |  | specific |
| Alt CEP17 (p11.2)-R |  |  | 23 | GACCGTATCTGTTAGAAATTGAG |  | specific |
| Alt CEP17 (p11.2)-probe |  |  | 22 | AGCAGTGGGCCTCACCCTACTA | Chamel610 | specific |

Alt, alternative; bp, base pair; CNV, copy number variation; F, forward; nt, nucleotide; R, reverse.

**Supplementary Table 3.** Clinicopathologic characteristics of patients with breast cancer enrolled in the multicenter study.

| Characteristics | All patients | Training set  (SCHH cohort) | Validation set 1  (SNUH cohort) | Validation set 2  (CNUH cohort) |
| --- | --- | --- | --- | --- |
| No. of patients | 363 | 103 | 200 | 60 |
| Age (years), mean | 54.91 (29-93) | 56.27 (33-87) | 54.84 (29-86) | 52.80 (37-93) |
| Histologic subtype |  |  |  |  |
| IDC | 333 (91.7) | 98 (95.1) | 180 (90.0) | 55 (91.7) |
| ILC | 16 (4.4) | 3 (2.9) | 11 (5.5) | 2 (3.3) |
| DCIS | 2 (0.6) | 0 (0) | 2 (1.0) | 0 (0) |
| Mucinous carcinoma | 7 (1.9) | 0 (0) | 5 (2.5) | 2 (3.3) |
| Others | 5 (1.4) | 2 (1.9) | 2 (1.0) | 1 (1.7) |
| Nuclear grade |  |  |  |  |
| Grade 1 and 2 | 207 (57.0) | 63 (61.2) | 115 (57.5)* | 29 (48.3) |
| Grade 3 | 154 (42.4) | 40 (38.8) | 83 (41.5) | 31 (51.7) |
| Histologic grade |  |  |  |  |
| Grade I | 38 (10.5) | 16 (15.5) | 13 (6.5)* | 9 (15.0) |
| Grade II | 221 (60.9) | 60 (58.3) | 134 (67.0) | 27 (45.0) |
| Grade III | 102 (28.1) | 27 (26.2) | 51 (25.5) | 24 (40.0) |
| Tumor stage |  |  |  |  |
| T1 | 214 (59.0) | 47 (45.6) | 132 (66.0)* | 35 (58.3) |
| T2 | 130 (35.8) | 48 (46.6) | 61 (30.5) | 21 (35.0) |
| T3 | 14 (3.9) | 5 (4.9) | 5 (2.5) | 4 (6.7) |
| T4 | 3 (0.8) | 3 (2.9) | 0 (0) | 0 (0) |
| N stage |  |  |  |  |
| N0 | 238 (65.6) | 69 (67.0) | 128 (64.0) | 41 (68.3) |
| N1 | 74 (20.4) | 19 (18.4) | 44 (22.0) | 11 (18.3) |
| N2 | 26 (7.2) | 12 (11.7) | 8 (4.0) | 6 (10.00) |
| N3 | 10 (2.8) | 3 (2.9) | 5 (2.5) | 2 (3.3) |
| Nx | 15 (4.1) | 0 (0.0) | 15 (7.5) | 0 (0.0) |
| M stage |  |  |  |  |
| M0 | 357 (98.3) | 102 (99.0) | 195 (97.5) | 60 (100) |
| M1 | 6 (1.7) | 1 (1.0) | 5 (2.5) | 0 (0) |
| Lymphovascular invasion |  |  |  |  |
| Yes | 197 (54.3) | 31 (30.1) | 154 (77.0) | 12 (20.0) |
| No | 166 (45.7) | 72 (69.9) | 46 (23.0) | 48 (80.0) |
| AJCC stage |  |  |  |  |
| I | 185 (51.0) | 40 (38.8) | 117 (58.5)** | 28 (46.7) |
| II | 132 (36.4) | 47 (45.6) | 63 (31.5) | 22 (36.7) |
| III | 38 (10.5) | 15 (14.6) | 13 (6.5) | 10 (16.7) |
| IV | 6 (1.7) | 1 (1.0) | 5 (2.5) | 0 (0) |
| ER status |  |  |  |  |
| Positive | 275 (75.8) | 83 (80.6) | 151 (75.5) | 41 (68.3) |
| Negative | 83 (22.9) | 20 (19.4) | 44 (22.0) | 19 (31.7) |
| PR status |  |  |  |  |
| Positive | 232 (63.9) | 68 (66.0) | 127 (63.5) | 37 (61.7) |
| Negative | 127 (35.0) | 35 (34.0) | 69 (34.5) | 23 (38.3) |
| HER2 status |  |  |  |  |
| Positive | 98 (27.0) | 27 (26.2) | 44 (22.0) | 27 (45.0) |
| Negative | 265 (73.0) | 76 (73.8) | 156 (78.0) | 33 (55.0) |

* DCIS (*n* = 2) are excluded from the categorization of nuclear grade, histologic grade, and tumor stage

** 16 cases (15 cases for Nx and 1 case for DCIS) are excluded from AJCC staging

**Supplementary Table 4.** Diagnostic accuracy of drPCR-based assessment of HER2 status in three independent breast cancer cohorts.

|  | |  | Training cohort  (SCHH, *n* = 103) | | |  | Validation cohort 1  (SNUH, *n* = 200) | |  | Validation cohort 2  (CNUH, *n* = 60) | |  | All patients  (*n* = 363) | |
| --- | --- | --- | --- | --- | --- | --- | --- | --- | --- | --- | --- | --- | --- | --- |
|  |  |  | HER2 testing (IHC/ISH) | | |  | HER2 testing (IHC/ISH) | |  | HER2 testing (IHC/ISH) | |  | HER2 testing (IHC/ISH) | |
|  |  |  | Positive (*n*) | Negative (*n*) | |  | Positive (*n*) | Negative (*n*) |  | Positive (*n*) | Negative (*n*) |  | Positive (*n*) | Negative (*n*) |
| *1. Original test** | | | | | | | | | | | | | | |
| HER2 drPCR  (Cutoff 1.9) | Positive (*n*) |  | 25 | | 0 |  | 37 | 1 |  | 25 | 0 |  | 87 | 1 |
|  | Negative (*n*) |  | 2 | | 76 |  | 7 | 155 |  | 2 | 33 |  | 11 | 264 |
| Sensitivity | |  | 92.6% | | |  | 84.1% | |  | 92.6% | |  | 88.8% | |
| Specificity | |  | 100.0% | | |  | 99.4% | |  | 100.0% | |  | 99.6% | |
| Positive predictive value (PPV) | |  | 100.0% | | |  | 97.4% | |  | 100.0% | |  | 98.9% | |
| Negative predictive value (NPV) | |  | 97.4% | | |  | 95.7% | |  | 94.3% | |  | 96.0% | |
| Accuracy | |  | 98.1% | | |  | 96.0% | |  | 96.7% | |  | 96.7% | |
| *2. Re-test*** | | | | | | | | | | | | | | |
| HER2 drPCR  (Cutoff 1.9) | Positive (*n*) |  | 25 | | 0 |  | 37 | 0 |  | 25 | 0 |  | 87 | 0 |
|  | Negative (*n*) |  | 2 | | 76 |  | 1 | 160 |  | 2 | 33 |  | 5 | 269 |
| Sensitivity | |  | 92.6% | | |  | 97.4% | |  | 92.6% | |  | 94.6% | |
| Specificity | |  | 100.0% | | |  | 100.0% | |  | 100.0% | |  | 100.0% | |
| Positive predictive value (PPV) | |  | 100.0% | | |  | 100.0% | |  | 100.0% | |  | 100.0% | |
| Negative predictive value (NPV) | |  | 97.4% | | |  | 99.4% | |  | 94.3% | |  | 98.2% | |
| Accuracy | |  | 98.1% | | |  | 99.5% | |  | 96.7% | |  | 98.6% | |

drPCR = digital real-time PCR; IHC = immunohistochemistry; ISH = in situ hybridization.

*The original test indicates that the diagnostic test was conducted by comparison with the original IHC/ISH or revised IHC results. In the IHC 3+ group, apparent IHC false-positive results were corrected based on IHC re-evaluation with ISH validation (SCHH, 7 of 26; SNUH, 8 of 38; CNUH, 4 of 26 original IHC 3+ cases).

**The re-test reflects the revised interpretation of ISH results based on repeat ISH and NGS analysis. Two indeterminate cases with ambiguous ISH and NGS results in SNUH cohort were excluded.

**Supplementary Table 5.** Comparison of drPCR with standard HER2 testing methods and NGS in the HER2 status assessment in patients with breast cancer.

| Case no.  (validation cohort, SNUH) | HER2  drPCR | | Final status  (manual IHC + ISH original) | Final status  (IHC + ISH and NGS) | 1. HER2 IHC | | | | | | | | |  | 2. HER2 ISH | | | | | |  | 3. Targeted NGS | | | |
| --- | --- | --- | --- | --- | --- | --- | --- | --- | --- | --- | --- | --- | --- | --- | --- | --- | --- | --- | --- | --- | --- | --- | --- | --- | --- |
|  |  |  |  |  | Original | |  | Re-evaluation  (manual) | |  | Re-evaluation  (AI-based) | | Final status |  | Original | |  | Re-evaluation | | Final status |  | (Cutoff 4) | | | |
|  | status | HER2  /CEP17 ratio |  |  | status | score |  | status | score |  | status | score |  |  | status | HER2  /CEP17 ratio |  | status | HER2  /CEP17 ratio |  |  | status | HER2 CNV | HER2  CN |  |
| *Discordant/indeterminate cases* | | | | |  |  |  |  |  |  |  |  |  |  |  |  |  |  |  |  |  |  |  |  |  |
| SNUH 2-4 | neg | 1.57 | pos | neg | equi | 2 |  | equi | 2 |  | equi | 2 | equi |  | pos | 2.04 |  | neg | 1.21 | ID |  | neg | diploid | 3.70 |  |
| SNUH 2-8 | neg | 1.62 | pos | neg | equi | 2 |  | equi | 2 |  | equi | 2 | equi |  | pos | 2.35 |  | neg | 0.97 | ID |  | neg | diploid | 3.70 |  |
| SNUH 3-10 | neg | 1.50 | pos | ID | equi | 2 |  | equi | 2 |  | equi | 2 | equi |  | pos | 2.66 |  | pos | 2.21 | pos |  | neg | diploid | 2.60 |  |
| SNUH 3-11 | neg | 1.10 | pos | neg | equi | 2 |  | equi | 2 |  | neg | 1 | equi |  | pos | 2.10 |  | neg | 0.86 | ID |  | neg | diploid | 3.30 |  |
| SNUH 7-17 | neg | 1.49 | pos | border-  line | equi | 2 |  | equi | 2 |  | neg | 1 | equi |  | pos | 4.71 |  | neg | 1.30 | ID |  | border-line | gain  /amp | 4.20 |  |
| SNUH 7-18 | neg | 1.37 | pos | neg | equi | 2 |  | equi | 2 |  | equi | 2 | equi |  | pos | 2.02 |  | neg | 1.53 | ID |  | neg | diploid | 2.20 |  |
| SNUH 7-19 | neg | 1.40 | pos | neg | equi | 2 |  | equi | 2 |  | equi | 2 | equi |  | pos | 3.00 |  | neg | 0.97 | ID |  | neg | diploid | 3.70 |  |
| SNUH 9-3 | pos | 1.96 | neg | ID | pos | 3 |  | equi | 2 |  | equi | 2 | equi |  | neg | 1.62 |  | neg | 0.85 | neg |  | border-line | gain  /amp | 4.30 |  |
| *Concordant/marginal amp case* | | | | |  |  |  |  |  |  |  |  |  |  |  |  |  |  |  |  |  |  |  |  |  |
| SNUH 9-4 | pos | 2.01 | pos | pos | pos | 3 |  | pos  /equi | 3 or 2 |  | equi | 2 | border-line |  | pos | 2.13 |  | neg | 1.01 | ID |  | border-line | gain  /amp | 4.50 |  |
| *IHC misinterpretation cases* | | | | |  |  |  |  |  |  |  |  |  |  |  |  |  |  |  |  |  |  |  |  |  |
| SNUH 3-20 | neg | 1.68 | neg | neg | equi | 2 |  | neg | 1 |  | neg | 1 | neg |  | neg | 1.87 |  | neg | 0.90 | neg |  | neg | diploid | 2.80 |  |
| SNUH 8-20 | neg | 1.46 | neg | neg | pos | 3 |  | equi | 2 |  | equi | 2 | equi |  | neg | 1.52 |  | neg | 1.17 | neg |  | neg | diploid | 3.50 |  |
| *HER2 CN deletion cases* | | | | |  |  |  |  |  |  |  |  |  |  |  |  |  |  |  |  |  |  |  |  |  |
| SNUH 2-10 | neg | 0.73 | neg | neg | equi | 2 |  | equi | 2 |  | neg | 1 | equi |  | neg | 0.60 |  | N/A |  | neg |  | neg | diploid | 1.70 |  |
| SNUH 5-1 | neg | 0.47 | neg | neg | equi | 2 |  | equi | 2 |  | neg | 1 | equi |  | neg | 0.51 |  | N/A |  | neg |  | neg | diploid | 2.30 |  |

AI, artificial intelligence; amp, amplification; CN, copy number; CNV, copy number variation; drPCR, digital real-time PCR; equi, equivocal; HER2, human epidermal growth factor receptor 2; ID, indeterminate; IHC, immunohistochemistry; ISH, in situ hybridization; neg, negative; NGS, next generation sequencing; pos, positive. N/A, not applicable.

**Supplementary Table 6.** Comparison of drPCR with ddPCR results in the assessment of HER2 status.

| ddPCR analysis | | Comparison to drPCR | | ddPCR | | Concordance rate %  (drPCR vs. ddPCR) |
| --- | --- | --- | --- | --- | --- | --- |
| Group | *n* |  |  | Positive  (*n*) | Negative  (*n*) |  |
| ***1. Concordant cases (IHC/ISH vs. drPCR)*** | | | | | | |
| HER2-positive (*n* = 82) | |  |  |  |  |  |
| Ultrahigh amp | 9 | drPCR | Positive (*n*) | 9 | 0 | 100%  (9 of 9) |
|  |  |  | Negative (*n*) | 0 | 0 |  |
| High amp | 33 | drPCR | Positive (*n*) | 33 | 0 | 100%  (33 of 33) |
|  |  |  | Negative (*n*) | 0 | 0 |  |
| Low-mid amp | 40 | drPCR | Positive (*n*) | 37 | 3 | 92.5%  (37 of 40) |
|  |  |  | Negative (*n*) | 0 | 0 |  |
| HER2-negative (*n* = 77) | |  |  |  |  |  |
| Non-amp | 77 | drPCR | Positive (*n*) | 0 | 0 | 100%  (77 of 77) |
|  |  |  | Negative (*n*) | 0 | 77 |  |
| ***2. Discordant cases (IHC/ISH vs. drPCR)*** | | | | | | |
| HER2-positive | 6 | drPCR | Positive (*n*) | 0 | 0 | 100%  (6 of 6) |
|  |  |  | Negative (*n*) | 0 | 6 |  |
| HER2-negative | 1 | drPCR | Positive (*n*) | 1 | 0 | 100%  (1 of 1) |
|  |  |  | Negative (*n*) | 0 | 0 |  |
| **Total cases** | 166 | drPCCR | Positive (*n*) | 80 | 3 | **98.2%**  (163 of 166) |
|  |  |  | Negative (*n*) |  | 83 |  |

HER2 status (positive and negative) was defined according to IHC and/or ISH. HER2 amplification level was determined using drPCR. Amp., amplification; ddPCR, droplet digital PCR; drPCR, digital real-time PCR; IHC, immunohistochemistry; ISH, *in situ* hybridization.

**Supplementary Table 7.** Re-evaluation of discordant cases between IHC and drPCR results in patients with an original IHC score 3+.

| Case no. | Tumor  purity (%) | HER2 IHC | | | | | | | |  | FISH/SISH validation | |  | HER2 drPCR | | Final HER2 status  (IHC/ISH and drPCR) |
| --- | --- | --- | --- | --- | --- | --- | --- | --- | --- | --- | --- | --- | --- | --- | --- | --- |
|  |  | Original | |  | Re-evaluation 1  (Manual assessment) | |  | Re-evaluation 2  (AI-based scoring) | |  | HER2/  CEP17  ratio | Status |  | HER2/  CEP17 ratio | Status |  |
|  |  | Score | Status |  | Score | Status |  | Score | Status |  |  |  |  |  |  |  |
| ***SCHH cohort*** | | | | | | | | | | | | | | | | |
| SCHH 1-5 | 80 | 3 | pos |  | 2 | equi |  | 2 | equi |  | 1.01 | neg |  | 0.98 | neg | neg |
| SCHH 1-9 | 60 | 3 | pos |  | 2 | equi |  | 1 | neg |  | 0.95 | neg |  | 1.43 | neg | neg |
| SCHH 2-9 | 40 | 3 | pos |  | 2 | equi |  | 2 | equi |  | 0.92 | neg |  | 0.99 | neg | neg |
| SCHH 2-10 | 40 | 3 | pos |  | 2 | equi |  | 2 | equi |  | 0.94 | neg |  | 1.01 | neg | neg |
| SCHH 2-11 | 30 | 3 | pos |  | 2 | equi |  | 2 | equi |  | 1.45 | neg |  | 0.99 | neg | neg |
| SCHH 8-1 | 15* | 3 | pos |  | 3 | pos |  | 0 | neg |  | 4.14 | pos |  | 1.12 | neg** | pos |
| SCHH 9-10 | 55 | 3 | pos |  | 2 | equi |  | 2 | equi |  | 0.89 | neg |  | 1.06 | neg | neg |
| SCHH 9-11 | 35 | 3 | pos |  | 2 | equi |  | 1 | neg |  | 0.84 | neg |  | 1.07 | neg | neg |
| ***SNUH cohort*** | | | | | | |  |  |  |  |  |  |  |  |  |  |
| SNUH 6-5 | 30 | 3 | pos |  | 2 | equi |  | 2 | equi |  | 1.18 | neg |  | 1.14 | neg | neg |
| SNUH 8-3 | 35 | 3 | pos |  | 2 | equi |  | 2 | equi |  | 1.24 | neg |  | 1.17 | neg | neg |
| SNUH 8-20 | 90 | 3 | pos |  | 2 | equi |  | 2 | equi |  | 1.52 | neg |  | 1.46 | neg | neg |
| SNUH 8-22 | 70 | 3 | pos |  | 2 | equi |  | 2 | equi |  | 0.88 | neg |  | 1.08 | neg | neg |
| SNUH 10-10 | 40 | 3 | pos |  | 2 | equi |  | 2 | equi |  | 1.69 | neg |  | 1.73 | neg | neg |
| SNUH 10-12 | 40 | 3 | pos |  | 2 | equi |  | 2 | equi |  | 1.36 | neg |  | 0.95 | neg | neg |
| SNUH 10-13 | 10* | 3 | pos |  | 2 | equi |  | 2 | equi |  | 1.03 | neg |  | 1.29 | neg | neg |
| SNUH 10-14 | 15* | 3 | pos |  | 2 | equi |  | 1 | neg |  | 1.11 | neg |  | 1.06 | neg | neg |
| ***CNUH cohort*** | | | | | | |  |  |  |  |  |  |  |  |  |  |
| CNUH P-3 | 20* | 3 | pos |  | 2 | equi |  | 2 | equi |  | 5.91 | pos |  | 1.65 | neg** | pos |
| CNUH P-9 | 10* | 3 | pos |  | 3 | pos |  | 2 | equi |  | 2.03 | pos |  | 1.08 | neg** | pos |
| CNUH P-15 | 25* | 3 | pos |  | 2 | equi |  | 1 | neg |  | 0.98 | neg |  | 1.07 | neg | neg |
| CNUH P-19-1 | 25* | 3 | pos |  | 2 | equi |  | 2 | equi |  | 1.20 | neg |  | 0.96 | neg | neg |
| CNUH P-21 | 40 | 3 | pos |  | 2 | equi |  | 1 | neg |  | 1.03 | neg |  | 1.09 | neg | neg |
| CNUH P-30 | 40 | 3 | pos |  | 2 | equi |  | 1 | neg |  | 1.24 | neg |  | 1.66 | neg | neg |

*Cases with low tumor purity (≤ 25%). **Discordance of HER2 status between IHC/ISH and drPCR due to low tumor purity. drPCR, digital real-time PCR; equi, equivocal; HER2, human epidermal growth factor receptor 2; ID, indeterminate; IHC, immunohistochemistry; ISH, in situ hybridization; neg, negative; no., number; pos, positive.

**Supplementary Table 8.** Effect of tumor purity on diagnostic accuracy of drPCR-based HER2 assessment (All cohorts, *n* = 363).

|  | | Cases with > 25% tumor purity  (*n* = 307) | |  | Cases with ≤ 25% tumor purity  (*n* = 56) | |
| --- | --- | --- | --- | --- | --- | --- |
|  |  | HER2 testing (IHC/ISH) | |  | HER2 testing (IHC/ISH) | |
|  |  | Positive (*n*) | Negative (*n*) |  | Positive (*n*) | Negative (*n*) |
| HER2 drPCR  (Cutoff 1.9) | Positive (*n*) | 78 | 1 |  | 9 | 0 |
|  | Negative (*n*) | 7 | 221 |  | 4 | 43 |
| Sensitivity | | 91.8 % | |  | 69.2% | |
| Specificity | | 99.6% | |  | 100.0% | |
| Positive predictive value (PPV) | | 98.7% | |  | 100.0% | |
| Negative predictive value (NPV) | | 96.9% | |  | 91.5% | |
| Accuracy | | 97.4% | |  | 92.9% | |

drPCR, digital real-time PCR; equi, equivocal; HER2, human epidermal growth factor receptor 2; IHC, immunohistochemistry; ISH, in situ hybridization.

**Supplementary Table 9.** Effect of tumor macrodissection on drPCR-based HER2 assessment.

| Case no. | Tumor purity (%) | HER2 IHC | |  | HER2 FISH/SISH | |  | HER2 drPCR | | | | |
| --- | --- | --- | --- | --- | --- | --- | --- | --- | --- | --- | --- | --- |
|  |  | status | score |  | status | HER2/  CEP17 ratio |  | Whole section | |  | Macrodissection | |
|  |  |  |  |  |  |  |  | status | HER2/  CEP17 ratio |  | status | HER2/  CEP17 ratio |
| Training set | | | |  |  |  |  |  |  |  |  |  |
| SCHH 7-7 | 25 | pos | 3 |  | pos | 5.30 |  | neg | 1.64 |  | pos* | 2.33* |
| SCHH 8-1 | 15 | pos | 3 |  | pos | 4.14 |  | neg | 1.33 |  | neg | 1.12 |
| SCHH 9-13 | 5 | equi | 2 |  | pos | 3.84 |  | neg | 1.48 |  | pos* | 1.91* |
| SCHH 10-13 | 10 | pos | 3 |  | pos | 2.58 |  | neg | 1.12 |  | neg | 1.23 |
| Validation set 1 | | | |  |  |  |  |  |  |  |  |  |
| SNUH 1-18 | 40 | pos | 3 |  | N/A | |  | pos | 6.47 |  | pos | 6.09 |
| SNUH 1-22 | 30 | pos | 3 |  | N/A | |  | pos | 3.53 |  | pos | 3.78 |
| Validation set 2 |  |  |  |  |  |  |  |  |  |  |  |  |
| CNUH P-3 | 20 | pos | 3 |  | pos | 5.91 |  | neg | 1.41 |  | neg | 1.65 |
| CNUH P-9 | 10 | pos | 3 |  | pos | 2.03 |  | neg | 1.22 |  | neg | 1.08 |
| CNUH P-29 | 20 | pos | 3 |  | pos | 6.67 |  | neg | 1.76 |  | pos* | 2.13* |

*Correction of HER2 status after macrodissection

drPCR, digital real-time PCR; equi, equivocal; HER2, human epidermal growth factor receptor 2; IHC, immunohistochemistry; ISH, *in situ* hybridization; neg, negative; pos, positive; N/A, not applicable.

**Supplementary Table 10.** Patients with breast cancer and shallow deletion of HER2 copy number detected by HER2 drPCR (HER2/CEP17 ratio ≤ 0.95).

| Case no. | HER2 drPCR | |  | HER2 ISH  validation | |  | HER2 IHC  (manual) | |  | HER2 IHC re-evaluation  (AI-based scoring) | | | | | |
| --- | --- | --- | --- | --- | --- | --- | --- | --- | --- | --- | --- | --- | --- | --- | --- |
|  | Status | HER2/  CEP17 ratio |  | Status | HER2/  CEP17 ratio |  | Status | Score |  | Status | Score | Proportions | | | |
|  |  |  |  |  |  |  |  |  |  |  |  | 3+  (%) | 2+  (%) | 1+  (%) | 0  (%) |
| *SCHH cohort (n = 12 of 103 cases; 11.7%)* | | | | | | | | | | | | | | | |
| SCHH 2-1 | neg | 0.92 |  | neg | 0.89 |  | neg | 1 |  | neg | 0 | 0.0 | 0.1 | 6.2 | 93.7 |
| SCHH 4-2 | neg | 0.87 |  | neg | 0.85 |  | neg | 0 |  | neg | 0 | 0.0 | 0.0 | 0.4 | 99.6 |
| SCHH 7-4 | neg | 0.95 |  | neg | 0.79 |  | neg | 0 |  | neg | 0 | 0.0 | 0.0 | 0.7 | 99.3 |
| SCHH 7-10 | neg | 0.95 |  | neg | 0.85 |  | neg | 0 |  | neg | 0 | 0.0 | 0.0 | 0.1 | 99.9 |
| SCHH 8-8 | neg | 0.94 |  | neg | 0.91 |  | neg | 1 |  | neg | 0 | 0.0 | 0.0 | 2.2 | 97.8 |
| SCHH 9-3 | neg | 0.92 |  | neg | 0.82 |  | neg | 0 |  | neg | 0 | 0.0 | 0.0 | 1.7 | 98.3 |
| SCHH 9-5 | neg | 0.89 |  | neg | 0.79 |  | neg | 0 |  | neg | 0 | 0.0 | 0.0 | 0.3 | 99.7 |
| SCHH 9-6 | neg | 0.95 |  | neg | 0.81 |  | neg | 1 |  | neg | 0 | 0.0 | 0.0 | 0.4 | 99.6 |
| SCHH 10-1 | neg | 0.94 |  | neg | 0.83 |  | neg | 0 |  | neg | 0 | 0.0 | 0.0 | 1.2 | 98.8 |
| SCHH 10-8 | neg | 0.86 |  | neg | 0.83 |  | neg | 1 |  | neg | 1 | 0.0 | 0.0 | 20.5 | 79.4 |
| SCHH 10-12 | neg | 0.94 |  | neg | 0.89 |  | neg | 1 |  | neg | 0 | 0.0 | 0.1 | 1.3 | 98.7 |
| SCHH 10-16 | neg | 0.86 |  | neg | 0.91 |  | neg | 1 |  | neg | 0 | 0.0 | 0.0 | 2.6 | 97.4 |
| *SNUH cohort (n = 9 of 200 cases; 4.5%)* | | | | | | | | | | | | | | | |
| SNUH 1-10 | neg | 0.72 |  | neg | 0.89 |  | neg | 0 |  | neg | 0 | 0.0 | 0.1 | 3.8 | 96.2 |
| SNUH 2-1 | neg | 0.94 |  | neg | 1.05 |  | equi | 2 |  | neg | 1 | 0.0 | 4.0 | 43.4 | 51.6 |
| SNUH 2-10 | neg | 0.73 |  | neg | 0.6 |  | equi | 2 |  | neg | 1 | 0.0 | 4.0 | 47.6 | 48.4 |
| SNUH 4-9 | neg | 0.81 |  | neg | 0.93 |  | equi | 2 |  | neg | 1 | 0.0 | 0.5 | 30.0 | 69.5 |
| SNUH 5-1 | neg | 0.47 |  | neg | 0.51 |  | equi | 2 |  | neg | 1 | 0.0 | 7.6 | 40.6 | 51.8 |
| SNUH 10-2 | neg | 0.81 |  | n.d | 0.68 |  | neg | 0 |  | neg | 0 | 0.0 | 0.0 | 1.3 | 98.7 |
| SNUH 10-12 | neg | 0.95 |  | neg | 1.36 |  | equi | 2 |  | equi | 2 | 0.1 | 41.3 | 44.1 | 14.6 |
| SNUH 10-19 | neg | 0.78 |  | n.d | 1.03 |  | neg | 0 |  | neg | 0 | 0.0 | 0.0 | 0.1 | 99.9 |
| SNUH 10-24 | neg | 0.94 |  | n.d | 1.02 |  | neg | 1 |  | neg | 0 | 0.0 | 0.0 | 1.8 | 98.2 |
| *CNUH cohort (n = 2 of 60 cases; 3.3%)* | | | | | | | | | | | | | | | |
| CNUH N-23 | neg | 0.94 |  | neg | 1.03 |  | neg | 0 |  | neg | 0 | 0.0 | 0.0 | 6.7 | 93.3 |
| CNUH N-25 | neg | 0.92 |  | n.d | |  | neg | 0 |  | neg | 0 | 0.0 | 0.0 | 0.5 | 99.5 |

drPCR, digital real-time PCR; equi, equivocal; HER2, human epidermal growth factor receptor 2; IHC, immunohistochemistry; ISH, in situ hybridization; n.d, not determined; neg, negative.

**Supplementary Table 11.** Multivariable analysis of the association between confounding variables related to treatment reponse and pCR status.

|  | Odds ratios (OR) | 95% CI | *P*-values |
| --- | --- | --- | --- |
| Age (< 55 vs. ≥ 55) | 0.32 | 0.05 – 1.97 | 0.221 |
| ER (positive vs. negative) | 0.38 | 0.06 – 2.45 | 0.306 |
| HER2 IHC (3+ vs. 2+/ISH+) | 0.83 | 0.05 – 12.97 | 0.894 |
| Treatment regimen (TCHP vs. the others) | 0.57 | 0.07 – 4.95 | 0.610 |
| HER2 drPCR (positive vs. negative) | 19.56 | 1.01 – 377.88 | 0.049* |

CI, confidence interval; drPCR, digital real-time PCR; ER, estrogen receptor; HER2, human epidermal growth factor receptor 2; TCPH : a chemotherapy regimen combining Docetaxel, Carboplatin, Trastuzumab, and Pertuzumab (6 cycles). The others include seven cases of AC-TH regimen (Doxorubicin and Cyclophosphamide (4 cycles) followed by Paclitaxel and Trastuzumab (4 cycles)) and one case of Docetaxel and Trastuzumab combination chemotherapy (6 cycles). *Statistically significant (*P* < 0.05) based on multivariate logistic regression.
